# Supplementary material for: Knowledge-guided analysis of "omics" data using the KnowEnG cloud platform
Source: PLoS Biol. 2020 Jan 23;18(1):e3000583. doi: 10.1371/journal.pbio.3000583 (PMC6977717; doi:10.1371/journal.pbio.3000583)
Supplement: S1 File — Nine appendices of supplemental commentary and additional resources describing the KnowEnG system. KnowEnG, Knowledge Engine for Genomics. (PDF) [file pbio.3000583.s001.pdf]

S1 File: Notes on the KnowEnG System

Table of Contents

Table of Contents 1
Appendix A: Infrastructure of the KnowEnG Platform 3
Overview 3
Cost Analysis 3
Resources 4
Figures 4
Figure A. KnowEnG Platform Components. 5
Appendix B: KnowEnG Platform User Experience 5
Overview 5
Figures 9
Figure B. Pipeline Home. 9
Figure C. Pipeline Setup Workflow. 10
Figure D. The Data Table. 11
Figure E. Descriptive Statistics Panel. 11
Figure F. Sample Clustering Visualization. 12
Figure G. Top Feature Selector. 12
Figure H. Gene Set Characterization Visualization Tool. 13
Figure I. Enrichment Analysis Drill Down Panel. 14
Tables 14
Table A. Resources for KnowEnG Pipelines. 14
Appendix C: Cloud Formation Template 14
Overview 14
Resources 16
Appendix D: Knowledge Network Selection Guidance 16
Overview 16
Results 17
Resources 18
Tables 18
Table B. Gene-Gene Interaction Networks. 18
Table C. Gene Set Collections. 18
Table D. AUROC by Interaction Network. 19
Table E. AUROC by Gene Set Collection Type and Network Type. 19
Table F. AUROC by Interaction Network and Gene Set Collection Type. 19
Table G. AUROC by Gene Set Collection and Network Type. 19
Appendix E: Knowledge Network Retrieval and Mapping 19
Overview 19
Resources 21
Appendix F: Reproducing Major Analyses in KnowEnG Platform 21
Overview 21
Resources 22
Figures 23
Figure J. Importing Spreadsheets for Reproducible Runs. 23
Tables 23
Table H. Analyses for Reproduction in the KnowEnG Platform. 23
Appendix G: Capabilities of the Spreadsheet Visualizer 23

Overview 23
User Interface Highlights..24
Figures 26
Figure K. Spreadsheet Visualizer Interface. 26
Figure L. Spreadsheet Visualizer Data Upload..27
Figure M. Spreadsheet Visualizer “Data-Strips”. 27
Figure N. Row Filtering and Sorting Controls. 28
Figure O. Spreadsheet Visualizer Distribution Graphs. 28
Figure P. Distribution Graph Hover Information..28
Figure Q. By Group Data Distribution..29
Figure R. Birdseye View..29
Figure S. Data Cell Hover Over..30
Figure T. Color Scale Map. 30
Figure U. Survival Curve. 31
Appendix H: KnowEnG Analysis on SB-CGC using Docker and CWL..............................31
Overview 31
Seven Bridges Cancer Genomics Cloud (SB-CGC) 31
Technologies for Importing Workflows 32
Methods 33
Run Combined, Multi-pipeline Workflow..33
Sharing and Reproducibility 33
Resources 34
Figures 34
Figure V. Editing Tool on the SB-CGC. 34
Figure W. The GSC Workflow Description. 35
Figure X. Editing the GSC Workflow. 36
Figure Y. KnowEnG Public Apps on the SB-CGC. 37
Figure Z. CWL for the GSC Tool. 38
Figure AA. Workflow for the Analysis of LUSC Subtypes. 39
Figure AB. Editing the KALS Workflow..40
Appendix I: Developing a New Analysis Pipeline for the KnowEnG System......................40
Overview 40
Command Line Tool..41
Dockerized Pipeline Tool 42
Jupyter Notebook Server 43
CWL Wrapped Tool and Seven Bridges Cancer Genomics Cloud 44
KnowEnG Platform 45
KnowEnG CloudFormation Template..47
References 50

Appendix A: Infrastructure of the KnowEnG Platform

Overview

The KnowEnG platform is a cloud-based web application. A free, public version of the platform is maintained by the Center. Users may also deploy private instances with their own Amazon Web Services (AWS) accounts using a CloudFormation Template [https://aws.amazon.com/cloudformation/aws-cloudformation-templates/], which streamlines setup (Appendix C in S1 File). The public and private versions share a common infrastructure, described here.

As shown in Figure A in S1 File, the platform software system consists of containerized components running on a cluster of virtual machines. User-submitted analyses are executed in short-running containers (those labeled Job Worker 1-N in the figure), the same as used in the SB-CGC (Appendix H in S1 File) graphical user interfaces. All other containers are long-running, always-on services that implement core functionality such as the API Server and data persistence. Both the short-running and long-running containers are orchestrated by Kubernetes, which automatically adjusts the cluster size, adding and removing nodes, as load changes. This allows the system to remain performant during periods of high demand without incurring costs for excess capacity during periods of low demand.

In the current deployments, AWS provides the cloud computing resources, which include EC2 virtual machines [https://aws.amazon.com/ec2/] as cluster nodes, S3 buckets [https://aws.amazon.com/s3/] for long-term storage, ElastiCache [https://aws.amazon.com/elasticache/] for in-memory caches, and Elastic File System [https://aws.amazon.com/efs/] for shared working storage among cluster nodes. The Center has also deployed the system outside AWS and will endeavor to remain compatible with other cloud providers.

The graphical user interface is single-page application [https://en.wikipedia.org/wiki/Single-page_application] implemented in Angular [https://angular.io/]. Visualizations are rendered using Scalable Vector Graphics [https://www.w3.org/TR/SVG/] and HTML canvas [https://html.spec.whatwg.org/multipage/canvas.html] using data fetched from the Representation State Transfer (REST) [https://www.w3.org/TR/2004/NOTE-ws-arch-20040211/#relwwwrest] API.

The platform adopts best practices and standards-based measures for security. Among these are OAuth 2.0 [https://oauth.net/2/] for single sign-on, JSON Web Tokens [https://jwt.io/] for authentication and authorization, Transport Layer Security [https://tools.ietf.org/html/rfc8446] to encrypt all traffic over the open Internet, and AWS Virtual Private Clouds [https://aws.amazon.com/vpc/] for network security.

Cost Analysis

The cloud-computing costs of running the public platform instance can be decomposed into three parts:

1. **Baseline compute**. This is the cost of running the cluster at is smallest size, during periods of low utilization, when it consists of four EC2 nodes. At current AWS on-demand prices as of May 2019, the total baseline compute cost is $0.4904 per hour, or about $364.86 per month. This cost could be reduced by (a) switching from on-demand nodes to Reserved Instances, which require a minimum one-year commitment, and (b) tuning node sizes, possibly reducing the responsiveness of the system during periods of low utilization.
2. **Burst compute**. This is the cost of adding extra nodes to the cluster during periods of high utilization. We currently allow the system to add up to ten m5.4xlarge [https://aws.amazon.com/ec2/instance-types/] nodes, each of which has 16 CPUs and 64 GiB RAM and costs $0.768 per hour.
3. **Storage**. This is the cost of storing cluster data on the Elastic File System. AWS charges $0.30 per gigabyte per month, and total cost to the Center has averaged $32.21 per month for September 2018 through February 2019. This cost could be reduced by (a) migrating more runtime data from Elastic File System to S3 buckets, which are billed at a lower rate, and (b) tightening data-retention policies that govern the persistence of user uploads and analyses.

Resources

KnowEnG Platform Components

Docker Image for API Server [https://hub.docker.com/r/knowengdev/nest_flask/]
Docker Image for Job Queue [https://hub.docker.com/r/knowengdev/nest_jobs/]
GitHub Repository [https://github.com/KnowEnG/platform]

Figures

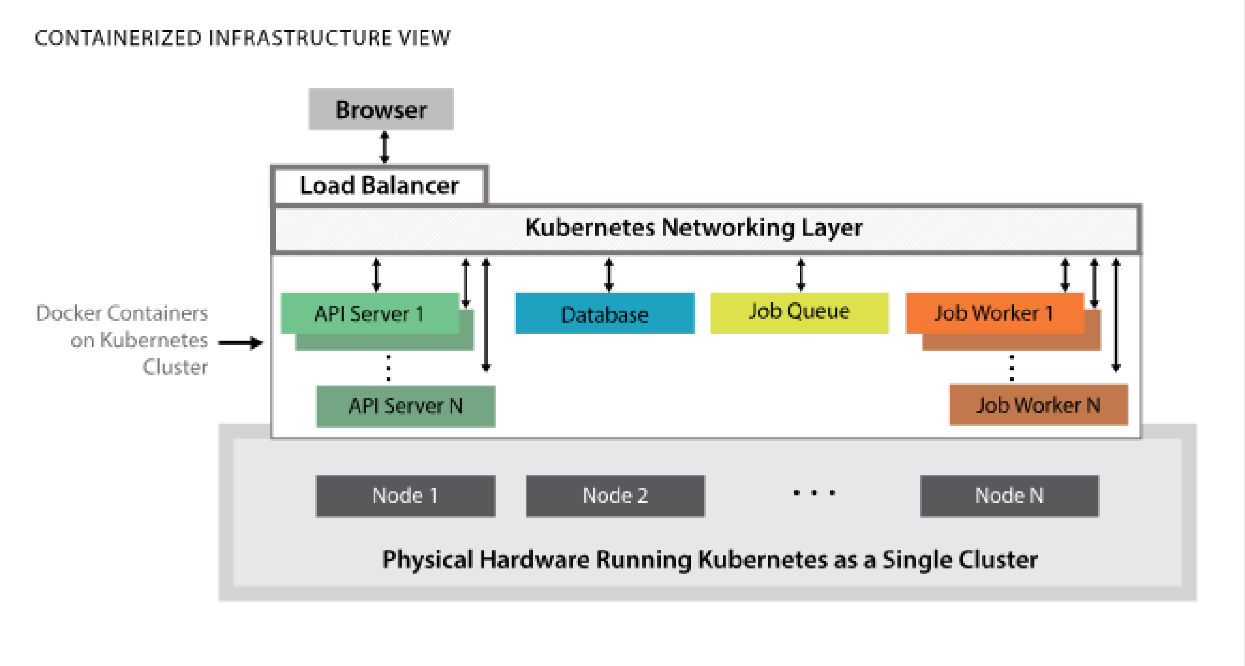

Figure A. KnowEnG Platform Components.
Components are isolated in Docker containers, indicated by the colored boxes. The containers are run by a Kubernetes orchestration system over multiple nodes. The number of cluster nodes automatically adjusts in response to user traffic.

Appendix B: KnowEnG Platform User Experience

Overview

KnowEnG is a web-based platform designed to provide biologists with an **intuitive user interface** enabling the execution of complex analytical processes, as well as **interactive visual displays** supporting the study and evaluation of results. The KnowEnG team believed that a carefully considered UI was critical to wide adoption of the platform by people who do not have experience in analytics. Toward that end, we brought together a variety of expertise and perspectives including, biomedical scientists, data scientists, software engineers and designers. Through these collaborations, we identified platform features that (1) addressed the needs of the scientists, (2) harnessed the power of the analytics, and (3) fell within resource and time constraints. Our solutions incorporate information design principles that we have used successfully in other domains [1, 2]. These include providing (1) qualitative overviews coupled with quantitative details, (2) efficient comparison methods, (3) relevant context and evidence for confirmation, and (4) illustrations of relationships between data types [http://vi-bio.ncsa.illinois.edu/next-gen-sequencing-results-visualization.html].

Technology Overview

The KnowEnG Platform interfaces were designed using Sketch App user interface design tool and InVision prototyping and style markup tool. InVision, specifically, provided developers with access to all of the interface mockups and was used during all stages of development to collaborate on design software feature decisions with all members of the development team.

The technology stack includes a single-page web application in the Angular framework, backed by a REST API in Python Flask with Redis, PostgreSQL, and Amazon S3 for data persistence. The compute cluster is managed by Kubernetes and automatically adjusts to user load by adding or removing Amazon EC2 virtual machines. The environment employs best practices for security, including OAuth 2.0 authentication and virtual private clouds. A free, public instance of the platform is administered by KnowEnG staff. Users may easily deploy private instances of the platform to their own AWS accounts using KnowEnG’s Cloud Formation Template.

Workflow

The KnowEnG platform is geared to the biologist and provides easy access to cutting edge analytical methods required to study genomic data effectively. The platform presents a process that includes easy data upload and selection, effortless algorithm parameter tuning for complex machine learning experiments, and sophisticated visualization tools for evaluating data and

studying results. It provides guidance on preparing data, choosing appropriate pipelines, and adjusting parameters. It provides interactive, visual methods for filtering and comparing data. Moreover, as users move through the workflow, contextually relevant help content is available for reference.

Navigation

The platform has three conceptual areas: *Analysis Pipelines, Data,* and *Support*. Clicking on *Analysis Pipelines* provides information about each pipeline including a description of the methods, common uses, input requirements, and links to tutorials and video clips. Here, the user can start a pipeline (Figure B in S1 File). Clicking on *Data* provides users with persistent access to their uploaded study data and resulting data produced by the executed pipelines. KnowEnG also provides example data for each pipeline for demonstration. *Support* houses contacts, documentation/training, and links to KnowEnG relevant publications and their associated data.

Launching Analysis Pipelines

The KnowEnG platform offers pipelines for common genomics analysis tasks. Current pipelines include Sample Clustering, Feature Prioritization, Gene Set Characterization, Signature Analysis, and Spreadsheet Visualization. Each pipeline provides common algorithmic approaches, and some include novel techniques that incorporate the use of prior knowledge. Selecting "Start Pipeline" for any one of these initiates a step-based setup tool. The setup flow is generally to upload/select data, and then to make parameters choices (Figure C in S1 File). The last step displays all selections for review and allows the user to add notes to keep track of data experiments. The analysis is the launched and runs automatically in the cloud. Results of the pipeline appear in the Data section. Clicking on any completed pipeline run displays reference information about the run and the option to study results in the visualization view or to download a zip file of the results to the local computer. Results of some pipelines can also be used as data inputs to certain other pipelines (Figure D in S1 File).

Viewing the Results

All pipelines have interactive visualizations that help the researcher review and study the results. The visuals provide high-level understanding of the data, and multiple features allow the user to filter, sort, and compare. Quantitative details are provided via drill-down methods. Details of each visualization are not discussed in this supplement, but a few selected, novel features are described below:

Example Features

<u>Distribution Graphs:</u> Most of the custom visualizations provide descriptive statistics that help users understand the distribution of their data across user-selected variables (often phenotypic). These are available by hovering over or clicking on the elements in the visualization. In the Sample Clustering visualization, the small graph shows the distribution of both continuous and categorical data. Hovering over graph shows the percent of data and quantity of samples at that point (or in the bin). Clicking on the single row heatmap opens a more detailed view of the data distribution, including a breakdown of the distribution based on the "group columns by" variable

(Figure E in S1 File). This is recalculated on the fly as the user explores other “group columns by” variables.

Clustering: The visualization for Sample Clustering provides an interactive heatmap displaying the samples (e.g. patients) as columns and the data (e.g. gene expression values) as rows. The columns are grouped by the cluster assignment, and sorted within each grouping by each sample’s silhouette score (the measure of how well the sample fits in that cluster). The rows are sorted by the strength of the statistical association between the row and the clustering assignments. The user can re-sort the rows by variance of data as well. In cases where the researcher included “bootstrapping” in the clustering analysis, a second heatmap is displayed below the other. This heatmap displays the same samples as columns and always stays aligned with the heatmap above. The rows duplicate the same samples providing a matrix that indicates how frequently each pair of samples was assigned to the same cluster, as the algorithm performed extensive clustering operations. This is referred to as the “consensus matrix”.

The single row heatmaps at the top of the screen allow the user to re-group and re-sort the columns by other features of interest, including phenotypic features. Additional single row heatmaps can be displayed at the bottom and their columns are also aligned to the same samples (Figure F in S1 File). This allows for fluid comparison across these different heatmaps on the same screen allowing users to get a sense of the features that are guiding and correlated with the current grouping. For more information about features, see Appendix G of S1 File.

Top Features Selector: The Top Features Selector appears in several visualization to help users control the number of features being displayed in the visualization. Rather than arbitrarily selecting top 10 or 100 features, the user can use this tool to select “top feature” thresholds based on seeing the distribution of the scores from highest to lowest. Researchers can see at a glance if there is small set of high scoring features, or if there is a gradual transition between high and low scoring (Figure G in S1 File). This not only allows users to select what is most interesting, but also affords a peek into the distribution of feature scores across all the data.

Filtering and Sorting: The visualizations in KnowEnG incorporate multiple methods for extensive sorting and filtering. This high-level of interactivity supports the efficient study of results. One example is the Gene Set Characterization visualization, which helps researchers see how their genesets overlap with the extensive public geneset collections that make up the Knowledge Network. The results are displayed as a heatmap where the researcher’s gene sets are rows and the public sets are columns. These columns can be grouped by class and sorted alphabetically, or by how well they score across the user gene sets. The cells display the color associated with the score and are clickable to access detailed information. The control panel on the left organizes all the public gene sets and supports refined filtering based on the ontology of each class of gene sets (Figure H in S1 File). Quantitative information about the level of enrichment is integrated in this panel helping the user more quickly find the areas of greatest relevance.

Drill Down: Throughout the platform, multiple drill down methods are provided to gain visibility into the data and help guide investigation. For example, clicking on a cell in the Gene Set Characterization similarity matrix pops up a panel that shows the number and percentage of genes in the user gene set that are also in that public gene set. In the example image, the user gene set, Ovarian Diseases, overlaps with 66.7% of the genes in the selected pathway gene set (Figure I in S1 File).

Data Handover between Pipelines

Multiple KnowEnG pipelines can be weaved together into workflows that offer a series of complementary perspectives on the user's data set. For example, a user could begin by partitioning a sample population into subgroups with Sample Clustering, proceed to find the genes most associated with the assigned cluster labels using Feature Prioritization, and conclude by identifying the most significantly enriched Gene Ontology terms with Gene Set Characterization. The KnowEnG platform allows the user to perform a variety of such multi-step analyses entirely inside the application, without having to download intermediate results and transform them for subsequent use. Fig 2A illustrates the supported types of pipeline handover.

To facilitate handover, spreadsheets produced in the execution of a pipeline are automatically saved alongside the user's uploads, grouped with the job that generated them. Later, when configuring another pipeline for execution, the user can simply select one of these output spreadsheets as an input.

Other KnowEnG User Experience Options

In addition to the web-based platform, KnowEnG provides two additional user experiences: Seven Bridges Cancer Genomics Cloud (SB-CGC) provides users with a query interface to work on major data sets, such as TCGA, alongside the KnowEnG analytical workflows. The Common Workflow Language is used to describe the sequence of container invocations that constitute the analysis. Such descriptions are then executed by the SB-CGC's engine, which provisions the necessary computational resources. For more see Appendix H in S1 File. Jupyter Notebooks are a popular tool for data analysis. Accordingly, KnowEnG offers a free, public web server pre-populated with a collection of notebooks that showcase the main analytical pipelines and enable common data manipulations. Users can step through the notebooks as provided, run new analyses by replacing the sample data with their own spreadsheets, and even introduce new functionality by editing the notebook scripts. The web server is built on the open-source JupyterHub project, deployed to AWS via Kubernetes, and offers users the same single-sign on technology as the platform. For more see Appendix E in S2 File. A list of very useful resources for these different KnowEnG User Experiences are provided in Table A in S1 Data.

Figures

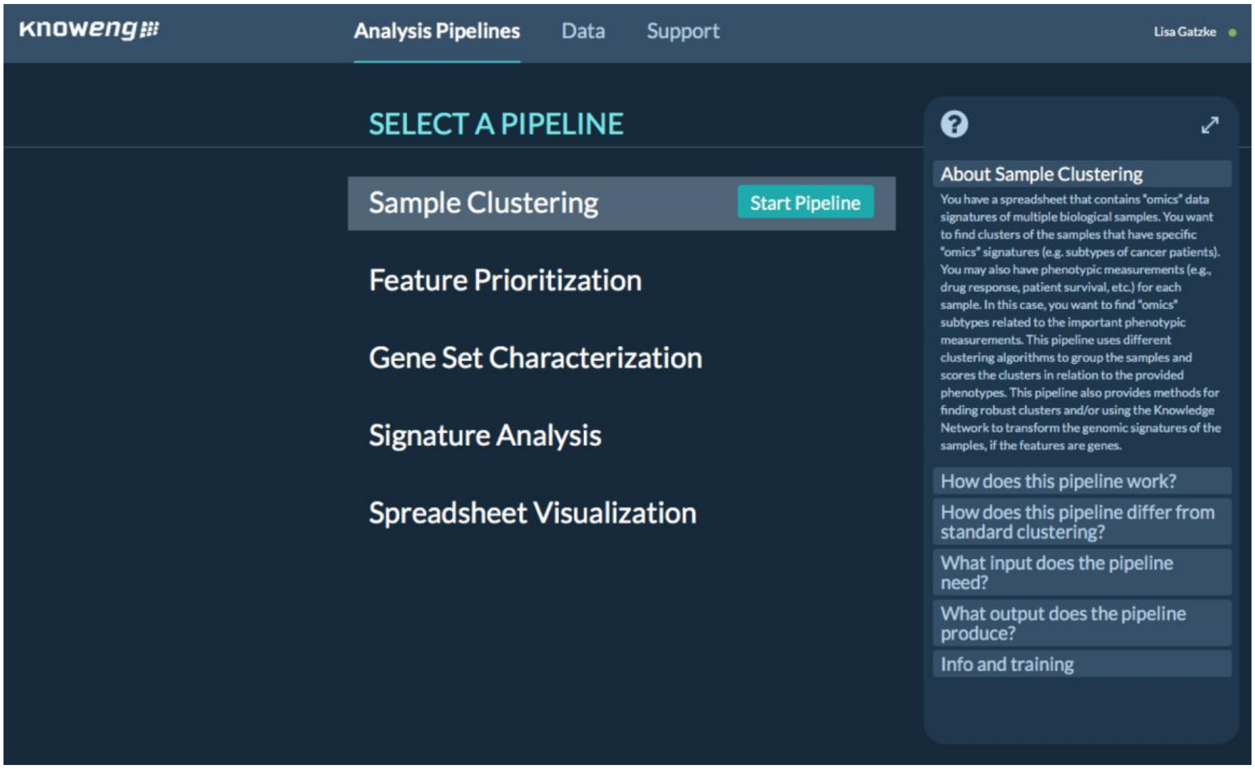

Figure B. Pipeline Home.
KnowEnG offers five pipelines that are commonly used in "omics" analyses. Frequently asked questions about each pipeline are addressed in the Info Panel on the right.

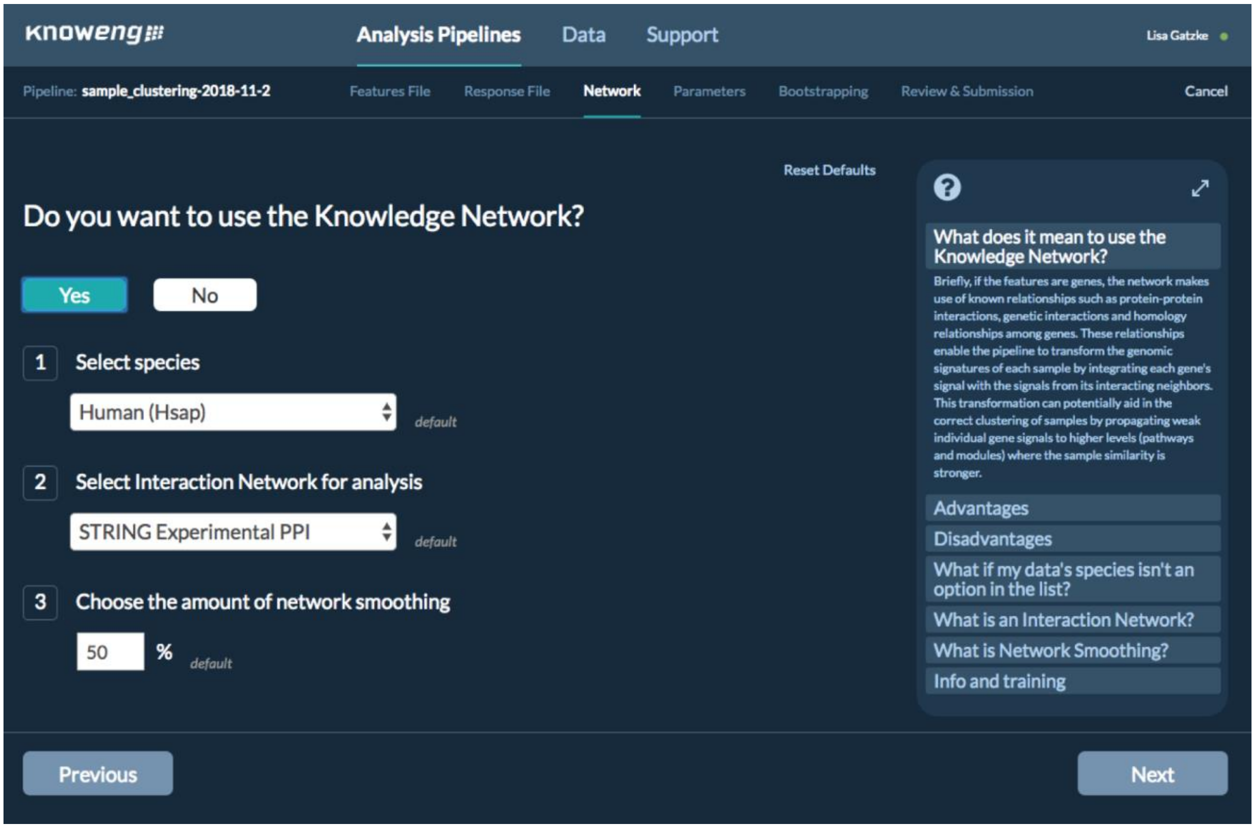

Figure C. Pipeline Setup Workflow.
Wizard-like interface that steps users through pipeline setup, including contextually relevant information provided in the Info Panel for each step. The above image shows the Network selections step in the Sample Clustering Pipeline.

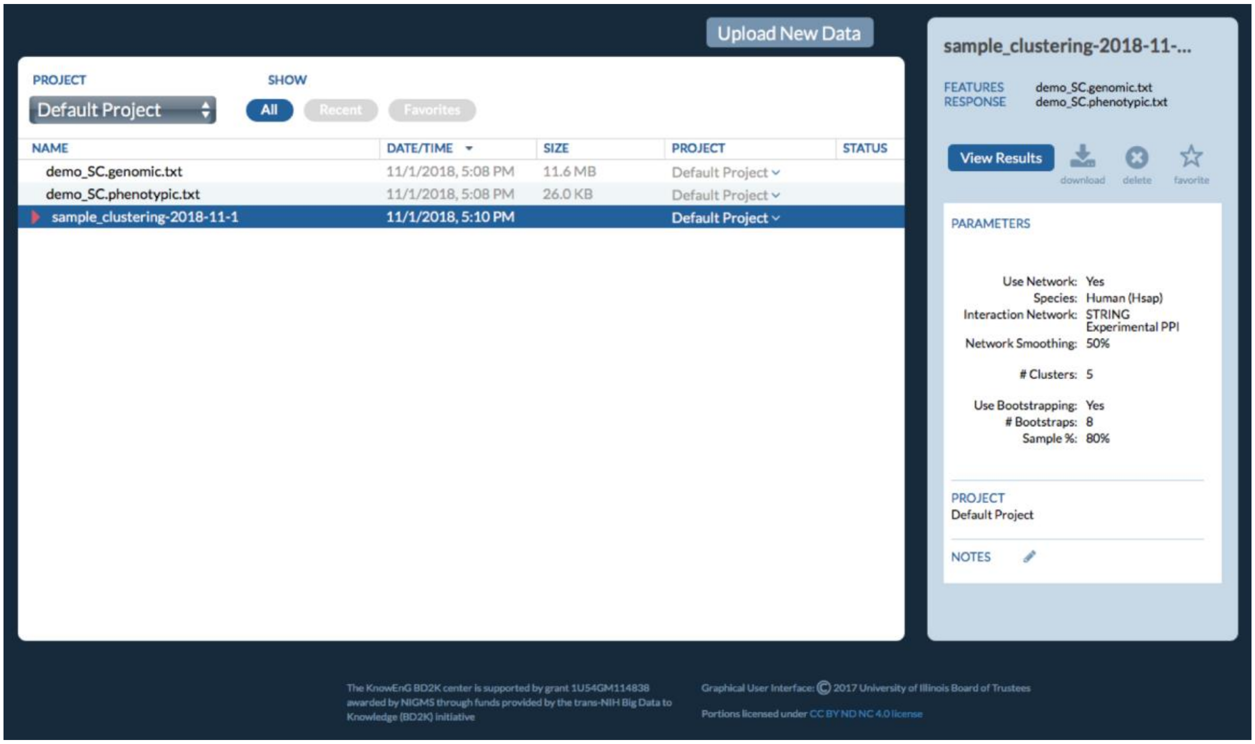

Figure D. The Data Table.

Appears when clicking on "Data" in the top navigation bar. The table provides persistent access to all pipeline run results as well as user uploaded data. Metadata about each file in the table is provided in the Info Panel on the right. Tools are also available to tag and add notes to data to facilitate data management.

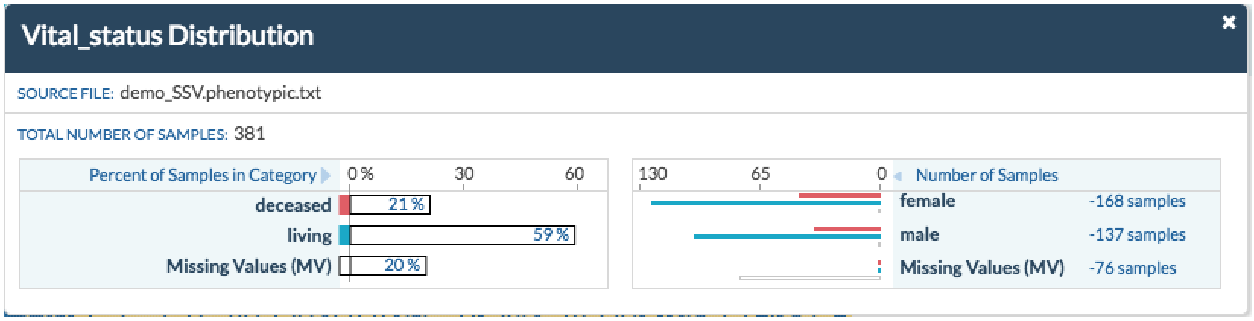

Figure E. Descriptive Statistics Panel.

Available by clicking on elements in the Spreadsheet Visualization interface (as seen in Fig 3E). Allows users to explore the distribution of data across all of the variables available.

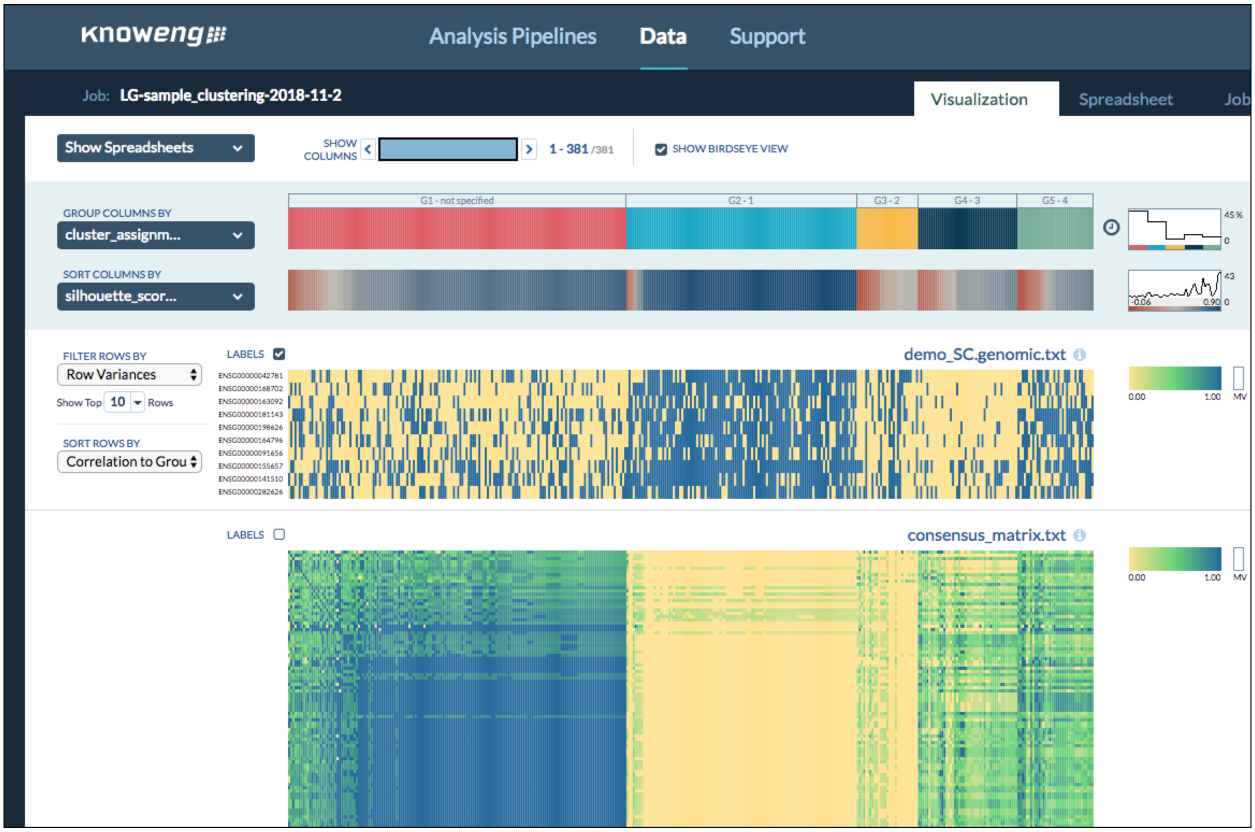

Figure F. Sample Clustering Visualization.

Offers several different heat maps that can be compared across columns for possible correlations among omics features and clinical features. The top two bars are “data-strips” that organize the columns by user-selected features.

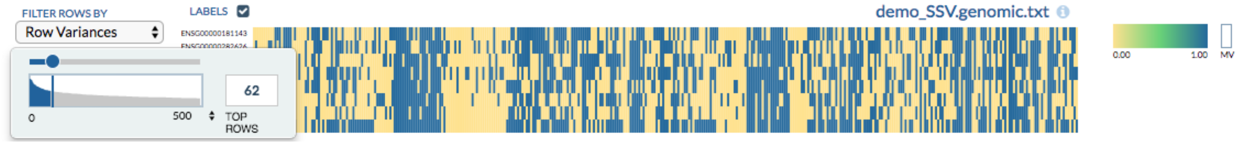

Figure G. Top Feature Selector.

Helps user make an informed decision about the number of omics features to display in the visualization. It is used in the Spreadsheet, Sample Clustering, and Gene Prioritization Visualizations.

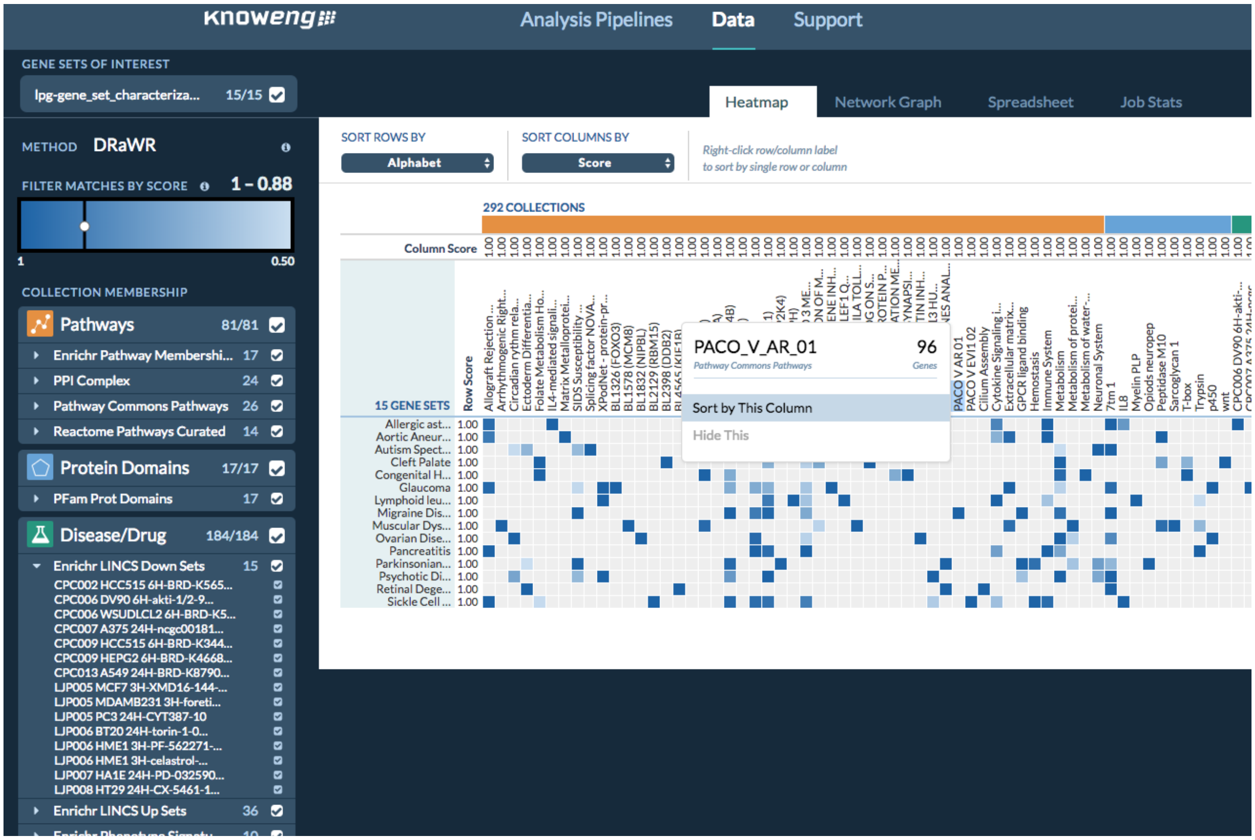

Figure H. Gene Set Characterization Visualization Tool.
Visualizes degree of overlap of user gene set(s) with public gene sets that are incorporated into the platform.

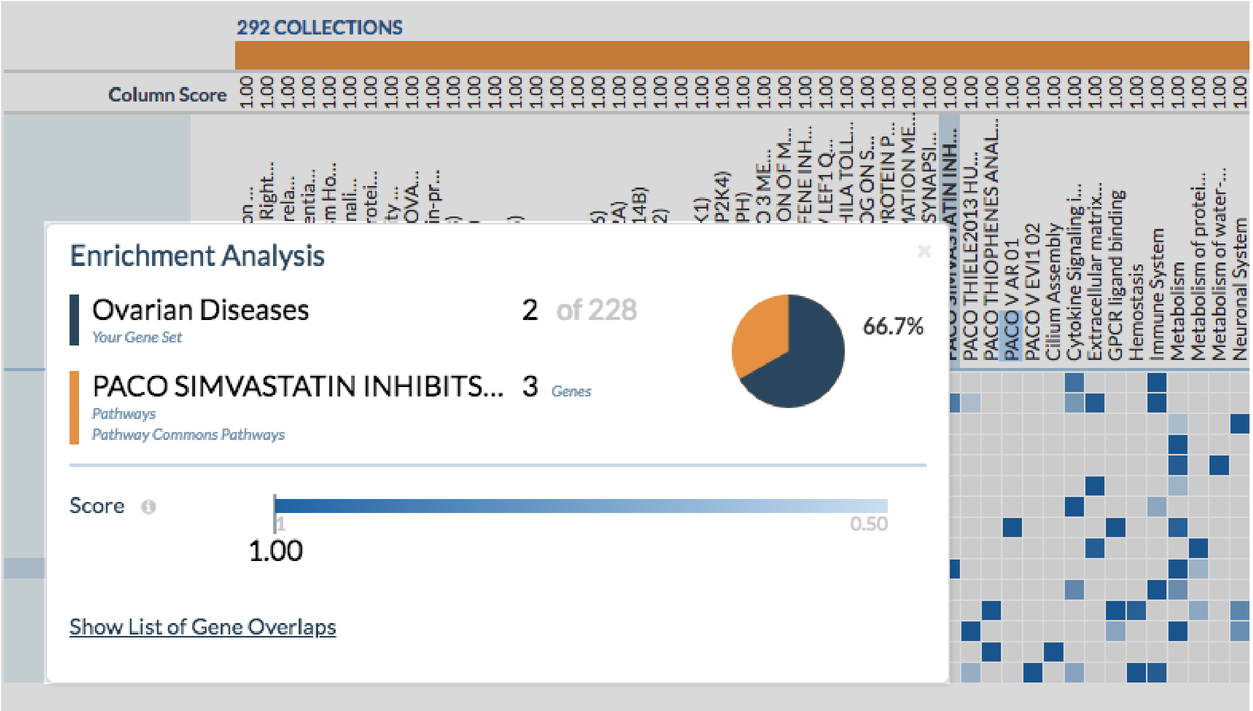

Figure I. Enrichment Analysis Drill Down Panel.
Calculates and visualizes the degree of overlap between the user gene set and the selected public gene set.

Tables

Table A. Resources for KnowEnG Pipelines.
Listed in each row are several important resources, their type, and url, publically available to users of the KnowEnG system.

Appendix C: Cloud Formation Template

Overview

AWS CloudFormation [https://aws.amazon.com/cloudformation/] offers tools to automate the deployment of pre-specified cloud infrastructures. The central concept of CloudFormation is the *template*, which is a description of the required AWS resources, any dependencies between them, and the runtime configuration parameters required to build a particular application stack. These templates are easily shared and version-controlled, allowing customized infrastructures to be precisely encapsulated as code to create replicate cloud instances in different regions and by different user accounts.

KnowEnG has published an AWS CloudFormation template that allows users to create their own instances of the KnowEnG analysis platform using their personal AWS accounts. This capability was developed for researchers whose data or analysis profiles were not conducive to the free use public KnowEnG instance. For example, researchers with sensitive data may want to create their own copy of the KnowEnG infrastructure with additional security protocols in place. Additionally, if a user wanted to run an analysis on a very large omics spreadsheet (>200 MB) or perform intensive computation with thousands of bootstrap iterations or exhaustive parameter sweeps (prohibited on the free use public instance), they could use the template to create a replicate of the KnowEnG platform with appropriately sized compute instances and without the restrictions of the public version.

The AWS CloudFormation template that creates new deployments of the KnowEnG platform, painlessly instantiating and integrating several components:
Container pods for platform services

- A Kubernetes [https://kubernetes.io/] cluster, consisting of several virtual machines:
    - One master node
    - Compute nodes whose number and size are specified by the user
- Virtual Private Cloud isolating the Kubernetes cluster from the public Internet
- Bastion virtual machine providing secure access to the Kubernetes cluster
- Elastic File System [https://aws.amazon.com/efs/] pre-loaded with the Knowledge Network, furnishing data to all pipeline containers and storing results from all analyses
- Resilient backend deployment of KnowEnG platform using containerized pods as a Kubernetes service
- Load-balancer exposing the web interface via a URL

Researchers who use the AWS CloudFormation template to deploy their own instance of the KnowEnG platform will bear the cost of the required infrastructure and of the resources necessary for any analysis runs performed on that instance. The major cloud-computing costs of running a personal instance can be decomposed into two parts:

1. **Virtual Machines**. This is the cost of running the virtual machines in the deployment. There is one t3.micro virtual machine that serves as the “bastion” for system administration, and there is one m5.large virtual machine that serves as the Kubernetes master. Together, these two virtual machines cost $0.1064 per hour. Additionally, the Kubernetes cluster includes compute nodes, the number and size of which can be adjusted by the user. For the default configuration of two m5.large virtual machines, the total compute-node cost is $0.192 per hour, bringing the total virtual machines cost of a default deployment to $0.2984 per hour. These prices are current as of May 2019, for the us-east-1 AWS region. The AWS EC2 price tables [https://aws.amazon.com/ec2/pricing/on-demand/] provide up-to-date information, including how changes to the number and type of the compute nodes and changes of region affect the cost of the virtual machines.
2. **Storage**. This is the cost of storing cluster data on the Elastic File System. As of May 2019, AWS charges $0.30 per gigabyte per month in the us-east-1 region. Users should expect at least 40 GB of storage, which would cost less than $0.018 per hour. The AWS

EFS price tables [https://aws.amazon.com/efs/pricing/] provide up-to-date information, including how changes to the amount of data stored and changes of region will affect the cost of storage.

Finally, we note that the existence of the CloudFormation template allows researchers to lower their costs by deleting their KnowEnG platform instance during periods of inactivity and redeploying it as needed.

Resources

KnowEnG CloudFormation Deployment GitHub repository [https://github.com/KnowEnG/Kubernetes_AWS/tree/master/cloudformation]
KnowEnG CloudFormation template: [https://s3.amazonaws.com/knowscripts/knoweng-platform-simple.template]

Appendix D: Knowledge Network Selection Guidance

Overview

The KnowEnG Analysis Platform offers users the opportunity to perform machine learning analysis on their omics datasets while making use of prior knowledge on gene interactions. This type of analysis can provide alternative, interesting results, but knowing which gene/protein interactions to include can be a daunting task. The knowledge-guided methods benefit when there are sufficient relationships between the gene entities of interest. In this supplementary appendix, we explore what types of interaction relationships provide the greatest benefit to a gene ranking task for many different types of gene sets.

The overall approach to this analysis relies on the gene ranking task performed by the DRaWR algorithm [3]. The DRaWR algorithm takes an input gene set and a heterogeneous network and attempts to rank all genes in the network for their relatedness to the input gene set. The network is formed with nodes that represent genes (and proteins) and edges that represent different types of relationships between those entities. The DRaWR algorithm uses Random Walks with Restart (see Appendix C in S2 File) in a manner that is similar to the KnowEnG Gene Set Characterization pipeline (see Appendix C in S4 File). The difference in this application is that we are using the method to rank the relatedness of the gene nodes to the input gene set and not ranking nodes that represent known annotations of functions or pathways.

For this analysis, we provide DRaWR a single gene-gene interaction network from the KnowEnG Knowledge Network (see Appendix A in S2 File) and a previously characterized set of genes. We divide the gene set into four equal sized partitions, and use 75% of the genes as the restart set in a RWR on the interaction network. We calculate the resulting gene set vector that shows a walker's long-term probability of being at any gene node in the network, and compare those values to a probability vector generated by a walker that restarts in the network randomly at any gene node. We rank genes for similarity to the gene set based on the difference between the two vectors, and find the ranking of the hidden 25% of the gene sets to

calculate the Area Under the Receiver Operating Curve (AUROC). We repeat this process for each partition, for each gene set, and for each gene-gene interaction network. We summarize these results below to access which network types are more amenable to this gene ranking task and how the type of input gene set affects ranking outcome.

Results

In this analysis, we collected 12 different gene-gene interaction networks for comparison (see Table B in S1 Data). Each of these networks we categorized into one of five different categories: networks that capture 1) transcriptional co-expression relationships, 2) DNA/protein sequence relationships, 3) physical interactions between proteins, 4) functional relationships within pathways, and 5) co-occurrence/integrated relationships from various literature resources. These 12 networks range in size from just over 100,000 to nearly seven million network edges/relationships in human databases. We also collected six additional interaction networks from the fruitfly to compare the results across species.

The other input to this analysis was nearly 48,298 human gene sets. These gene sets were taken from 30 gene set collections available through the KnowEnG Knowledge Network. The gene set collections were also grouped into six categories: gene sets derived from 1) experimental measurements in disease/drug perturbations, 2) ontologies of gene function, 3) annotations of biological pathways, 4) identification of protein structures, 5) disruptions and measurements of transcriptional regulation, and 6) assays on different tissues and conditions. Several of these gene set collections were divided into subcollections when gene sets came from distinct sources (Table C in S1 Data). The subcollections range from 12 to 5832 gene sets with average sizes ranging from 19 to 1772 genes that are in the gene set and the interaction network.

We ran the four fold cross validation mode of DRaWR as described above for all 12 single gene-gene interaction networks and all of the input gene sets. Network edges were treated as undirected and weighted and the restart probability was set to 0.5. The second stage of DRaWR was not run, and the AUROC for each run was calculated from the DRaWR ‘diff’ score.

The results show that in this gene ranking task, the Text_Mining/Integrated (average AUROC 0.664) and Pathway (average AUROC 0.624) based interaction networks generally performed better over all gene sets (panel A of Table D in S1 Data). The Experimental Interaction based network performed worst (average AUROC 0.583). This trend was also true in results produced from the six fruitfly networks using fruitfly gene sets from Gene Ontology, KEGG, Pfam, and Reactome (panel B in Table D in S1 Data). In Table E in S1 Data, we explore how these interaction network types perform on different gene set collection types. We find that curated Pathway, Protein Domain, and Ontology (average AUROC > 0.736) types of gene sets are easier to recover than other more experimental gene sets from Disease/Drug, Regulation, and Tissue_Expression (average AUROC < 0.610) (panel A in Table E in S1 Data). This pattern is reasonable considering that these strong performing types of gene sets can be embedded in the Text_Mining, Pathway_Database, and Conservation network interactions. We see that the CoExpression network type does generally poorer at identifying the missing genes from any

type of gene set collection. We also observe a major performance decrease for Experimental_Interaction networks on gene sets that are more experimental and less curated.

In Table F in S1 Data, we further divide the results to examine the effect of each single gene-gene network. One general trend shows that for the same category of interaction network, the more complete networks provide the greatest benefit. For example, STRING Function Database (391,679 edges) is the best Pathway_Database network and STRING Co-expression (1,400,944 edges) is the best Experimental_Interaction network. When considering the subcollections of gene sets, we can see that general trends do not always predict performance, but can provide valuable guidance (Table G in S1 Data). A Disease/Drug gene set collection like OMIM [4] gene sets is extremely well captured by Text_Mining/Integrated networks (AUROC 0.904) and very poorly captured by CoExpression networks (AUROC 0.581). On the other hand the MSigDB Cancer Gene Neighborhood gene sets [5] are captured by both network types equally well (AUROC ~0.72) and better than any other type. Co-expression networks (and Pathway networks) also seem to perform best for many different types of gene sets derived from the Gene Expression Omnibus [6], which perhaps represent the common case of experimentally defined differential expression gene sets.

Overall, when choosing an interaction network, if your gene set is defined by careful selection or annotated genes, it may be best to consider a Text_Mining/Integrated Network. In general, the more complete the network (in terms of number of edges) given its category, the more likely it will perform well in analysis tasks. Finally, co-expression networks seem to have a strong value specifically for differential expression gene sets derived from experimental assays.

Resources

DRaWR GitHub Repo [https://github.com/KnowEnG/DRaWR]
Knowledge Network Content Summary [https://knoweng.org/kn-data-references/]
Knowledge Network Downloadable Content Links [https://github.com/KnowEnG/KN_Fetcher/blob/master/Contents.md]

Tables

Table B. Gene-Gene Interaction Networks.

Listed in each row are the different gene-gene interaction network that were assessed in this analysis. Additional information about each network is reported including its category, identifier, and the number of network edges in the human and fruitfly.

Table C. Gene Set Collections.

Listed in each row are the different gene set collections that were assessed in this analysis. Additional information about each collection includes the collection category, identifier, and the aliases of possible subcollections. For each collection, we list the number of gene sets and the average size of those sets after mapping them to the gene-gene interaction network.

Table D. AUROC by Interaction Network.
The average AUROC across four folds for every gene set is listed for each interaction network as well as the number of folds averaged. The interaction networks are grouped and summarized by network category. Results are shown for A) human and B) fruitfly.

Table E. AUROC by Gene Set Collection Type and Network Type.
The average AUROC across four folds for every gene set is listed for each gene set collection type (rows) and interaction network type (columns) for the human only runs (A) and fruitfly runs (B).

Table F. AUROC by Interaction Network and Gene Set Collection Type.
The average AUROC across four folds for every gene set is listed for each gene set collection type (columns) and interaction network (rows) for the human only runs. The interaction networks are grouped and summarized by network category.

Table G. AUROC by Gene Set Collection and Network Type.
The average AUROC across four folds for every gene set is listed for each each gene set subcollection (rows) and interaction network type (columns) for the human only runs. The gene set subcollections are grouped by gene set collection type.

Appendix E: Knowledge Network Retrieval and Mapping

Overview

The KnowEnG Center has developed two valuable utilities in order to provide access and interoperability for users to our Knowledge Network of prior knowledge about genes and proteins. Both of these utilities, the KN_Fetcher and KN_Mapper, are developed in Python, and made available as auto-building Docker images on DockerHub. Both also contain a Common Workflow Language (CWL) description, making them executable on many standardized workflow runners and amenable for workflow visualization tools and user interfaces. These utilities with their GitHub, Docker, and CWL references are registered in the Dockstore developed by the Cancer Genome Collaboratory [https://dockstore.org/].

KN_Fetcher Utility

The first utility, the KN_Fetcher, is a tool that allows users to download a specific subnetwork of the Knowledge Network that relates to a single species and single edge type. In the version of the Knowledge Network used in this publication, there are 185 different subnetworks of this type. This tool can be helpful when examining the downloadable results from the KnowEnG platform, retrieving the networks used in a pipeline run for analysis and visualization. The utility is also valuable for users who wish to run the KnowEnG Analysis Pipelines outside the platform because it retrieves the network data already transformed into the appropriate format. Being able to run the KnowEnG prior knowledge-guided analysis pipelines in the SevenBridges Cancer Genomics Cloud is possible in part because the KN_Fetcher is able to pull the relevant

subsections of the Knowledge Network onto the SevenBridges cloud. The KN_Fetcher asks the user to supply a Knowledge Network version, species taxonomic identifier (Table A in S2 Data) and a Knowledge Network edge type [https://knoweng.org/kn-data-references/#kn_contents_by_gene-gene_edge_type]. It then uses Amazon Web Services (AWS) tools to retrieve three files that relate to that subnetwork from AWS Simple Storage Service (S3) [https://aws.amazon.com/s3/]. The returned files are the file listing the subnetwork edges, a file that contains mapping information for all of the subnetwork nodes, and finally a file that contains the metadata of the subnetwork including the provenance of the original external data files processed, information about the build command and date, and network statistics about the subnetwork. For users who are willing to manually download subnetwork files one at a time for the Knowledge Network build used in this publication, direct links can be accessed from our contents summary page [https://github.com/KnowEnG/KN_Fetcher/blob/master/Contents.md]

KN_Mapper Utility

The second utility, the KN_Mapper, is a tool that allows users to provide a list of gene names or identifiers, and retrieve the Ensembl stable identifiers that are used internally in the KnowEnG network-guided analysis pipelines. In the current version, this tool enables the mapping of over 48 million gene, transcript, and protein names and identifiers to stable identifiers for 583,933 genes across 20 species. The KN_Mapper tool is integrated into the Data Cleanup Pipeline and run before any knowledge-guided analysis in the KnowEnG Platform or on the SevenBridges Cancer Genomics Cloud. It can be used by any user who wishes to harmonize the entities of their dataset with the entities recorded in the KnowEnG Knowledge Networks. The inputs to this tool are a file that contains one original gene name on each line, as well as optional hints about the mapping, such as species taxonomic identifier. The tool then queries an in-memory Redis key-value database [https://redis.io/]. It outputs a file with the same number of lines where each line corresponds to the mapped Ensembl stable identifier, gene symbol, and full gene description. If no stable identifiers are found for the original query, the flag “unmapped-none” is returned. If multiple, different stable identifiers are found for the same query string, the flag “unmapped-many” is returned. The tool queries the submitted gene names from the current Knowledge Network Redis database, available at port 6379 at knowredis.knoweng.org. Instructions for setting up a local Redis database can be found at the GitHub readme [https://github.com/KnowEnG/KN_Mapper].

Network_Prepper Utility

One final utility that relates the KnowEnG Knowledge Network is the Network Prepper tool. This tool allows users to transform their own gene-gene knowledge networks from an original list of network edges to the format and internal stable identifiers necessary to be compatible with the KnowEnG knowledge-guided analysis pipelines. Essentially, the tool runs the KN_Mapper on the source and target nodes in the user-submitted network and then performs some basic network checks and operations. The input to the Network Prepper tool is a tab-separated edge file, where each row lists the source node identifier, target node identifier, and positive weight of an edge of the new gene-gene network. After mapping the first two columns of node identifiers, the tool will optionally make the network symmetric by inserting the corresponding directional edges. It will also remove any duplicate edges by only keeping the row that contains the largest

edge weight. Finally, the Network Prepper will output two raw map files that contain mapping information for each node and edge in the originally submitted network, as well as two final, “clean” files that show the network and the node mapping after all operations have been performed. The tool will return an error if less than 60% of the nodes or edges are successfully mapped by the KN_Mapper tool or if there are fewer edges than nodes in the final network. Metadata about the final network is also produced as an output. More information can be found at the GitHub readme [https://github.com/KnowEnG/Network_Prepper]

Resources

KnowEnG KN Tools Page [https://knoweng.org/kn-tools/]

Knowledge Network Fetcher Tool

GitHub Repository [https://github.com/KnowEnG/KN_Fetcher]
Docker Image [https://hub.docker.com/r/knoweng/kn_fetcher]
Dockstore Reference [https://dockstore.org/containers/quay.io/cblatti3/kn_fetcher]

Knowledge Network Mapper Tool

GitHub Repository [https://github.com/KnowEnG/KN_Mapper]
Docker Image [https://hub.docker.com/r/knoweng/kn_mapper]
Dockstore Reference [https://dockstore.org/containers/quay.io/cblatti3/kn_mapper]

Network Prepper Tool

GitHub Repository [https://github.com/KnowEnG/Network_Prepper]
Docker Image [https://hub.docker.com/r/knowengdev/network_prepper]

Appendix F: Reproducing Major Analyses in KnowEnG Platform

Overview

In this paper, we have demonstrated several bioinformatics analyses such as patient stratification, gene prioritization, gene set characterization and signature analysis on a few major data sets in cancer genomics (TCGA and METABRIC). In doing so, we have reproduced key results from the original studies [7-9] as well as gleaning new biological insights. In this supplementary appendix, we provide resources for users who wish to reproduce the majority of these analyses for themselves in the KnowEnG platform. In doing so, we highlighted both the sophisticated level of analysis possible and the ease-of-use with which multiple pipelines can be invoked, individually as well as in combination, to generate a multi-faceted narrative of the insights that the data have to offer.

We chose eight analyses to highlight that could be reproduced in the KnowEnG Analysis Platform (or the SevenBridges Cancer Genomics Cloud). The description of these eight analyses are found in Table H in S1 Data as well as the README of the associated GitHub

repository with the input data [https://github.com/KnowEnG/quickstart-demos/tree/master/publication_data/blatti_et_al_2019].

For all of selected reproducible analyses, we provide the exact input spreadsheets. The easiest way to access these spreadsheets is to import them into the saved data of your user account in the KnowEnG Platform web server. The data can be imported from the “Support” tab using the “Manuscript Data Access” page (https://platform.knoweng.org/static/#/support/manuscript). You simply need to select this publication from the drop down list and select the “Load Manuscript Data” button (see Figure J in S1 File). This will import all of the spreadsheet files using the naming convention described in the GitHub README and make them available as data files when configuring pipeline runs in the platform. Alternatively, files can be downloaded from a single zipped archive [https://s3.amazonaws.com/knoweng-publication-data/blatti_et_al_2019.tar.gz], individually from the spreadsheets directory in the repository [https://github.com/KnowEnG/quickstart-demos/blob/master/publication_data/blatti_et_al_2019/spreadsheets], or from the links in the repository's input data tables. Files downloaded this way can be loaded into the platform using the standard data upload methods.

In the README for each analysis, we provide the parameters of the pipeline that are needed to recreate the highlighted analysis in the paper. These parameters appear in the order and with the vocabulary found in the KnowEnG Analysis Platform. (The parameters names and requirements will likely differ in the SevenBridges Cancer Genomics Cloud depending on the pipeline). Once an analysis is submitted and finished in the platform, the pipeline visualization and several downloadable outputs will be produced. We have also highlighted the primary downloadable output of interest as part of the README. Often, this primary downloadable output is also the input to the next analysis example. Besides the eight exemplary analyses provided, most of the baseline and alternative analysis described in the paper can be recreated with these resources by modifying the Knowledge Network related parameters.

Resources

Instructions for Recreating Analyses [https://github.com/KnowEnG/quickstart-demos/tree/master/publication_data/blatti_et_al_2019]
KnowEnG Platform Data Import [https://platform.knoweng.org/static/#/support/manuscript]
Data for Recreating Analyses [https://s3.amazonaws.com/knoweng-publication-data/blatti_et_al_2019.tar.gz]

Figures

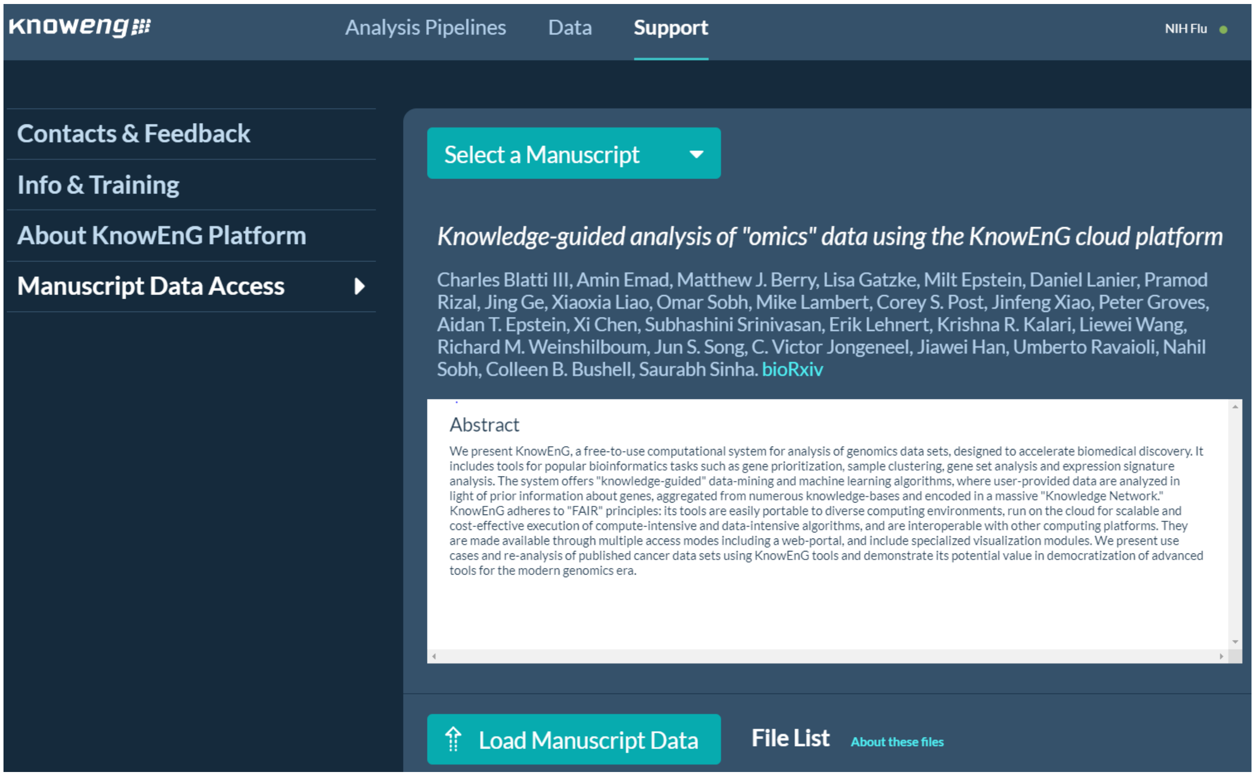

Figure J. Importing Spreadsheets for Reproducible Runs.
Shows the page in the KnowEnG Platform where the data that is necessary to reproduce the eight selected pipeline analysis runs can be automatically imported into the user's account and data collection.

Tables

Table H. Analyses for Reproduction in the KnowEnG Platform.
We highlight eight primary analyses that are found within the paper and that can be quickly reproduced in the KnowEnG platform. For each analyses, we list its related case study, analysis name, the pipeline it requires, the knowledge network that was used, the source of its input data, and the supplementary section where more details can be found.

Appendix G: Capabilities of the Spreadsheet Visualizer

Overview
Often, the first objective when beginning data analysis is to get a sense of what the data look like – *are there any striking imbalances between cohorts? any striking correlations with certain phenotypes? any missing data? any skewed data?* It is not easy to grasp this information

efficiently, and it is equally difficult for investigators to refresh their memories as they come back to prior studies. We developed a tool, Spreadsheet Visualizer, to address these challenges using descriptive statistics and interactive visuals (Figure K in S1 File).

The Spreadsheet Visualizer provides a flexible method for comparing multiple data types from the same patient samples, such as comparing between demographic and gene data, or other ‘omics data. We translate the values in these spreadsheets to colored “heatmaps” and provide many filtering and display options allowing investigators to check the data for errors and identify any obvious correlations between data types and phenotypes.

This tool is valuable at multiple stages of the data analysis process. As described, an initial understanding of the data ***prior to analysis*** can identify the need for further data cleaning and data transformation, and assist in developing hypotheses to be tested with computational analysis. The tool can also be helpful in studying the ***results of analysis*** by selecting spreadsheets derived from other pipelines such as Gene Prioritization. The tool is also helpful to convey ***reportable observations*** for publications and presentations.

The Spreadsheet Visualizer allows users to select multiple spreadsheets of related data that have been uploaded to the KnowEnG platform (see Appendix A in S1 File) for technical description. The pipeline executes standard variation and correlation statistics, distribution analysis and Kaplan-Meier time-to-event curves. An interactive visualization provides fluid exploration of the loaded data.

When using multiple spreadsheets, each spreadsheet must represent the same sample population. Spreadsheet orientation does not matter—that is, samples can correspond to rows or to columns—as long as the sample identifiers are consistent between files and all files have at least 50% of their samples in common. It is best to separate different data types into their own spreadsheets. For example, if a study includes gene expression data, somatic mutation data, and clinical data, the spreadsheet visualizer will be most effective if the data are organized as three spreadsheets, one containing the gene-expression data, one containing the somatic mutation data, and one containing the clinical data. When studying a single spreadsheet, it is best if the spreadsheet is oriented such that columns correspond to samples. Launching the pipeline is simple and requires the user to select the spreadsheets of interest (Figure L in S1 File).

User Interface Highlights

The tool provides both a qualitative overview of the data, as well as quantitative details, and supports the ability to fluidly shift focus and reorganize data, based on selected phenotypes and/or categorical features. This interface is rich with information design techniques including the following highlights:

Data Comparison

Central to this visualization is the ability to compare multiple spreadsheets for the same study samples. For example, one of the heatmaps can display expression values of top genes, and another can show DNA methylation. All heatmaps align so that the columns across all visuals

represent the same sample (e.g. patient). The biologist can choose a specific phenotype from their data, such as “Cancer Stage”, that is displayed as a single row heatmap at the top of the view. This “Group By” phenotype defines the grouping of all the samples (columns) in all the heatmaps. A secondary phenotype can be selected for sub-sorting the columns within each group. Additional single row heatmaps, or *“data-strips”,* can be displayed for a single variable and appear below the heatmaps in the same alignment for easy comparison of data. These variables are selected from any of the loaded spreadsheets and can be either categorical data such as “survival status”, or continuous variables such as “blood pressure” (Figure M in S1 File). The score beside each of these single-row heatmaps reflects the strength of the statistical association between the row and the “Group By” category that has been selected at the top of the visualization. For rows of continuous data, we apply an ANOVA test, and for rows of categorical data, we apply a Chi-squared test. In both cases, the displayed score is the negative log10(pvalue), capped at 200.

The rows of the multi-row heatmaps represent the data values for each sample. For example, the rows may represent genes and their expression value per sample. These displayed rows can be sorted by row variance or by significance of correlation to the displayed *Group by* phenotype. This correlation score is determined by an ANOVA test. The quantity of rows displayed can be determined by these same measures. The biologist can choose to display the top *N* rows, or view a graph depicting the score for each variable to better determine a meaningful threshold for display (Figure N in S1 File).

Data Distribution

Understanding how the data are distributed, and the relationships between distributions based on various phenotypes, provides a richer understanding of the data. Small graphs are included adjacent to each *data-strip* (Figure O in S1 File). providing a quick reference to the distribution of values for that variable. Quantitative details are provided by rolling over segments of the graph (Figure P in S1 File). These small yet powerful graphs facilitate visual skimming of the data – a task that is not often addressed by other tools. Clicking on the *data-strips* displays statistical details of the distribution, including the distribution based on the *Grouping Phenotype* displayed at the very top. This is recomputed each time the user re-sorts using other phenotypes of interest for grouping (Figure Q in S1 File).

Heatmaps

Heatmaps for large spreadsheets of data provide a way to comprehend the numerical values across many variables and samples. The visualization allows users to explore the data at multiple resolutions: (1) all samples at once in a “*Birdseye View”,* (2) only the samples in a selected group, or (3) “pages” of 250 samples at a time. Whenever the number of displayed samples exceeds the number of horizontal pixels available, cell colors will be blended to provide a sense of the data values on a single screen; i.e., without horizontal scrolling. The researcher can then toggle between the zoomed-out view and a zoomed-in view using the navigator (Figure R in S1 File). Interactive rollovers for each cell of the heatmap help researchers find specific detail about the data, such as sample ID, gene name and expression value (Figure S in S1 File). Color map choices are provided so the user can choose a color system that works best with their data (Figure T in S1 File).

Time-to-Event Curves

For spreadsheets that contain time-to-event measures, such as years of survival, the researcher can generate a Kaplan-Meier analysis for any categorical phenotype of interest. To generate curves, the researcher clicks on the clock icon adjacent to that single-row heatmap. This opens a popup where the time variable and event variable are selected and the graph is created (Figure O in S1 File). For example, a graph is easily computed to compare survival between “Anatomic Neoplasm Subdivision” groupings. The graph includes a log-rank-p-value that is a summary statistic capturing the extent to which the differences between the groups’ curves are significant (Figure U in S1 File).

Figures

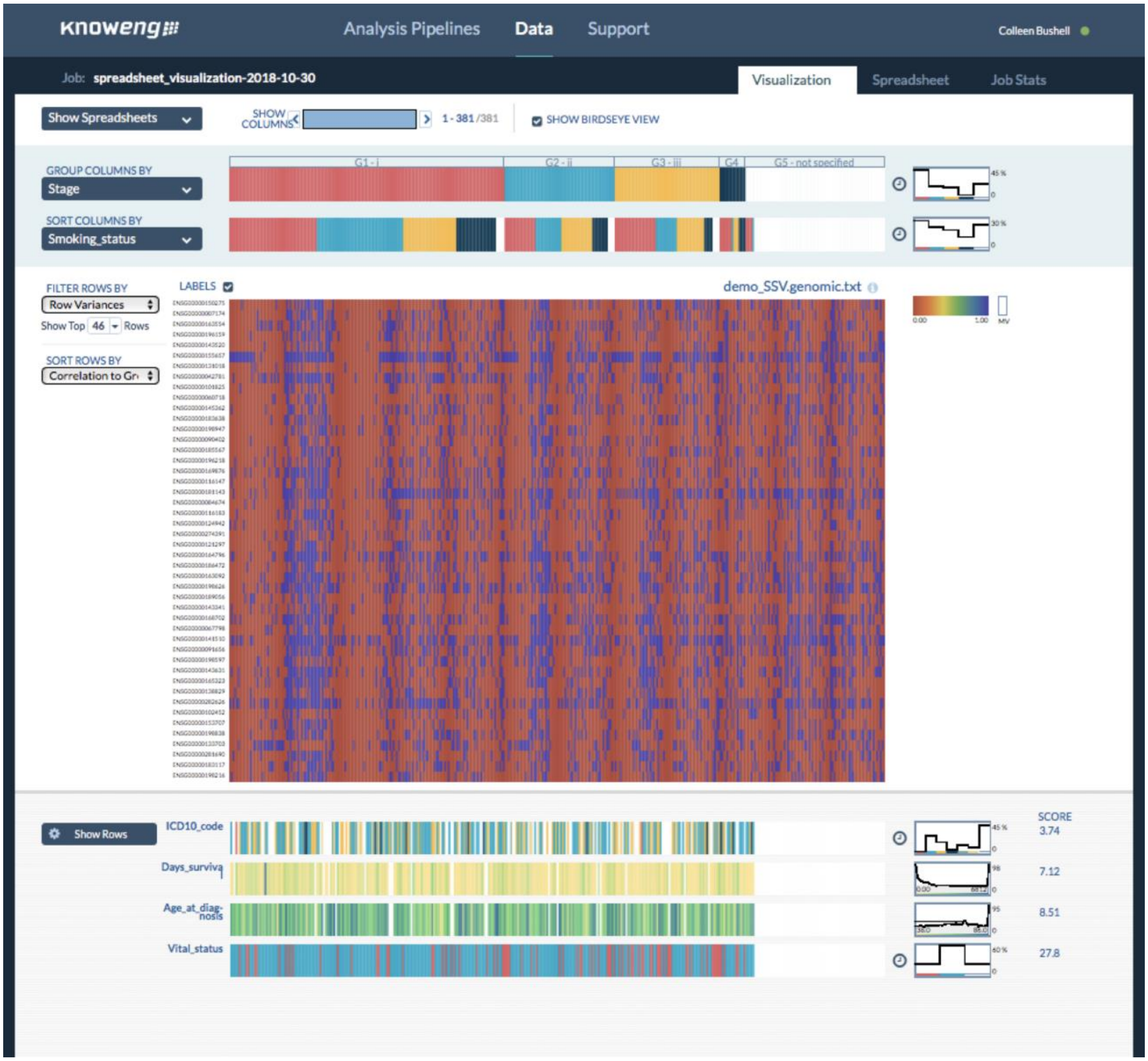

Figure K. Spreadsheet Visualizer Interface.

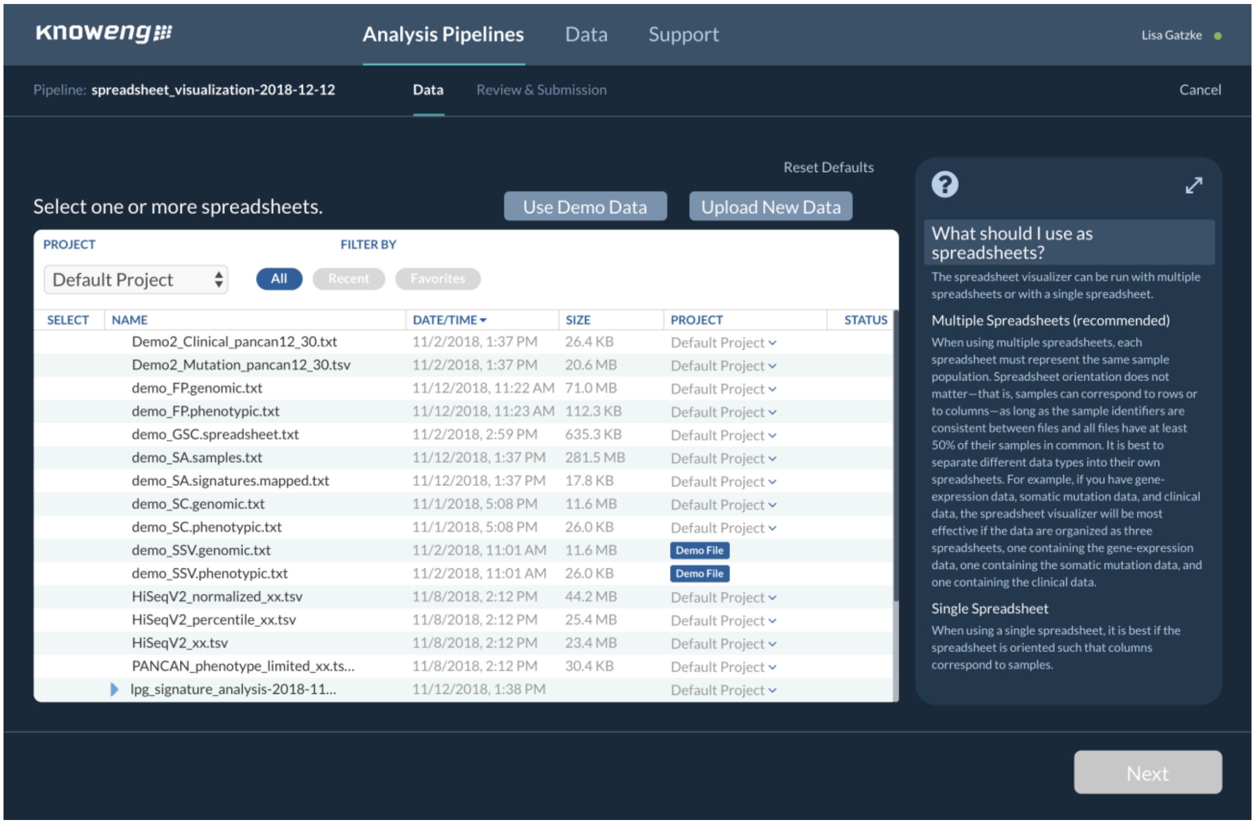

Figure L. Spreadsheet Visualizer Data Upload.
Used for selection of spreadsheets for visualization.

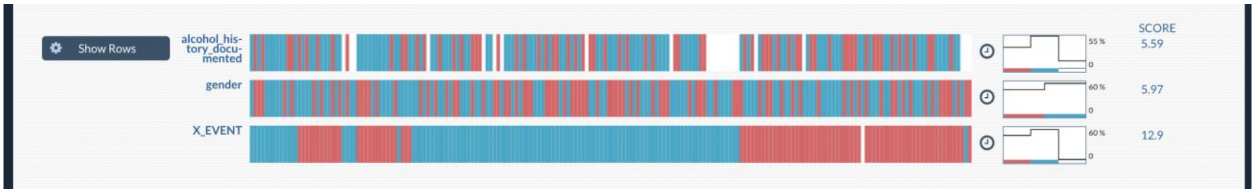

Figure M. Spreadsheet Visualizer "Data-Strips".
Use these to visually scan for correlations across the different spreadsheets.

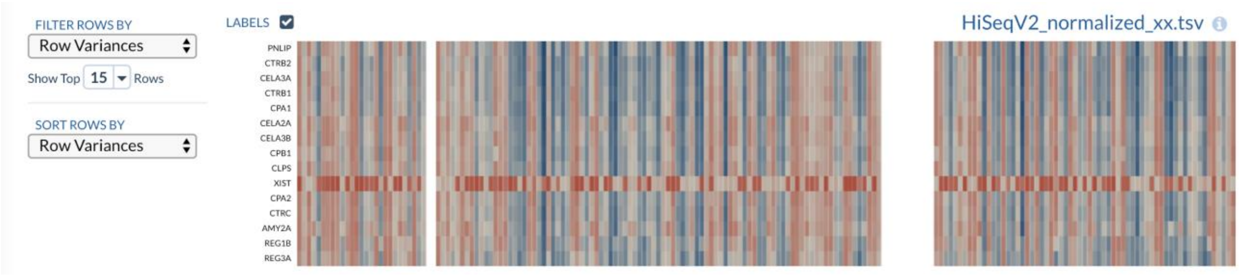

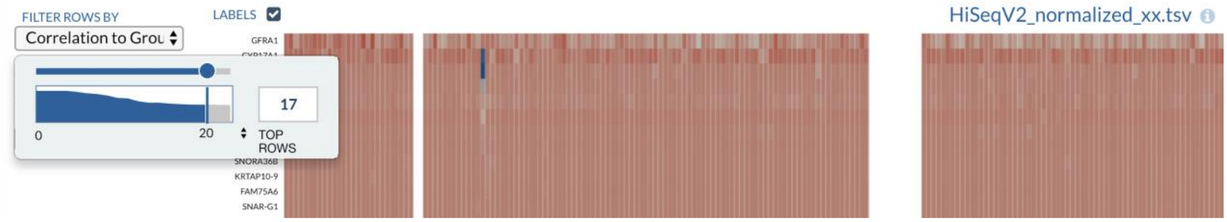

Figure N. Row Filtering and Sorting Controls.
Rows can be filtered and sorted by Row Variance score as well as Correlation to the Group By category selected at the top.

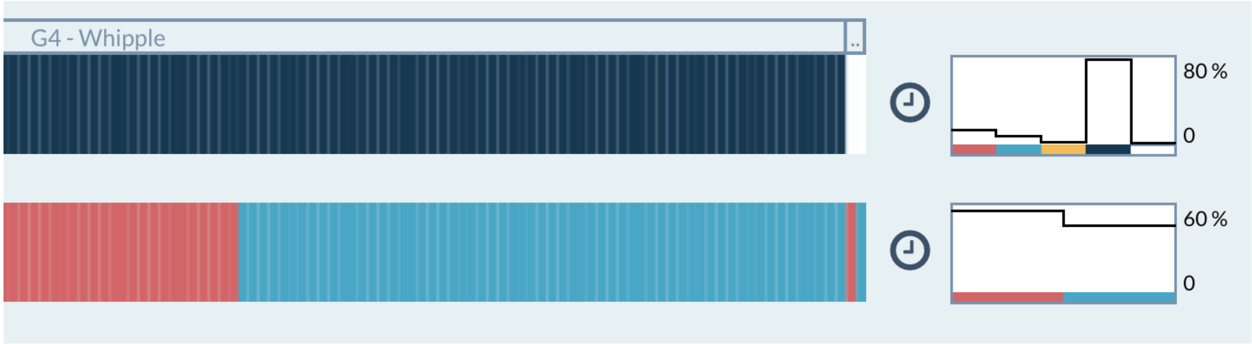

Figure O. Spreadsheet Visualizer Distribution Graphs.
Spreadsheet Visualizer “data strips” distribution graphs shown on the right.

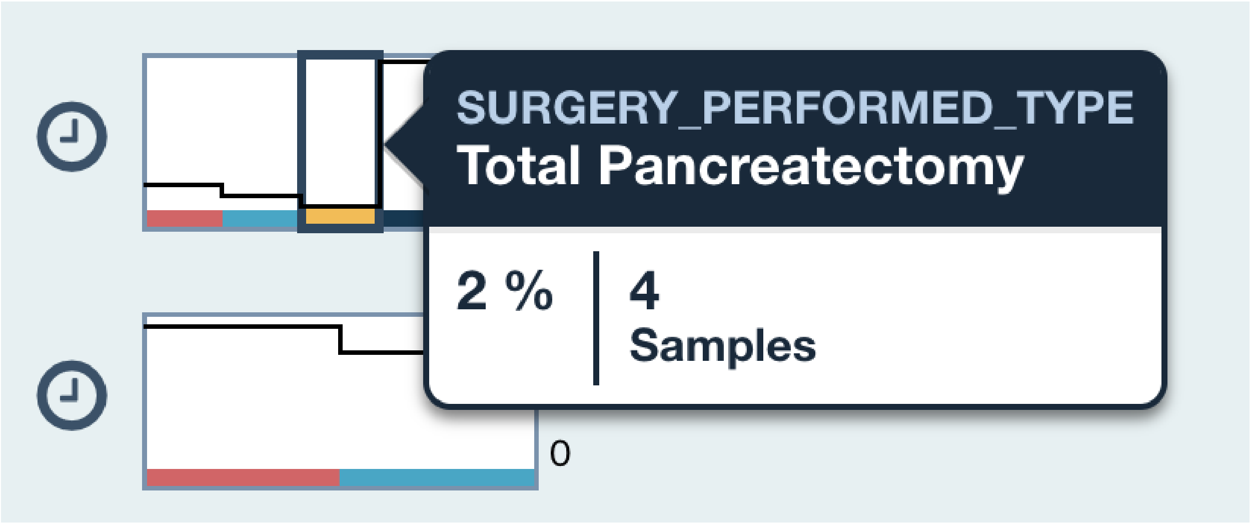

Figure P. Distribution Graph Hover Information.
Spreadsheet Visualizer “data strips” distribution graphs provide additional statistical information on hover.

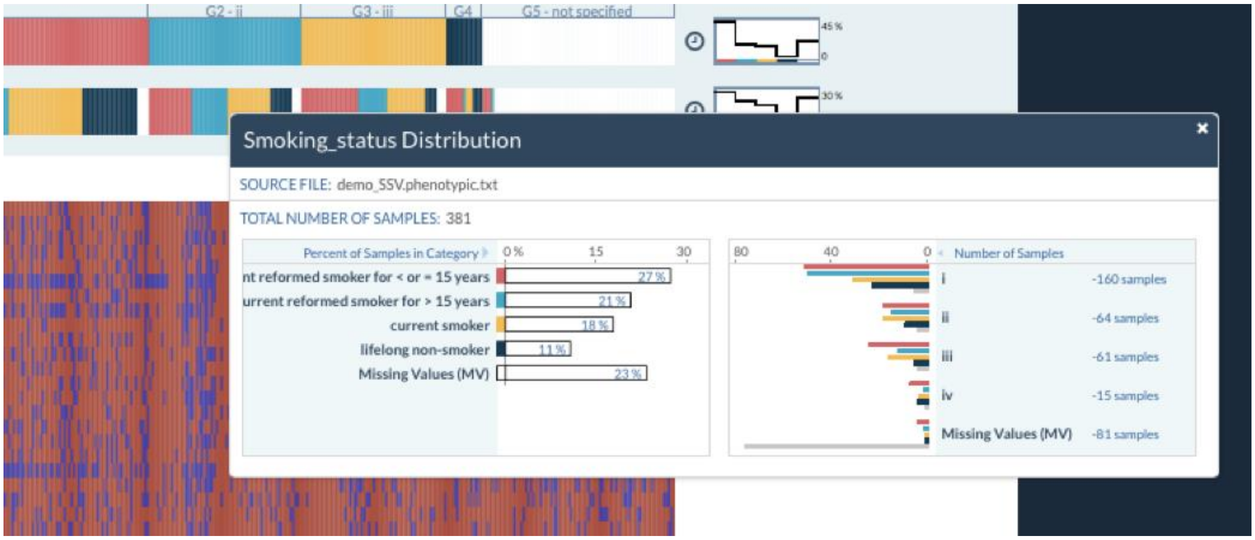

Figure Q. By Group Data Distribution.

Clicking on Spreadsheet Visualizer “Group by” and “Sort by” data strips triggers a popup showing the distribution of the data across the category bins as well as showing how these distribute across the “Group By” category selected at the top.

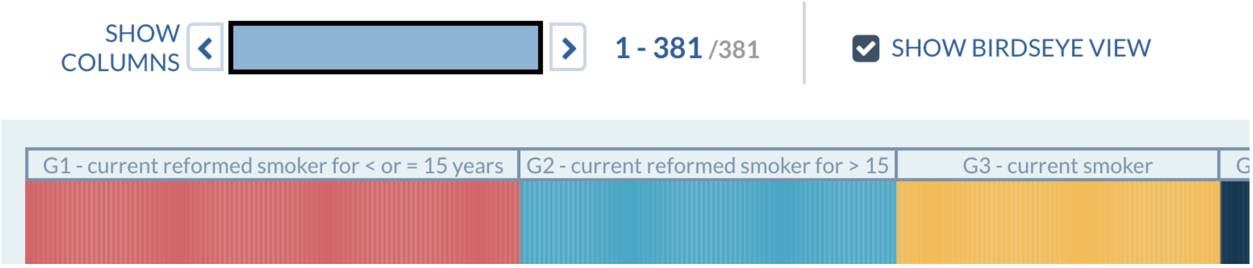

Figure R. Birdseye View.

The Navigator controls help users control how many columns are in the display at a time. When there are many more columns than can be displayed on screen, “Show Birdseye View” can be selected. Cell colors will be blended to provide a sense of the data values on a single screen the user a sense of how all of the data looks without horizontal scrolling.

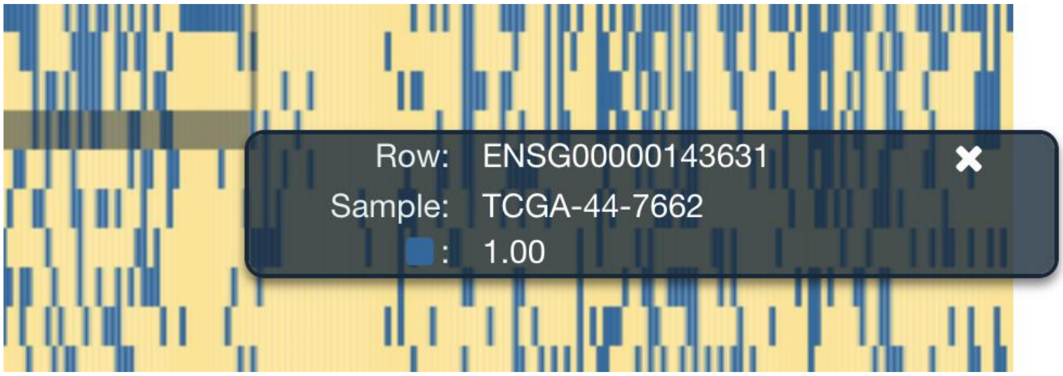

Figure S. Data Cell Hover Over.
The Navigator controls help users control how many columns are in the display at a time. When there are many more columns than can be displayed on screen, “Show Birdseye View” can be selected. Cell colors will be blended to provide a sense of the data values on a single screen the user a sense of how all of the data looks without horizontal scrolling.

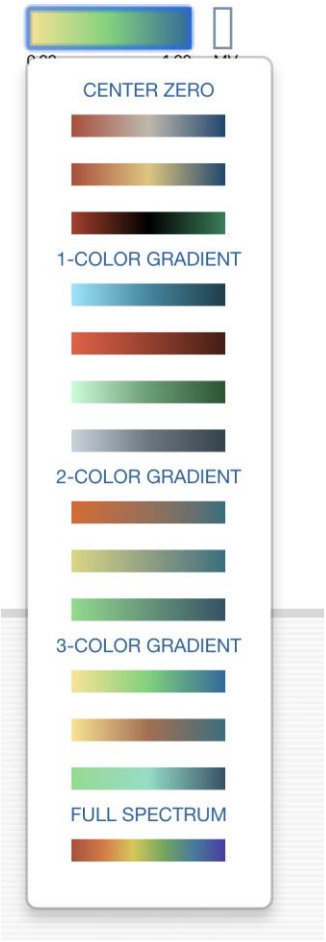

Figure T. Color Scale Map.
A variety of color map choices are available and can be switched out on the fly depending on the needs of the data being displayed.

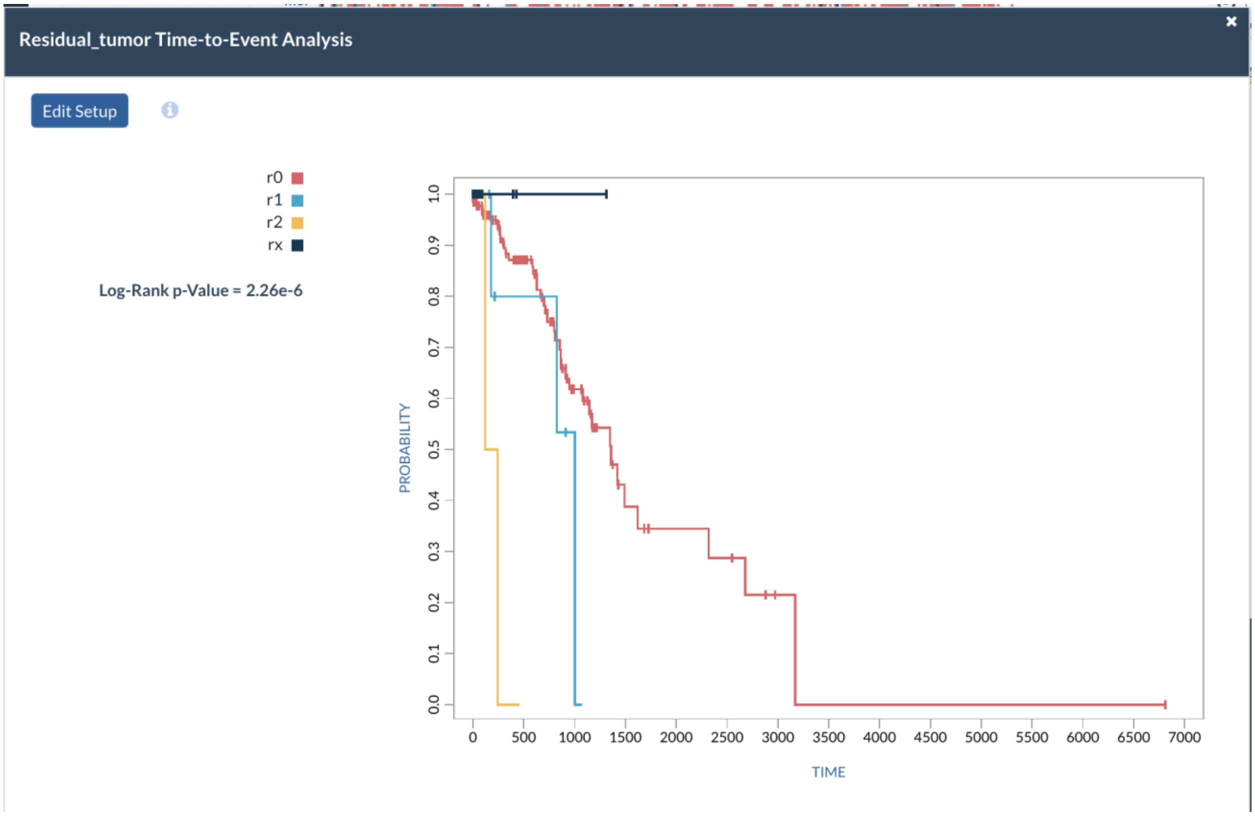

Figure U. Survival Curve.
For spreadsheets that contain time-to-event measures, such as years of survival, the researcher can generate a Kaplan-Meier analysis for any categorical phenotype of interest.

Appendix H: KnowEnG Analysis on SB-CGC using Docker and CWL

Overview

Seven Bridges Cancer Genomics Cloud (SB-CGC)

The Cancer Genomics Cloud (CGC) [https://cgc.sbgenomics.com/], built by Seven Bridges Genomics as part of the National Cancer Institute's Cancer Genomics Cloud pilot program, is a cloud-based environment that hosts large genomic data sets (e.g., TCGA) as well as genomic analysis applications. These apps can be executed on the CGC, which uses Amazon's AWS behind the scenes.

Apps are either **command line tools** or **workflows** built up from other apps. They can be developed on, or imported to, the CGC site. KnowEnG [https://knoweng.org/] has created apps on the CGC for several of the pipelines we have developed.

Figure V in S1 File shows one tool imported from KnowEnG, Gene Set Characterization (GSC), being edited directly on the CGC Tool Editor web interface. The execution of this pipeline tool is specified with an appropriate command line call and the associated GSC Docker image developed by KnowEnG. Besides the Tool Editor interface, CGC also provides the Rabix Composer IDE that allows editing and testing CGC apps on one's own local machine.

In general, for the KnowEnG pipelines, both a command line tool and a workflow version are necessary. The latter involves collecting all the inputs for the pipeline and modifying and distributing them as necessary to the different components of the workflow, running all the components of the workflow, and collecting and modifying the outputs.

Figures W and X in S1 File show the workflow version of the GSC pipeline. Figure W in S1 File shows the app's description, and Figure X in S1 File shows creating/editing the app on the CGC. For a workflow, this mostly involves dragging and dropping icons representing apps (command line tools or workflows), connecting them as needed, and configuring the inputs and outputs of the overall workflow and the internal components.

The CGC also provides some public analysis tools of their own, and allows apps developed on their site to be published and made available to others. KnowEnG has published several of their analysis pipelines on the CGC. Figure Y in S1 File shows the KnowEnG apps on the CGC.

Technologies for Importing Workflows

Two of the main technologies that allow for tools to be developed on, imported to, and run on the CGC are Docker and the Common Workflow Language (CWL).

Docker [https://www.docker.com/] provides containerization, a form of virtualization that allows for capturing all of the system, environment, library, and code dependencies an application needs in a standard, lightweight object. Docker containers can be executed in any computation environment.

When a command line tool is developed on the CGC, a Docker container is specified. This can be seen in Figure V in S1 File, where the container is specified in the field **Docker Repository[:Tag]**. This container is used when the tool is executed.

CWL [https://www.commonwl.org/] is a language for describing analysis tools and workflows. It specifies the details of the inputs, outputs, and execution steps of the tools. This allows for making the tools portable and scalable across of variety of software and hardware environments. There are many execution engines for CWL, taking a tool's CWL description and executing it.

Normally, apps on the CGC are developed interactively and graphically, and the CWL is created behind the scenes. But it is also possible to import an app already described in CWL. Similarly, it is possible to export an app from the CGC into a pure text CWL format. Figure Z in S1 File shows a portion of the CWL description of the GSC command line tool app.

Methods

Run Combined, Multi-pipeline Workflow

As stated earlier, KnowEnG has made available several of our pipelines as workflows on the CGC. These pipelines are useful by themselves, but additionally they can be used in conjunction to create more complex workflows.

To achieve this, one possibility is to run each workflow individually, using outputs from some workflow runs as inputs to others. With just a little bit of work on the CGC, however, it is possible to relatively quickly create such larger workflows as apps themselves from the individual lower level workflows.

Figure AA in S1 File shows an example of this, where the KnowEnG Analysis of LUSC Subtypes (KALS) workflow is built up from several of the other KnowEnG workflows.

Figure AB in S1 File shows editing the KALS workflow, with the node for the GSC workflow highlighted. When integrating an app into a workflow, one can choose whether to hardcode values for that app's input parameters, or to "port" them and make them available as inputs in the new, larger workflow. Similarly, one can choose which of the app's outputs to make available.

This document [https://github.com/KnowEnG/KnowEnG_CWL/tree/master/CGC] describes how to run the workflows on the CGC, both individually and the combined workflow directly.

Sharing and Reproducibility

In publishing these tools on the CGC, KnowEnG is working towards the "FAIR" principles: Findable, Accessible, Interoperable, and Reusable. They are findable through the CGC, as well as Docker Hub and GitHub. The versions on the CGC are accessible to researchers, ready to use with the public data sets available there or with their own data. They are interoperable with other tools and workflows available on the CGC and they are reusable in a reproducible manner with the other CGC datasets and collections in the KnowEnG Knowledge Network.

Users are able to run KnowEnG tools singly or in combination. As an example, we published a multi-tool workflow, the aforementioned KALS workflow that performs knowledge-guided enrichment analysis on sets of genes that are differentially expressed between TCGA samples that map to different genomic subtypes.

One could easily envision bringing additional custom collections of knowledge bases into this analysis framework using the Docker and CWL paradigm demonstrated here. By seamlessly linking petabyte-scale data access to the full suite of bioinformatics tools and advanced analytics available on the CGC and KnowEnG platforms, an even more valuable data analysis ecosystem is made available to researchers worldwide.

Resources

KnowEnG KnowEnG_CWL GitHub repo
[https://github.com/KnowEnG/KnowEnG_CWL/tree/master/CGC]
KnowEnG Public Apps on the CGC
[https://cgc.sbgenomics.com/public/apps#q?search=knoweng]

Figures

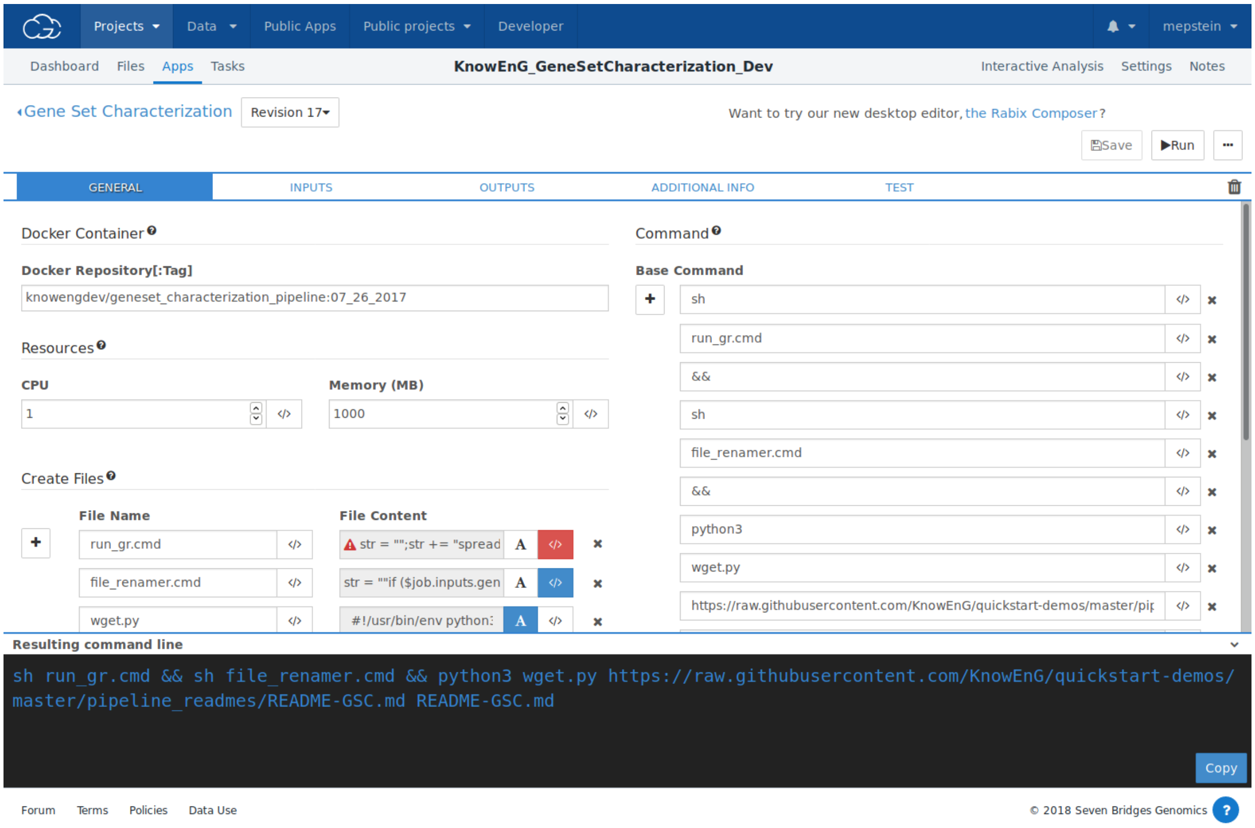

Figure V. Editing Tool on the SB-CGC.
This page shows the editing of the Gene Set Characterization command line tool on the SB-CGC. There are several tabs available to edit different aspects of a tool; this figure shows the **GENERAL** tab, which includes things like the command being run, its arguments, the docker container, the compute resources required, and associated files; other tabs include **INPUTS** and **OUTPUTS**.

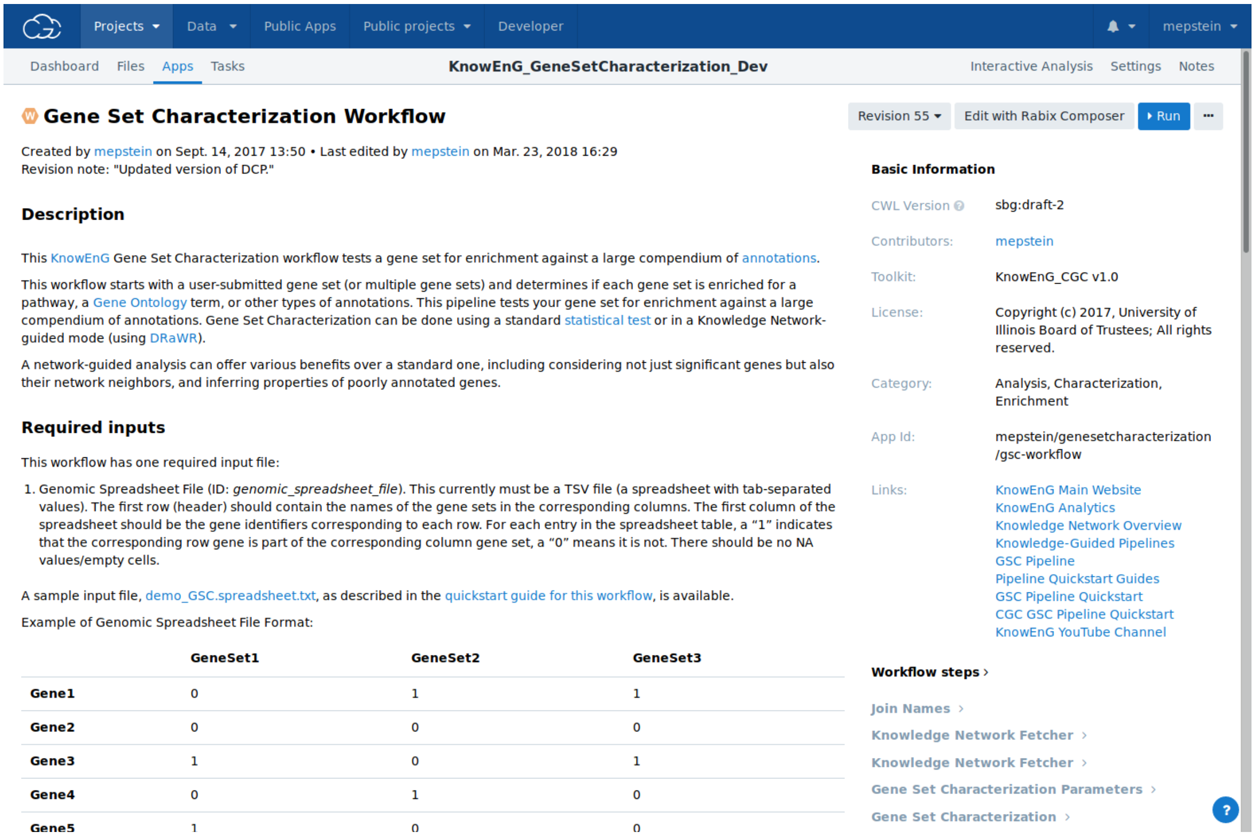

Figure W. The GSC Workflow Description.

This page can be seen at [https://cgc.sbgenomics.com/public/apps#mepstein/knoweng-genesetcharacterization-public/gene-set-characterization/]. The Description is the documentation for an app; conventionally, it includes a variety of information about the app, including a general description of the app, how it is used, and its inputs and outputs. It also includes a specification of a sample command line (for command line tools) or an image of the workflow.

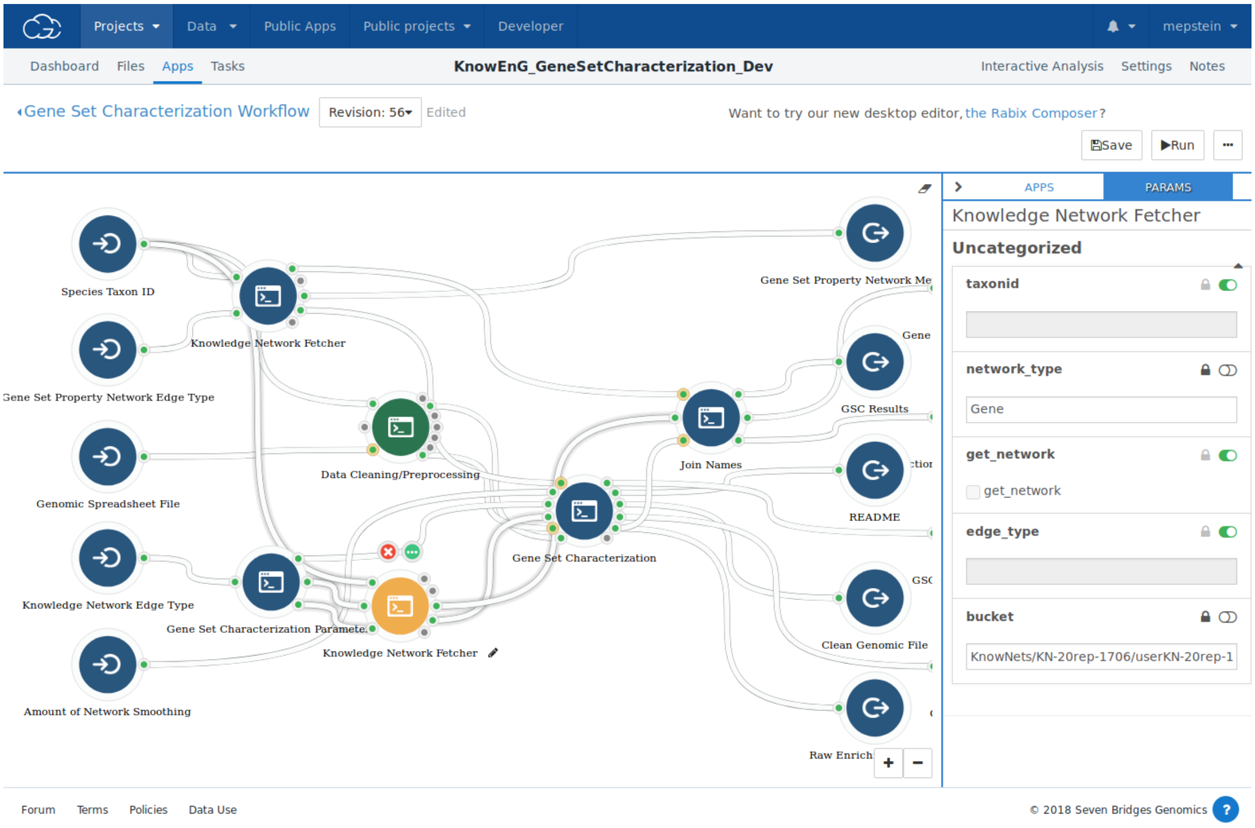

Figure X. Editing the GSC Workflow.

Editing a workflow on the SB-CGC primarily consists of creating a graphical image of the workflow (using drag-and-drop); the nodes to the left are the inputs to the workflow, those to the right are the outputs, and those in the middle are the component apps of the workflow. In this figure, where one of the apps is selected, the fields to the right show the inputs for that app; hard-coded values can be set there, or inputs can be ported and made inputs for the overall workflow.

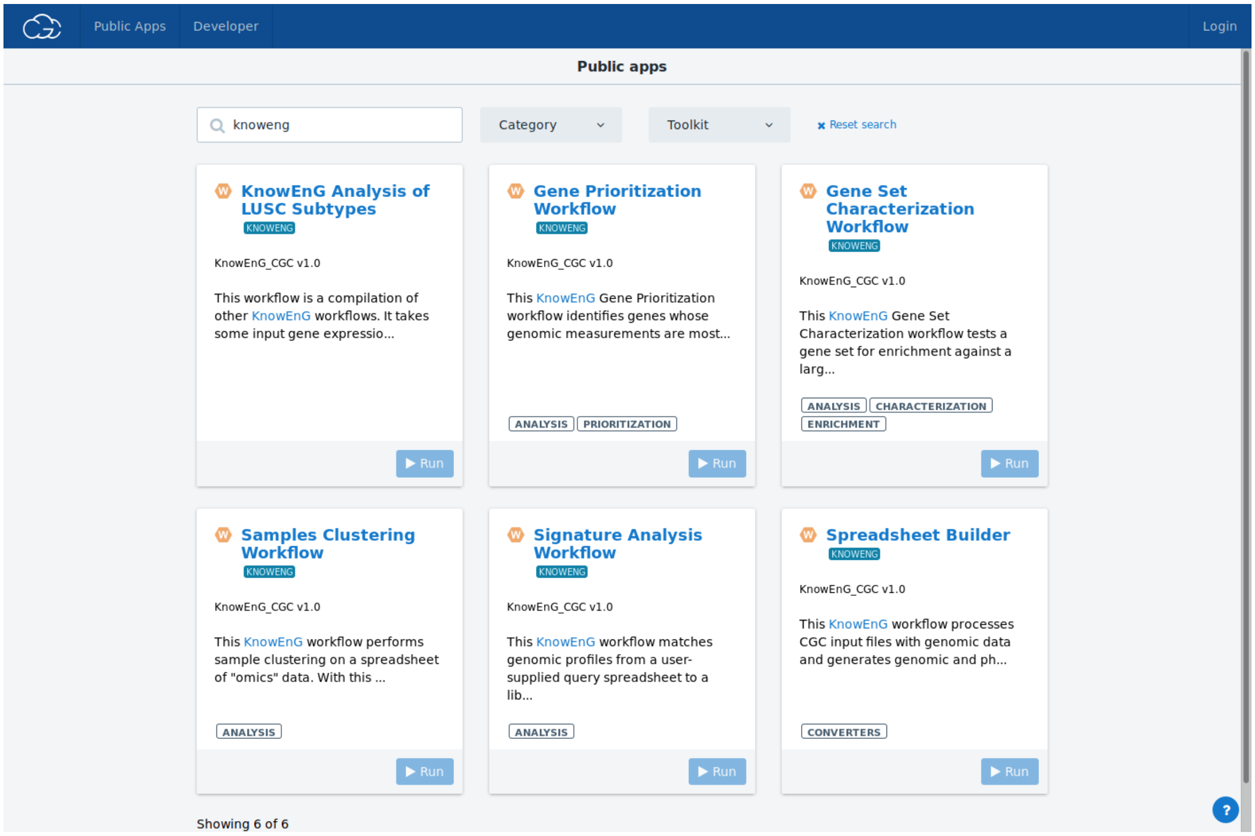

Figure Y. KnowEnG Public Apps on the SB-CGC.
This page can be seen at [https://cgc.sbgenomics.com/public/apps#q?search=knoweng]. It is easy to search through the many public apps at the SB-CGC. Currently, KnowEnG has published six apps there.

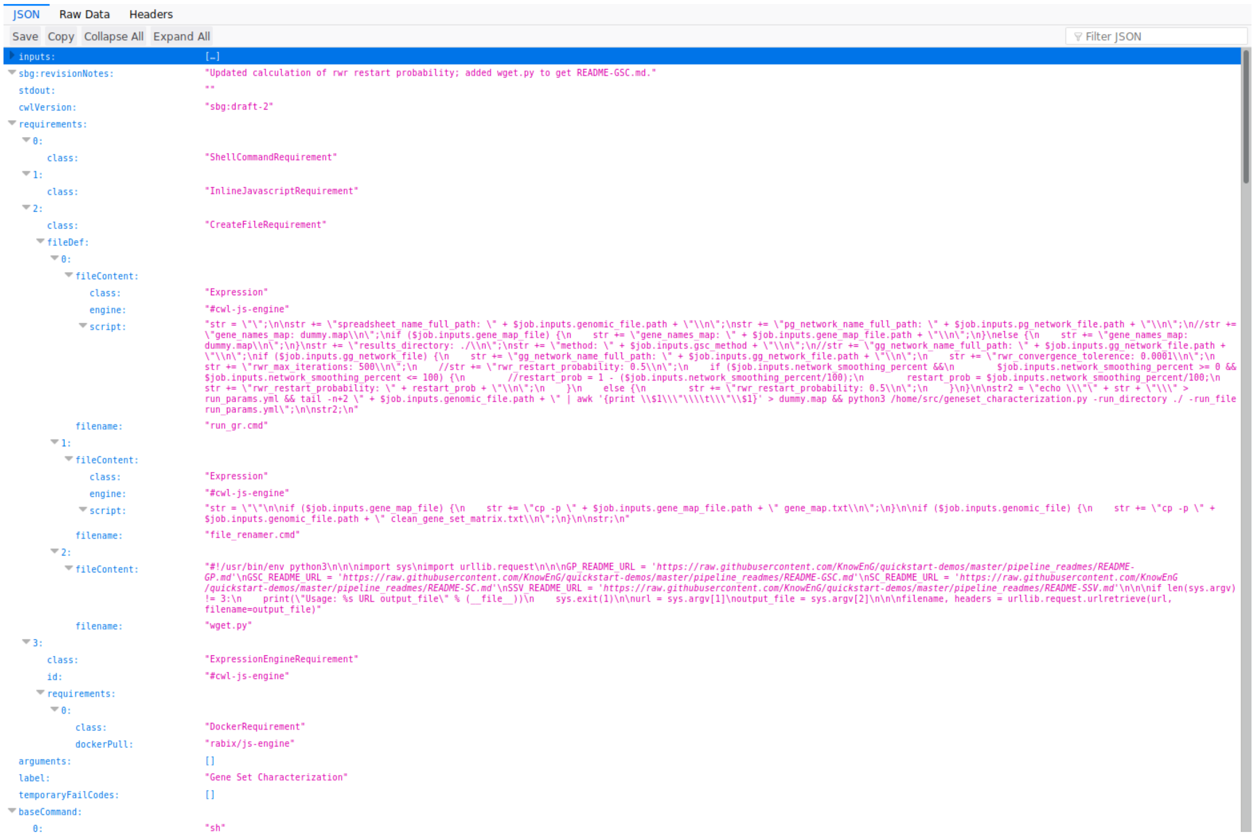

Figure Z. CWL for the GSC Tool.

CWL can be in JSON or YAML format, and includes description of the inputs and outputs of the app, and the specification of the command line (e.g., the base command and arguments) for command line tools or the steps in the workflow.

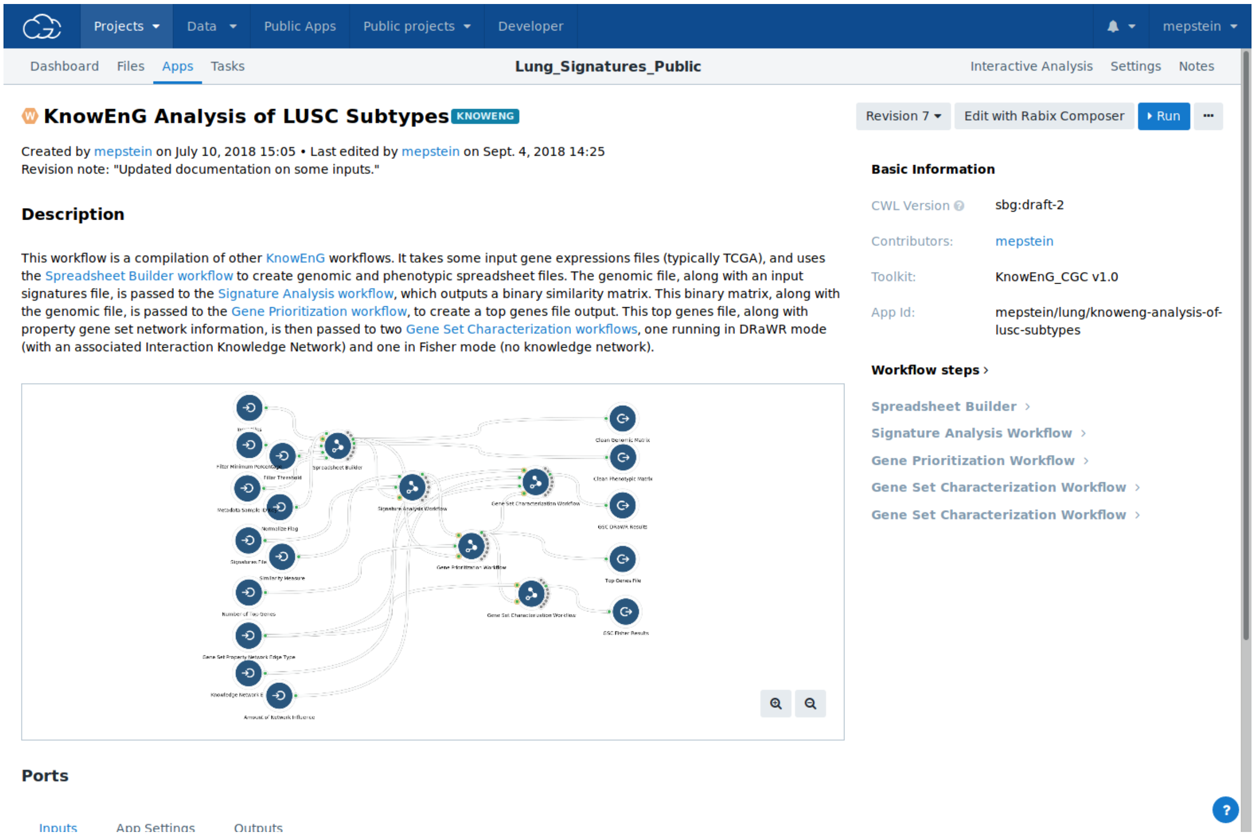

Figure AA. Workflow for the Analysis of LUSC Subtypes.
This page can be seen at [https://cgc.sbgenomics.com/public/apps#mepstein/lung/knoweng-analysis-of-lusc-subtypes/]. The documentation for the KnowEnG Analysis of LUSC Subtypes (KALS) workflow app on the SB-CGC.

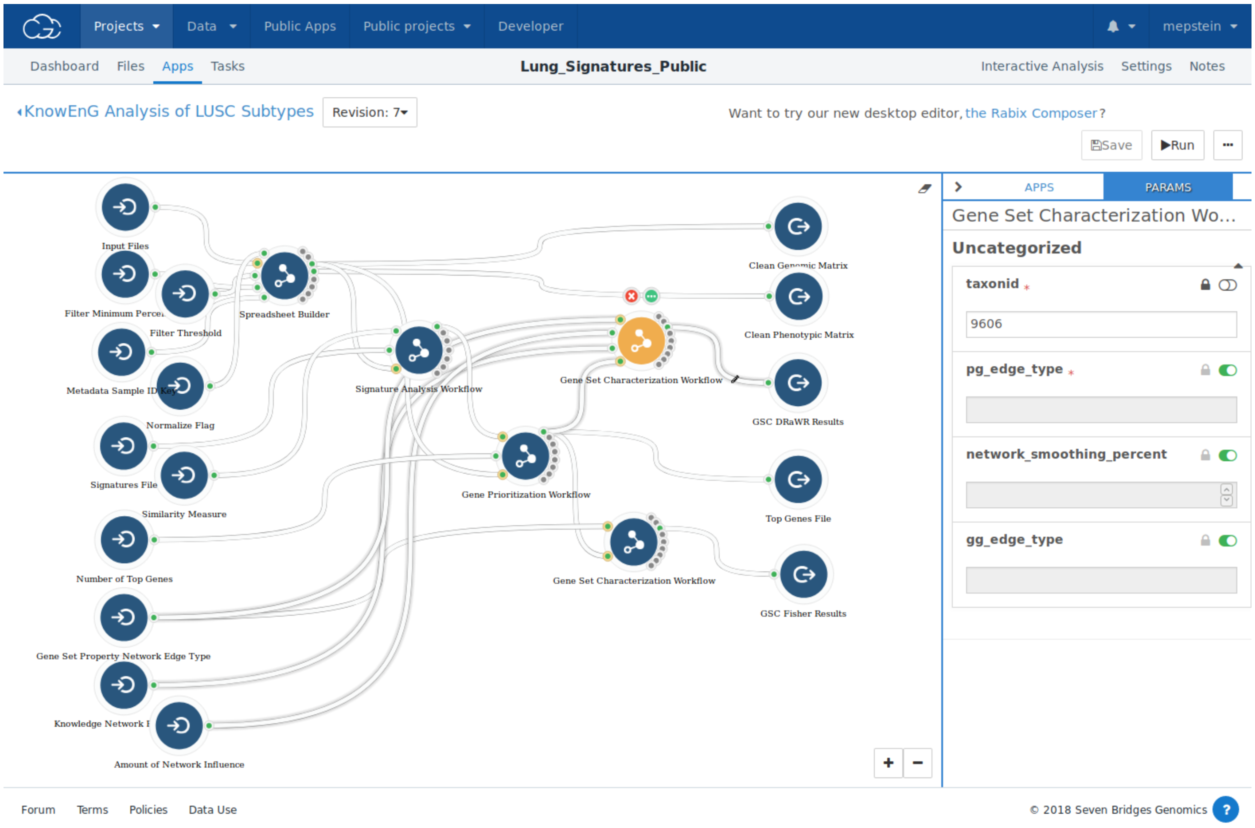

Figure AB. Editing the KALS Workflow.
The KALS workflow combines the Spreadsheet Builder workflow, the Signature Analysis workflow, the Gene Prioritization workflow, and two instances of the Gene Set Characterization workflow.

Appendix I: Developing a New Analysis Pipeline for the KnowEnG System

Overview

In this supplementary appendix, we will break down the steps that are typically required to create a new analysis software tool and integrate it as pipeline in the KnowEnG suite of access modalities including:

1. our Jupyter Notebook server (Appendix E in S2 File),
2. as a CWL tool on the Dockstore and on the Seven Bridges Cancer Genomics Cloud (Appendix H in S1 File),
3. our public KnowEnG Platform web server (Appendix A in S1 File), and
4. with our AWS CloudFormation Template (Appendix C in S1 File).

Only some subset of these steps may be required depending on the how the tool will be used, the level of coding familiarity of its target users, and the location and security issues related to the data to be analyzed. In the following sections, we hope to highlight the main considerations

of adding the tool for each modality as well as highlight examples (as bracketed links) showing what was done to add our Gene Prioritization pipeline to KnowEnG.

Command Line Tool

The first step to adding a new tool as a KnowEnG pipeline is to create the tool as a command line executable. Since this executable will be run in a Docker container in most cases, the language with which the tool is written and the specific environment in which it was compiled or run generally will not matter. For compatibility with our Jupyter notebook only, we recommend tools that are written in Python or R and developed on Linux systems, because it would allow the users of the notebook to use the interactive environments to edit, test, and run components and functions of the overall analysis tool.

Whatever the language the tool is written in, best practices should be observed [10]. These include and are not limited to:

- A program README with instructions for running a simple example [https://github.com/KnowEnG/Gene_Prioritization_Pipeline/blob/master/README.md]
- Writing meaningful and clean code with discrete and documented functions and minimal duplication [https://github.com/KnowEnG/Gene_Prioritization_Pipeline/blob/master/src/gene_prioritization_toolbox.py]
- Using well maintained libraries, like sciPy or pandas for Python
- Creating and running tests for correctness and performance [https://github.com/KnowEnG/Gene_Prioritization_Pipeline/tree/master/test]
- Versioning code and committing it to a code repository [https://github.com/KnowEnG/Gene_Prioritization_Pipeline/commits/master]

There are some additional considerations to make if you plan to support knowledge-guided analysis and use the KnowEnG Knowledge Network (Appendix A in S2 File). You will need to check whether your tools will process the network edge files correctly. Network edge files are assumed to be tab-separated files with at least three columns where each row contains information about an edge in the network. The first column is treated as the source nodes of the network and the second column is treated as the target nodes. The third column is required and assumed to have the weights of each edge. The weights are assumed to be non-null and greater than zero. If your tool requires a different network format for knowledge-guided analysis, you may have to write a format converter in order to be compatible with all the public networks stored in the Knowledge Network.

Additionally, there are some considerations that may make your tool easier to integrate into the public KnowEnG Platform server. While these recommendations are not required for the platform integration, they will enable greater reusability of functions already implemented in the platform for handling submitted pipeline runs.

- **Parameter Passing**: The pipeline configurations and launchers in the KnowEnG platform write nearly all pipeline parameters to a yaml file that is to be read and processed by the tool executable. This creates a record of the parameters for each run

and simplifies the command line structure required for starting each run. [https://github.com/KnowEnG/Gene_Prioritization_Pipeline/blob/master/data/run_files/BENCHMARK_3_GP_net_pearson.yml]

- **Command Line Structure**: The KnowEnG platform also uses a standardized format for the command line call necessary to launch the run of a tool. The format has three components: 1) the location of the executable, 2) the location of the working directory in which to process the run, 3) and the location of the parameters yaml file described previously.
- **Error Logging**: The KnowEnG platform is set up to process and pass error messages on to users by reading a specially formatted yaml log file. This file is typically a list of string messages, with information about the run flagged with the prefix string "INFO: ", harmless warnings flagged with the prefix string "WARNING: ", and errors captured and flagged with the prefix string "ERROR: ". An example log file is seen here [https://github.com/KnowEnG/Data_Cleanup_Pipeline/blob/master/data/verification/gene_prioritization_pipeline_pearson/log_gene_prioritization_pipeline.yml].
- **Parallelization**: On the KnowEnG platform, the runs are distributed to individual nodes of a compute cluster as Docker-based tasks. These Docker tasks can be configured to use multiple CPUs that may be available on the compute node. If the new tool makes use of multiple threads, you will want to create top level parameters for the tool that control the amount of resources that the tool will use on its assigned compute node.
- **Format of Output Files**: Finally, the KnowEnG platform is designed to 'handover' (see Fig 2A) the outputs of one pipeline as the inputs of another. Because of this type of functionality, you may want to design the final outputs and useful intermediates of your tool to match the formats that KnowEnG has established for genomic and phenotypic spreadsheets. More information about these formats can be found at [https://github.com/KnowEnG/quickstart-demos/blob/master/pipeline_readmes/README-DataPrep.md]

It is also possible to add existing tools as pipelines to KnowEnG rather than writing tools from scratch. In this case, you may want to create simple wrapper scripts for the existing tools that address the issues raised above.

Dockerized Pipeline Tool

Docker [https://www.docker.com/] provides containerization, a form of virtualization that allows for capturing all of the system, environment, library, and code dependencies an application needs in a standard, lightweight object. Docker containers can be executed in many computation environments. After creating or selecting a command line tool for addition to KnowEnG as a pipeline, it is necessary to save that tool inside a Docker image.

The first step of this process is to build a simple Dockerfile, a text document that contains all the commands necessary to assemble a Docker image. Best practices for creating Dockerfiles are found here [https://docs.docker.com/v17.09/engine/userguide/eng-image/dockerfile_best-practices/]. The Dockerfile typically begins by selecting the appropriate base image and installing the additional and necessary libraries and packages. It usually continues by modifying

the environment variables and copying or installing the application code for the tool. For KnowEnG Python-based tools, this often just involves cloning the GitHub repository into the image. Finally, in the Dockerfile you can specify the default command to execute when that image is run. The Dockerfile for Gene Prioritization is only a few lines long [https://github.com/KnowEnG/Gene_Prioritization_Pipeline/blob/master/build/docker/Dockerfile].

After creating the Dockerfile, you will want to build the corresponding Docker image and version it with a tag identifier. KnowEnG images are frequently tagged by the date they were built. Then, simply create a README for using the Docker image and publish it at an image repository [https://hub.docker.com/r/knowengdev/gene_prioritization_pipeline]. Wherever Docker is installed, the new image can be pulled and run, and the analysis tool inside executed.

Jupyter Notebook Server

In (Appendix E in S2 File), we introduced the KnowEnG JupyterHub server instance [https://knowtebook.knoweng.org/hub/login] that gives user access to secure notebooks for web-enabled interaction of KnowEnG data and code. In this subsection, we will quickly walk through the process of how a new pipeline tool could be added to our JupyterHub Server. Adding a pipeline to the Knowtebook server can be useful for quickly prototyping and accessing pipelines and data through a web browser with command line capabilities. This access modality is probably most useful when the tool itself is written in Python or R as this allows the notebook users to manipulate the tool and its functions in the Python/R kernel. The process primarily relies on adding the tool code and libraries to the Docker image that launches the customized JupyterHub Server.

The first step of adding the new tool is to update the customized base image our Knowtebooks server uses. For this, we just need update the Dockerfile by copying in the new tool code (often done by cloning the GitHub repo) and by installing any libraries and packages needed by the tool [https://github.com/KnowEnG/KnowEnG_All_Pipelines/blob/master/build/docker/Dockerfile]. Once we rebuild our base Docker image [https://hub.docker.com/r/knowengdev/jupyter_notebooks], we can then work on creating a simple IPython notebook that provides some basic instructions for setting up the inputs for the tool on the server and then provides a compute cell that shows sample code for executing an example run of the analysis tool [https://github.com/KnowEnG/KnowEnG_All_Pipelines/blob/master/data/server_notebooks/feature_prioritization_notebook.ipynb]. The final step is to create the final customized JupyterHub image by updating its build file to copy in any of the necessary example data files and the new IPython notebook [https://github.com/KnowEnG/KnowEnG_All_Pipelines/blob/master/Dockerfile]. Once the final image is pushed to public repos, anyone can launch the customized JupyterHub Notebook server and have access to all pipelines and sample datasets. This access mode is especially useful when users want to programmatically rather than manually set up many analysis runs, as is commonly done in parameter sweeps.

CWL Wrapped Tool and Seven Bridges Cancer Genomics Cloud

If the new tool is to be added to the Seven Bridges Cancer Genomics Cloud (SB-CGC, see Appendix H in S1 File), then there are a few steps that need to be undertaken.

First, you need to write a Common Workflow Language (CWL) description of the new tool. CWL [https://www.commonwl.org/] is a language for describing analysis tools and workflows. It specifies the details of the inputs, outputs, and execution steps of the tools. This allows for making the tools portable and scalable across of variety of software and hardware environments. More information about the best practices for writing a CWL description for an analysis tool can be found here [https://doc.arvados.org/user/cwl/cwl-style.html], but at a high level there are a few main parts:

- **Docker image**: the name of the Docker image that contains the analysis tool and the appropriately configured running environment
- **Resource minimums**: the CWL description can contain the memory, disk, and CPU minimum requirements for running the analysis tool and the CWL executor can communicate this to Docker when launching the corresponding container
- **Inputs**: identifiers, types, and defaults for the input files and parameters. Additional documentation is also helpful.
- **Outputs**: identifiers and output types are required, as well as regular expressions for binding output files
- **Base command**: the command line executable for the analysis tool from inside the Docker container. Often for KnowEnG tools, our base command also contains a shell script that will convert the CWL parameter specifications into our standard KnowEnG parameter yaml file.

An example CWL description for the Gene Prioritization pipeline is here [https://github.com/KnowEnG/KnowEnG_CWL/blob/master/GenePrioritization/CWL/gp_runner.cwl]. Once a new pipeline has a CWL description, it can be submitted to the Dockstore [https://dockstore.org/], a repository for executable tools for science. The CWL description of a new tool provides machine parsable instructions on how to run it and allows for increased automation in the launching of tasks/runs.

The Cancer Genomics Cloud [https://cgc.sbgenomics.com/], built by Seven Bridges Genomics as part of the National Cancer Institute's Cancer Genomics Cloud pilot program, is a cloud-based environment that hosts large genomic data sets (e.g., TCGA) as well as genomic analysis tools. These tools and workflows can be executed on the CGC, which uses Amazon's AWS behind the scenes. There is an entire note (Appendix H in S1 File) on running and porting analysis tools to the SB-CGC. Once you have built your Docker image and your CWL description, you are most of the way there. The only major remaining step is to use the SB-CGC provided tools (such as the Rabix integrated development environment (https://rabix.io/)) to import your pipeline into their platform. Additional tools may need to be developed in order to connect your pipeline with the data files available in the SB-CGC. In this case, you will have to consider the entire end-to-end workflow that is needed and build CWL-described tools and connect them in a CWL-described workflow in the SB-CGC. However, completing this process

makes your tool available on a scalable compute environment that has access to a large specialized dataset and the specialised methods for querying specific subset of the data. The analyses run in the SB-CGC are also always reproducible and easily shared with colleagues.

KnowEnG Platform

Introducing a new pipeline to the KnowEnG platform involves three points of integration:

- The **"launcher,"** where users select data and enter parameters for their analyses,
- The **job worker,** which queues user-initiated analyses, prepares data and parameters for handoff to pipelines, executes pipelines as Docker containers, and processes the results, and
- The **visualization**, consisting of user-interface elements on screen and the web services providing them with data.

The effort required at each of these integration points depends on whether the pipeline to be added is either (1) a new variant of a kind of analysis already present in the KnowEnG platform--for example, an alternative algorithm for prioritizing genes--that is substantially compatible with an existing launcher, job worker, and visualization, or (2) a kind of analysis new to the KnowEnG platform.

In the first case, where the new pipeline is a variant of a kind of analysis already present in the KnowEnG platform, the tasks are as follows:

- **Launcher:** The launcher for the existing analysis type will need to be modified so that users have the option of selecting among all pipelines of the type, including the existing pipeline(s) and the new pipeline. If the new pipeline also introduces new user-controlled parameters, they will have to be added to the launcher. These changes might in turn call for edits and additions to the help text that appears in the launcher margin. To simplify the task of creating and maintaining launchers, KnowEnG provides a library of launcher widgets, and a pipeline's launcher specification consequently consists of a single TypeScript file detailing which widgets to use, what values they should accept, and how widgets interact with each other. https://github.com/KnowEnG/platform/blob/master/client/src/models/knoweng/pipelines/FeaturePrioritization.ts]
- **Job worker:** The job worker for the existing analysis type will need to handle the new parameters arriving from the modified launcher. Commonly, this will entail converting the pipeline type to a Docker image tag and saving new fields to the parameter yaml file passed to the Docker container. If the new pipeline departs from the existing pipelines of the same type in how it formats its results or how it names its output files, minor edits will have to be made to accommodate the changes. [https://github.com/KnowEnG/platform/blob/master/nest_py/knoweng/jobs/pipelines/feature_prioritization.py]
- **Visualization:** In our experience, adding a new pipeline of an existing kind entails few changes to the visualization. Generally the web service, data model, and major user-

interface elements do not need modification. Sometimes minor changes are necessary to labeling and number-scaling, conditioned upon the choice of pipeline.

In the second case, where the pipeline is the first of its kind in the KnowEnG platform, the tasks are as follows:

- **Launcher:** As described in the first case above, launchers are specified using a library of widgets. A new launcher specification will need to be created alongside, and following the example of the other pipelines https://github.com/KnowEnG/platform/blob/master/client/src/models/knoweng/pipelines/FeaturePrioritization.ts]. The new specification must then be added to the directory of available pipelines, which entails adding one entry to an array [https://github.com/KnowEnG/platform/blob/master/client/src/services/knoweng/PipelineService.ts]. For users' convenience, you may also wish to provide example data files [https://github.com/KnowEnG/platform/blob/master/client/data/demofiles.json], which are incorporated in the deployment process via a seed job [https://github.com/KnowEnG/platform/blob/master/nest_py/knoweng/jobs/knoweng_seed_job.py], and even a demonstration video, which is exposed to the user interface with an entry in a data file [https://github.com/KnowEnG/platform/blob/master/client/data/youtube.json].
- **Job worker:** A job worker usually consists of a single Python module (script) subclassing a predefined superclass and implementing several standard methods. Job worker modules are stored in a single Python package (directory) [https://github.com/KnowEnG/platform/tree/master/nest_py/knoweng/jobs/pipelines], where you will find examples, and incoming job requests are dispatched via a case in the queue consumer [https://github.com/KnowEnG/platform/blob/master/nest_py/knoweng/jobs/worker_app.py]. From the job worker's point of view, queuing, status reporting, and communication with Kubernetes are handled automatically.
    - Each analysis type will store results in one or more database tables, with one or more web service endpoints providing an API, for efficient access from the visualization. For typical permissions--in which only the user who created the data is allowed to see it--tables can be defined with one or more simple subclasses [https://github.com/KnowEnG/platform/blob/master/nest_py/knoweng/data_types/feature_prioritizations.py] and will automatically be added to the database during deployment by adding a line in the directory of schemas [https://github.com/KnowEnG/platform/blob/master/nest_py/knoweng/data_types/knoweng_schemas.py]. Web service endpoints are automatically created by adding lines to the directory of CRUD (create, read, update, delete) endpoints [https://github.com/KnowEnG/platform/blob/master/nest_py/knoweng/flask/knoweng_flask.py]. The job worker can write to the new table by declaring new methods in a class of database utility methods [https://github.com/KnowEnG/platform/blob/master/nest_py/knoweng/jobs/db_utils.py].

    - Each pipeline invocation should also produce a zip archive of results for user download. The zip archive itself is created by the job worker module. Our convention is to include a README file with each zip archive; the README is incorporated in the deployment process via the seed job [https://github.com/KnowEnG/platform/blob/master/nest_py/knoweng/jobs/knoweng_seed_job.py]. The new analysis type can be added to the web service that provides zip archives with a new dictionary entry [https://github.com/KnowEnG/platform/blob/master/nest_py/knoweng/flask/nest_endpoints/job_downloads_endpoint.py].
- **Visualization:** A visualization is implemented as a new Angular module [https://github.com/KnowEnG/platform/blob/master/client/src/components/knoweng/results/feature_prioritization/FPModule.ts], which must be imported in the parent module for all results visualizations [https://github.com/KnowEnG/platform/blob/master/client/src/components/knoweng/results/ResultsModule.ts]. Elements from other KnowEnG results visualizations are suitable for reuse and can be incorporated. Typically, the visualization will communicate with new web service endpoints via a new data service [https://github.com/KnowEnG/platform/blob/master/client/src/services/knoweng/FeaturePrioritizationService.ts], which needs to be added to the data providers with a new entry in an array [https://github.com/KnowEnG/platform/blob/master/client/src/components/knoweng/CoreModule.ts], and which will model data with one or more new classes [https://github.com/KnowEnG/platform/blob/master/client/src/models/knoweng/results/FeaturePrioritization.ts]. The new pipeline should also receive new cases as one line in the results display area [https://github.com/KnowEnG/platform/blob/master/client/src/components/knoweng/results/ResultsVisualization.html] and one line in the job summary widget [https://github.com/KnowEnG/platform/blob/master/client/src/components/knoweng/results/common/JobSummary.html], and it should receive a new color to distinguish it visually from other jobs when displayed in tables [https://github.com/KnowEnG/platform/blob/master/client/src/models/knoweng/Job.ts].

KnowEnG CloudFormation Template

After adding the new pipeline to the KnowEnG platform as described above, deploying the platform via CloudFormation Template is straightforward:

1. Edit the docker-compose.yml [https://github.com/KnowEnG/platform/blob/master/docker-compose.yml] to replace the “image” values associated with nest_flask_i and nest_jobs_i; you will need to choose values that match your Docker Hub username.
2. Rebuild the KnowEnG Docker images with your changes by invoking “compose.sh build && compose.sh push” from the repository root directory.
3. Push the newly built images to Docker Hub.
4. Deploy an instance of the KnowEnG platform using the usual CloudFormation Template. (This step is optional; if you want to update a running, previously deployed instance, skip this step.)

5. From a command prompt in the platform instance's bastion host (see https://github.com/KnowEnG/Kubernetes_AWS/tree/master/cloudformation), download https://raw.githubusercontent.com/KnowEng/Kubernetes_AWS/master/cloudformation/nest.cfn.yaml to the local disk. Edit the downloaded file to change the image names "knowengdev/nest:flask" and "knowengdev/nest:jobs" with the image tags you selected in step 1. Run "kubectl apply -f nest.cfn.yaml", and Kubernetes will restart the system with your changes.

References

1. Baker MP, Bushell C. After the storm: Considerations for information visualization. IEEE Computer Graphics and Applications. 1995;15(3):12-5.
2. Tufte ER, McKay SR, Christian W, Matey JR. Visual explanations: images and quantities, evidence and narrative. AIP; 1998.
3. Blatti C, Sinha S. Characterizing gene sets using discriminative random walks with restart on heterogeneous biological networks. Bioinformatics. 2016;32(14):2167-75. doi: 10.1093/bioinformatics/btw151. PubMed PMID: 27153592; PubMed Central PMCID: PMCPMC4937193.
4. Hamosh A, Scott AF, Amberger JS, Bocchini CA, McKusick VA. Online Mendelian Inheritance in Man (OMIM), a knowledgebase of human genes and genetic disorders. Nucleic acids research. 2005;33(suppl_1):D514-D7.
5. Liberzon A, Subramanian A, Pinchback R, Thorvaldsdottir H, Tamayo P, Mesirov JP. Molecular signatures database (MSigDB) 3.0. Bioinformatics. 2011;27(12):1739-40. doi: 10.1093/bioinformatics/btr260. PubMed PMID: 21546393; PubMed Central PMCID: PMCPMC3106198.
6. Barrett T, Wilhite SE, Ledoux P, Evangelista C, Kim IF, Tomashevsky M, et al. NCBI GEO: archive for functional genomics data sets—update. Nucleic acids research. 2012;41(D1):D991-D5.
7. Emad A, Ray T, Jensen TW, Parat M, Natrajan R, Sinha S, et al. An epithelial-mesenchymal-amoeboid transition gene signature reveals molecular subtypes of breast cancer progression and metastasis. bioRxiv. 2017:219410. doi: 10.1101/219410.
8. Hoadley KA, Yau C, Wolf DM, Cherniack AD, Tamborero D, Ng S, et al. Multiplatform analysis of 12 cancer types reveals molecular classification within and across tissues of origin. Cell. 2014;158(4):929-44. doi: 10.1016/j.cell.2014.06.049. PubMed PMID: 25109877; PubMed Central PMCID: PMCPMC4152462.
9. The Cancer Genome Atlas Research N, Kim J, Bowlby R, Mungall AJ, Robertson AG, Odze RD, et al. Integrated genomic characterization of oesophageal carcinoma. Nature. 2017;541:169. doi: 10.1038/nature20805

https://www.nature.com/articles/nature20805#supplementary-information.

10. Wilson G, Bryan J, Cranston K, Kitzes J, Nederbragt L, Teal TK. Good enough practices in scientific computing. PLoS computational biology. 2017;13(6):e1005510.
