# Supplementary material for: Knowledge-guided analysis of "omics" data using the KnowEnG cloud platform
Source: PLoS Biol. 2020 Jan 23;18(1):e3000583. doi: 10.1371/journal.pbio.3000583 (PMC6977717; doi:10.1371/journal.pbio.3000583)
Supplement: S2 File — Seven appendices describing the data, pipelines, methods, and additional analyses that relate to the first case study of performing sample clustering on cancer patient data. (PDF) [file pbio.3000583.s002.pdf]

S2 File: Methods for Sample Clustering Case Study

Table of Contents

Table of Contents 1
Appendix A: Constructing the Knowledge Network 3
Overview 3
Methods 4
Resources 4
Figures 4
Figure A. Overview of the Knowledge Network Build Pipeline. 4
Figure B. Parallelized Task Execution Example. 5
Tables 5
Table A. Species in Knowledge Network. 5
Table B. External Public Data Repositories. 5
Appendix B: Description of the TCGA PANCAN Dataset 5
Methods 5
Tables 6
Table C. TCGA Datasets. 6
Table D. TCGA Mutation Data Samples. 6
Appendix C: Sample Clustering on TCGA Mutation Dataset 6
Overview 6
User Inputs 7
User Parameters 8
Data Preprocessing 8
Description of Algorithm 9
Pipeline Outputs 10
Methods 11
Resources 12
Figures 13
Figure C. Sample of “omics” Spreadsheet 13
Figure D. Sample of Phenotypic Spreadsheet. 14
Figure E. Overview of Knowledge-Guided Sample Clustering. 14
Figure F. Survival Analysis of “sc_noNet” Clustering. 15
Figure G. Survival Analysis of “sc_sText” Clustering. 15
Figure H. Survival Analysis of “tcga_mut” Clustering. 16
Tables 16
Table E. PANCAN12 Mutation Sample Clusterings. 16
Table F. PANCAN12 Mutation Samples Cluster Assignments. 16
Table G. Enrichment between Clusters. 16
Appendix D: Characterization of Knowledge Guided Mutation Subtypes 17
Background 17
Results 17
Tables 18
Table H. 'hnInt' Clusters by Disease Type. 18
Table I. 'hnInt' Cluster Specific Genes. 18
Table J. Top Pathways Enriched in Genes Specific for 'hnInt' Clusters. 19

Appendix E: KnowEnG Jupyter Tools and Combining Multi-omics Clusterings 19
Overview 19
User Experience 19
Description of Algorithm 20
Methods 20
Resources 21
Tables 21
Table K. Combined Multi-omics Clustering Matrix. 21
Appendix F: COCA Analysis of Multi-omics TCGA Data 21
Methods 21
Figures 22
Figure I. Survival Analysis of "tcga_coca" Original COCA 22
Tables 22
Table L. Cluster Assignments for COCA Survival Analysis. 22
Table M. Selected Gene Copy Number Variation Data. 23
Appendix G: Sample Clustering on METABRIC Transcriptomic Dataset 23
Methods 23
Data Collection 23
Clustering of samples based on the EMT signature 23
Figures 24
Figure J. Survival probabilities of PAM50 subtypes of METABRIC samples. 24
Figure K. Survival Analysis using EMT signature and Standard Sample Clustering. 24
Figure L. Survival Analysis using EMT signature and Sample Clustering with hnInt. 25
Figure M. Survival Analysis using EMT signature and Sample Clustering with sText. 25
References 26

Appendix A: Constructing the Knowledge Network

Overview

The KnowEnG Center has developed and published a pipeline for collecting and harmonizing prior knowledge about genes and proteins and formatting that data for knowledge-guided analysis pipelines. This pipeline organizes these datasets as a massive heterogeneous network, called the Knowledge Network. The goal of this pipeline is to incorporate as many external sources and make the process of transforming their newest datasets into the Knowledge Network as simple, fast, and transparent as possible (Figure A in S2 File). Roughly, there are four stages to the Knowledge Network Build Pipeline:

1. SETUP, which downloads and imports the latest Ensembl [1] gene name mapping databases into a local Redis [https://redis.io/] database
2. PARSE, which downloads, parses, and performs entity mapping on the latest version of external public database collections of gene interactions and annotations,
3. IMPORT, which stores the harmonized data and provenance information into a MySQL [https://www.mysql.com/] database, and
4. EXPORT, which outputs the interactions and entity maps in the Knowledge Network as flat files formatted for the KnowEnG analysis tools.

The Knowledge Network Build Pipeline is decomposed into many small tasks by species or external public database that we run in parallel across a cloud environment (Figure B in S2 File). We use Apache Mesos [http://mesos.apache.org/] to manage the resources and scheduling of these tasks. Apache's Marathon [https://mesosphere.github.io/marathon/] framework is given responsibility for deploying the Redis and MySQL databases during the build, and its Chronos [https://mesos.github.io/chronos/] framework manages the dependencies between the stages of the build and the many tasks. All tasks are run within an environment provided by our Docker Image, KN_Builder. The build pipeline is designed to run from start to finish with a single command line so it can be automated and regular updates be published to stable location such as AWS S3 [https://aws.amazon.com/s3/].

The design philosophy of the Knowledge Network Build Pipeline is to preserve the original annotations and interactions from the public data sources as carefully as possible. Interactions between entities where at least one cannot be mapped with the Ensembl resources are marked and prevented from publication in the final exported Knowledge Network. If multiple evidences exist for the same type of relation, the strongest one is exported and the others are marked as redundant. Metadata relating to the original interaction and the mapping decisions are stored in our MySQL database, as well as the licensing and citation information from the original source. For the final export, subnetworks of a single species and type are reported as 1) a list of weighted, undirected edges using the Ensembl stable identifiers along with references back to their original source line, 2) a map from the stable identifies to common aliases and descriptions, and 3) a file that describes the provenance of the contained data.

Methods

For the results in this paper, we performed a complete build of the Knowledge Network in June of 2017, tagged as KN-20rep-1706. This build was focused on twenty species available from Ensembl of particular research interest (Table A in S2 Data). The build extracted data from 13 external public data sources listed in Table B in S2 Data. This build extracted 137 different datasets that contained 185 different edge types. The combined Knowledge Network contained 405,439 gene nodes, 178,493 annotation/property nodes, and 476,816,989 edges. In the analysis in this paper, we only use the human subnetworks of the Knowledge Network (taxon id 9606). Specifically, we use the edge types of the Knowledge Network that represent integrated interaction scores from humanNet (hn_IntNet) [2], text mining scores from STRING (STRING_textmining) [3], annotations from Gene Ontology (gene_ontology) [4], and pathway gene sets from Enrichr (enrichr_pathway) [5].

Resources

Knowledge Network Build Pipeline

Docker Image [https://hub.docker.com/r/knoweng/kn_builder/]
GitHub Repository for Launcher [https://github.com/KnowEnG/KnowNet_Pipeline_Tools]
GitHub Repository for Container [https://github.com/KnowEnG/KN_Builder/]
Build Pipeline Documentation [http://knowredis.knoweng.org/#]
Knowledge Network Resources [https://knoweng.org/kn-tools/]

KN-20rep-1706 Knowledge Network

High Level Content Summary [https://knoweng.org/kn-overview/]
Detailed Content Summary [https://knoweng.org/kn-data-references/]
Downloadable Content Links
[https://github.com/KnowEnG/KN_Fetcher/blob/master/Contents.md]

Figures

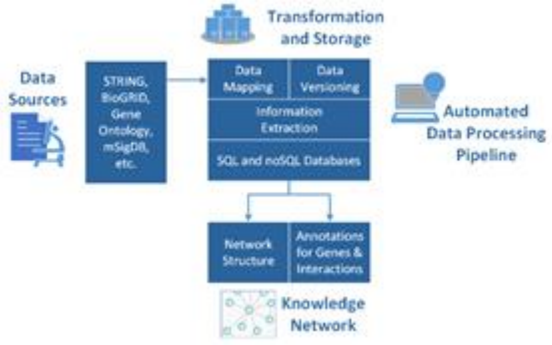

Figure A. Overview of the Knowledge Network Build Pipeline.
Overall flow of data from original external public repositories to final exported KnowEnG Knowledge Network.

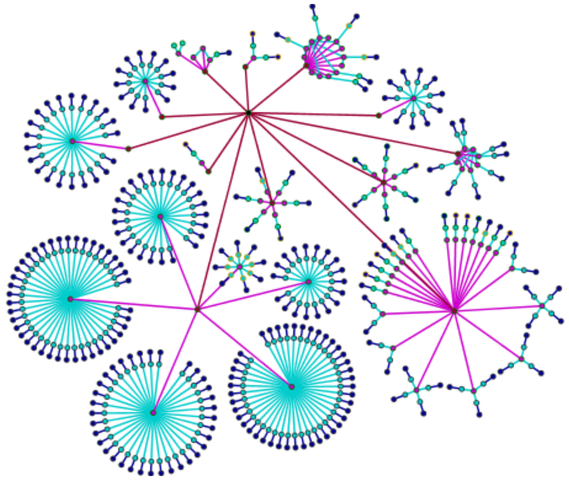

Figure B. Parallelized Task Execution Example.
Each node in this diagram is a containerized task in the Build Pipeline and the edges represent dependencies between tasks. The node color shows the type of task: check external source (red), download external file (magenta), parse external data format (cyan), and map interaction entities (dark blue). The node borders show an example status of each task: successful (green), running (yellow), or queued (black). For a particular size of the cloud deployment, the diagram shows how tasks are partitioned and can be executed simultaneously.

Tables

Table A. Species in Knowledge Network.
For each species, lists the taxon identifier for the species as well as the number of Knowledge Network gene nodes, total number edges, and different data source files related to that species.

Table B. External Public Data Repositories.
For each data source, lists the number of Knowledge Network property/annotation nodes and total number edges derived from that sources as well as the PubMed identifier.

Appendix B: Description of the TCGA PANCAN Dataset

Methods

In our first two case studies, we performed our analysis on multi-omics tumor sample data from the TCGA PANCAN analysis of 12 cancer types [6]. For five types of omics data, we downloaded the omics spreadsheet and the clinical annotations from the UCSC Cancer Genome Browser [7] in September 2017. The miRNA data from the original study was not available here. We extracted the overall survival days and indicator data from the clinical annotations, "_OS" and "_OS_IND". We also relied on the three clusterings of the samples from the original TCGA publication: their disease annotation ("_primary_disease"), their pathway

guided clustering ("_PANCAN_mutation_PANCAN"), and their Cluster-Of-Cluster-Assignments clustering ("_PANCAN_Cluster_Cluster_PANCAN"). Information about these omics files, the number of samples, and the number of omics features can be found in Table C in S2 Data.

For the first case study, we examined the sample mutation data in particular, which contained mutation data for 39,674 genes on 3276 samples (Table D in S2 Data). For every (gene, sample) pair in this genomic spreadsheet, there is a "1", indicating that in that sample there was at least one nonsynonymous somatic mutation in the coding sequence of that gene or there is a "0", indicating otherwise. For the Cluster-Of-Cluster-Assignment analysis, we also used the omics data of four of the remaining data types ("CGA_PANCAN12_RPPA_RBN-2015-01-28","TCGA_PANCAN12_exp_HiSeqV2-2015-01-28","TCGA_PANCAN12_hMethyl-2015-01-28","TCGA_PANCAN12_genecopynumber-2015-01-28") and the clustering information from the clinical annotations for miRNA, "_PANCAN_miRNA_PANCAN". The number of clusters in each of the original six clusterings performed by TCGA can be seen in Table C in S2 Data. In the second case study for gene prioritization, we focused on the population-normalized log2(RPKM + 1) gene expression data from TCGA_PANCAN12_exp_HiSeqV2-2015-01-28 for 16,114 genes in 3598 samples.

Tables

Table C. TCGA Datasets.

For each omics data type (rows), shows the acronym used for that data type, the number of omics features measured, the number of samples profiled, and the number of clusters produced from the clustering of the data in the original paper.

Table D. TCGA Mutation Data Samples.

For each primary disease type (rows), shows the acronym used for that disease and the number of samples from a specific "sample location type" (columns).

Appendix C: Sample Clustering on TCGA Mutation Dataset

Overview

The KnowEnG Sample Clustering pipeline is a data-mining tool that performs the clustering of samples based on the similarity of their feature profiles. This type of analysis is useful in bioinformatics when given a large collection of samples described by their "omics" (genomic, transcriptomic, etc.) profiles and you want to find a limited number of subtypes (or sample clusters). The Sample Clustering pipeline is available in two modes: 1) a "knowledge-guided" mode that integrates gene-based feature profiles with prior knowledge from the KnowEnG Knowledge Network and 2) a "standard" mode that performs the more common without the prior knowledge. In both modes, these omic subtypes may then be correlated with the phenotypes of the samples in order to understand and use them for various prediction tasks (e.g. prognosis,

treatment outcome). The Sample Clustering pipeline supports the common paradigm of consensus clustering [8], where subsets of the data are clustered independently many times and the global co-occurrence of samples in the same cluster are tracked. This mode is compute intensive, but it allows for a more robust clustering of the data that is less reliant on the starting or convergence conditions of an individual clustering.

Knowledge-Guided Sample Clustering

The knowledge-guided mode of the Sample Clustering pipeline is inspired by Network Based Stratification [9]. The fundamental idea of this analysis is that it is difficult to accurately assess the distance between samples in sparse genomic datasets in which the value assigned to most genomic features for a sample is zero (e.g. a dataset that records the genes with somatic mutations in cancer patients). The hypothesis is that a clustering similarity measure becomes more reliable when you allow the sparse sample features to propagate across local neighborhoods in a known gene-gene interaction network. The KnowEnG Sample Clustering pipeline enables users to run this network-guided analysis on their gene-level omics spreadsheets with many different types of interaction networks.

Standard Sample Clustering

The KnowEnG Sample Clustering pipeline supports k-means and hierarchical clustering of the sample columns of the omics spreadsheet in its standard mode. In this mode, the features are not required to be related to genes and the Knowledge Network is ignored.

User Inputs

The Sample Clustering pipeline has two primary inputs:

1. a required “omics spreadsheet” which will have its samples divided into subtypes/clusters based on the distance between their feature profiles, and
2. an optional “phenotype spreadsheet” that will contain numerical or categorical information on the samples that will be used to describe how well the identified clusters associate with known phenotypes of interest.

The “omics” spreadsheet is expected to be a matrix of numeric values with named rows representing features and named columns representing samples (Figure C in S2 File). *For the knowledge-guided mode of Sample Clustering, the features must correspond to genes and be labeled with common names or identifiers, and the matrix must contain only non-negative values*.

The phenotypic spreadsheet is expected to be a matrix where the named rows represent the samples of the genomic spreadsheet, and there is one named column for each observed phenotype (Figure D in S2 File). The Sample Clustering pipeline will only consider categorical and numerical phenotypes. More information about the formatting of these input files can be found at our data preparation resource [https://github.com/KnowEnG/quickstart-demos/blob/master/pipeline_readmes/README-DataPrep.md].

User Parameters

There are a number of parameters that the user must select to run the Sample Clustering Pipeline.

Global Parameters

All modes of Sample Clustering require the user to select the

- [number_clusters] to group the samples into and specify

If consensus clustering is desired, the user must also set the parameters for

- [number_bootstraps] - an integer value
- [bootstrap_percentage] - percent of samples to randomly select in each bootstrap

Knowledge-Guided Only Parameters

In the prior knowledge guided mode, the user must specify the

- [species] - out of the twenty species in the Knowledge Network of the gene features
- [interaction_network] - the gene-gene network available in the Knowledge Network for that species to use in the transformation of each sample's features
- [network_percentage] - controls the extent of influence the interaction network has in the transformation

Standard Clustering Only Parameters

In the standard clustering mode, the user must specify the

- [clustering_algorithm] - currently supports k-means or hierarchical clustering

If hierarchical clustering is selected, the user must also supply the

- [affinity_metric] - currently supports Euclidean, Manhattan, and Jaccard distances
- [linkage_criteria] - currently supports clustering assignments using average, complete, and Ward linkages

Data Preprocessing

Once the user selects the Sample Clustering inputs and parameters, a simple preprocessing step occurs before the main algorithm. First, columns with missing values and row features with duplicate names in the omics spreadsheet are dropped from the analysis. If the user supplied a phenotype spreadsheet, then there is a quick check that the sample names in the "omics" and phenotypic spreadsheets overlap, otherwise an error is returned.

Knowledge-Guided Only

In the knowledge-guided mode, the numeric values of the spreadsheet must be non-negative. The input gene names and identifiers are first mapped to stable Ensembl identifiers of the appropriate [species] using the KN_Mapper tool (see Appendix E in S1 File) and the Redis database of gene aliases that accompanies the current Knowledge Network build. Unmapped rows (either missing or ambiguous mappings) are dropped along with the rows that contain a duplicated mapped gene identifier. Finally in this mode, the gene identifiers of the spreadsheet are compared to the gene identifiers of the selected [interaction_network]. If there is no intersection between these identifier lists, then the algorithm is terminated.

Description of Algorithm

Standard Sample Clustering

Depending on the [clustering_algorithm], the standard clustering mode of the KnowEnG Sample Clustering pipeline uses the sklearn implementations of k-means [http://scikit-learn.org/stable/modules/generated/sklearn.cluster.KMeans.html] and hierarchical clustering [http://scikit-learn.org/stable/modules/generated/sklearn.cluster.AgglomerativeClustering.html] at its core. These methods are run with the default settings and the user supplied [number_clusters], [affinity_metric], and [linkage_criteria]. If bootstrap consensus clustering is not used, then this core clustering result is returned as the final clustering assignments. If [number_bootstraps] is greater than one, then our consensus clustering implementation creates that many copies of the omics spreadsheet, each with only [bootstrap_percentage] of the samples, and the core clustering algorithm is repeated on each. The consensus matrix is built by counting the number of times each pair of samples occurred in each bootstrap clustering. Finally, the final cluster assignments are found by one last application of sklearn's k-means on the consensus matrix.

Random Walks with Restart

Many of the KnowEnG knowledge-guided analysis methods rely on the basic guilt-by-association method known as Random Walk with Restart (RWR). This method belongs to a family of algorithms that model how information spreads within a graph or network that includes the personalized PageRank algorithm [10]. The idea is that information on the nodes of a graph start in a seed state. Node values are updated using some percentage of information that is allowed to propagate across the network neighbors and the remaining percentage from the original seed state. For well-connected, aperiodic networks, this update procedure can be repeated and will converge to a stationary node state where the values on the nodes no longer change significantly. These final stationary node states represent a blending of the original seed and the network information.

In KnowEnG, any [interaction_network] from the Knowledge Network can be used in the RWR algorithm. The weighted interaction network is treated as undirected and converted to a transition probability matrix, T, that captures the probability of following a weighted edge from every starting node to every target node in one step in the undirected network. A sample's original gene feature vector is treated as the seed state over all nodes, V_0. The final network-smoothed vector, V_F, is calculated using steps of a random walk with restart where the vector at each iteration, V_i is defined by:

$$V_i = a * T * V_{i-1} + (1-a) * V_0$$

where a is the [network_percentage] user parameter that dictates the amount of influence the network propagation has on the final result. When the smoothed node state vector converges to a set tolerance, the final vector can be substituted as the description of the sample in further machine learning algorithms.

Knowledge-Guided Clustering

The knowledge-guided clustering mode of the Samples Clustering pipeline supports the same single clustering or consensus clustering structure of the standard mode, but replaces the core

clustering method (Figure E in S2 File). This implementation is based off of the algorithm proposed in [9] and a more detailed description can be found there. The first step of this new core clustering method is to transform each sample's original gene feature vector into a network-smoothed feature vector using random walks with restart (RWR). These transformed, non-negative feature vectors are represented in a genes by samples matrix. The clustering of the samples in the knowledge-guided mode applies Non-negative Matrix Factorization (NMF) [11, 12] to approximate the network-smoothed feature matrix, S, as the product of two reduced-dimension matrices, W and H, where S = WH [13]. The two factorization matrices will have reduced dimension size to the [number_clusters], and the largest value across the k components for each sample in the H matrix will determine the cluster that sample is assigned. The implementation of NMF in our code follows the method used by [9], but converges more quickly because we halt iteration once the clustering becomes stable rather than when the change in ||WH - S|| becomes arbitrarily small.

Phenotype Correlation

If a phenotype matrix is supplied, then the final clustering clusters are evaluated for associations with the supplied phenotypes. Each phenotype is classified as categorical or numerical, and the appropriate test statistic and significance is calculated using scipy functions. For numerical phenotypes, we return the significance p-value of a one-way ANOVA [https://docs.scipy.org/doc/scipy-0.18.1/reference/generated/scipy.stats.f_oneway.html] that tests the null hypothesis that all clusters have the same population mean. For categorical phenotypes, we build the observed contingency table and return the significance p-value of a chi-squared test [https://docs.scipy.org/doc/scipy-0.15.1/reference/generated/scipy.stats.chi2_contingency.html] for the null hypothesis that the categories and the clusters are independent.

Pipeline Outputs

KnowEnG Platform Interface

Running the KnowEnG Sample Clustering pipeline in the KnowEnG Platform will produce results that can be interactively viewed using our Cluster visualization tool. This tool includes the same functionality as the Spreadsheet Visualizer (see Appendix G in S1 File), with the addition of default settings specific to clustering. The original ”omics” spreadsheet is shown as a heatmap, and the columns are grouped by their cluster assignment and sorted by their similarity to the cluster mean (silhouette scores). The omics row features are sorted by the degree of relevance to the cluster assignments. The user can resort these by feature variance. If multiple bootstraps were performed, the consensus matrix is shown below the omics data. Here the matrix shows samples by samples with each cell representing the consensus score. Single-row heatmaps showing phenotypes and/or specific data features can be displayed in alignment with the other visuals and by default include a score indicating correlation to the cluster groupings. These scores can be recalculated for other groupings on the fly. Many additional features can be used to study the data distributions and the relationships between the clustering, the sample phenotypes, and the “omics” features. Survival curves can be generated for each cluster as well as any categorical phenotype, if this serial time/event information is included in the data.

Downloadable Files

The primary downloadable files of the Sample Clustering pipeline are the sample cluster assignments and the consensus matrix that is constructed when multiple bootstraps were performed. There are also silhouette scores provided to indicate cluster quality as well as any phenotype associations if a phenotypic spreadsheet was provided. The cluster centers are reported as a separate output, as well as the top 100 highest scoring gene features for each, which can be passed directly to Gene Set Characterization. *In knowledge-guided mode, additional files containing metadata about the [interaction_network], data preprocessing, and pipeline run are also provided.* More information about the outputs of the pipeline and their structure can be found at [https://github.com/KnowEnG/quickstart-demos/blob/master/pipeline_readmes/README-SC.md].

Methods

We applied the Standard Clustering mode of the KnowEnG Sample Clustering pipeline to the spreadsheet of somatic mutation data from the TCGA PANCAN12 study (see Appendix B of S2 File). We ran [clustering_algorithm] hierarchical clustering several times using different combinations of [affinity_metrics] and [linkage_criteria] without consensus clustering. All runs were performed with [number_clusters] set to 14, the number of clusters returned in the somatic mutation analysis of the original TCGA publication [6]. The Docker image tags at the time of the analysis were knowengdev/data_cleanup_pipeline:10_12_2017 and knowengdev/general_clustering_pipeline:04_22_2018. The results of these runs are summarized in panel A of Table E in S2 Data and Figure F in S2 File. The clinical data that accompany the TCGA data were also uploaded in the analysis, and the Kaplan-Meier plots and p-values were calculated on the "_OS" and "_OS_IND" fields with Spreadsheet Visualizer. The best standard clustering result was with Jaccard affinity and average linkage, which we call "sc_noNet".

We then applied the knowledge-guided mode of the KnowEnG Sample Clustering pipeline to the same genomic and phenotypic spreadsheets. This analysis was performed twice with two different [interaction_networks]. The first network, "HumanNet Integrated Network" [https://s3.amazonaws.com/KnowNets/KN-20rep-1706/userKN-20rep-1706/Gene/9606/hn_IntNet/9606.hn_IntNet.edge], was the 469,784 edge, 15,999 node integrated network from the HumanNet project [2] built from their method for combining edge types. The second network, "STRING Text Mining from Abstracts" [https://s3.amazonaws.com/KnowNets/KN-20rep-1706/userKN-20rep-1706/Gene/9606/STRING_textmining/9606.STRING_textmining.edge], was the 8,511,457 edge, 18,146 node network from the STRINGdb project [3] that aggregates gene co-occurrence across species in literature abstracts. Both knowledge-guided runs were performed without consensus clustering, with 14 [number_clusters] as in the original paper, and with the [network_percentage] fixed at 50%. The Docker image available at the time of the run was knowengdev/samples_clustering_pipeline:04_09_2018. Of the original 39,674 features, 8,483 were removed due to ambiguous/incomplete name mapping and/or duplication. The results of these runs and the Kaplan-Meier p-values from the Spreadsheet Visualizer are summarized in

panel B of Table E in S2 Data and listed in Table F in S2 Data. Plots for these runs are found in Fig 3B and Figure G in S2 File.

We next examined the consistency of our best standard clustering result, “sc_noNet”, our two network based clusterings, “sc_hnInt” and “sc_sText”, and the three prior clusterings/groupings of the samples from the original TCGA paper [6]. These baseline groupings were extracted from the clinical data: 1) “disease” from their disease annotation (“_primary_disease”), “tcga_mut” from their pathway guided clustering (“_PANCAN_mutation_PANCAN”), and “tcga_coca” from their Cluster-Of-Cluster-Assignments clustering (“_PANCAN_Cluster_Cluster_PANCAN”) (panel C of Table E in S2 Data and Table F in S2 Data, and Figure H in S2 File). We calculated the similarity between every pair of clusterings with the adjusted Rand Index score [http://scikit-learn.org/stable/modules/generated/sklearn.metrics.adjusted_rand_score.html] and report the results in Fig 3C. We also compared the clusters of our best network-informed clustering, “sc_hnInt” to the previously discovered clusters from the TCGA paper using the same one-sided Fisher’s exact test available through the KnowEnG implementation of the standard Gene Set Characterization pipeline. The negative log10 p-values of enrichment between the pairs of clusters are shown in Table G in S2 Data.

Finally, we performed the Standard Clustering mode of the KnowEnG Sample Clustering pipeline to each of the four remaining data types (expr, methyl, cnv, and RPPA) for which we had omics spreadsheets (see Appendix B in S2 File and Table A in S2 Data). We ran [clustering_algorithm] hierarchical clustering using ‘euclidean’ [affinity_metrics] and ‘Ward’ [linkage_criteria] without consensus clustering. All runs were performed with the number of clusters found in the original paper [6] for the corresponding data type. The results of these runs are summarized in panel D of Table E in S2 Data. These four clusterings are referred to as “gc_cnv”, “gc_expr”, “gc_methyl”, and “gc_RPPA”.

Resources

Sample Clustering Pipeline

KnowEnG Platform Tool [https://platform.knoweng.org/static/#/pipelines/sample_clustering]
Quickstart Guide [https://knoweng.org/wp-content/uploads/2017/08/SC_Quickstart.pdf]
YouTube Tutorial [https://www.youtube.com/watch?v=nAZzN100B3w]
Data Preparation Guidelines [https://github.com/KnowEnG/quickstart-demos/blob/master/pipeline_readmes/README-DataPrep.md]
Downloadable Results Description [https://github.com/KnowEnG/quickstart-demos/blob/master/pipeline_readmes/README-SC.md]

Seven Bridges Cancer Genomics Cloud

Public Tool [https://cgc.sbgenomics.com/public/apps/#mepstein/knoweng-samples-clustering-public/]
Quickstart Guide [https://knoweng.org/wp-content/uploads/2018/07/SC_CGC_Quickstart.pdf]

Docker and GitHub Repositories

Knowledge-Guided Docker [https://hub.docker.com/r/knowengdev/samples_clustering_pipeline/]

Knowledge-Guided GitHub [https://github.com/KnowEnG/Samples_Clustering_Pipeline]
Standard Clustering Docker [https://hub.docker.com/r/knowengdev/general_clustering_pipeline/]
Standard Clustering GitHub [https://github.com/KnowEnG/General_Clustering_Pipeline]
Cluster Evaluation Docker [https://hub.docker.com/r/knowengdev/clustering_evaluation/]
Cluster Evaluation GitHub [https://github.com/KnowEnG/Clustering_Evaluation]
Data Cleanup Docker [https://hub.docker.com/r/knowengdev/data_cleanup_pipeline/]
Data Cleanup GitHub [https://github.com/KnowEnG/Data_Cleanup_Pipeline]
Pipeline Utilities Docker [https://hub.docker.com/r/knowengdev/base_image/]
Pipeline Utilities GitHub [https://github.com/KnowEnG/KnowEnG_Pipelines_Library]

Figures

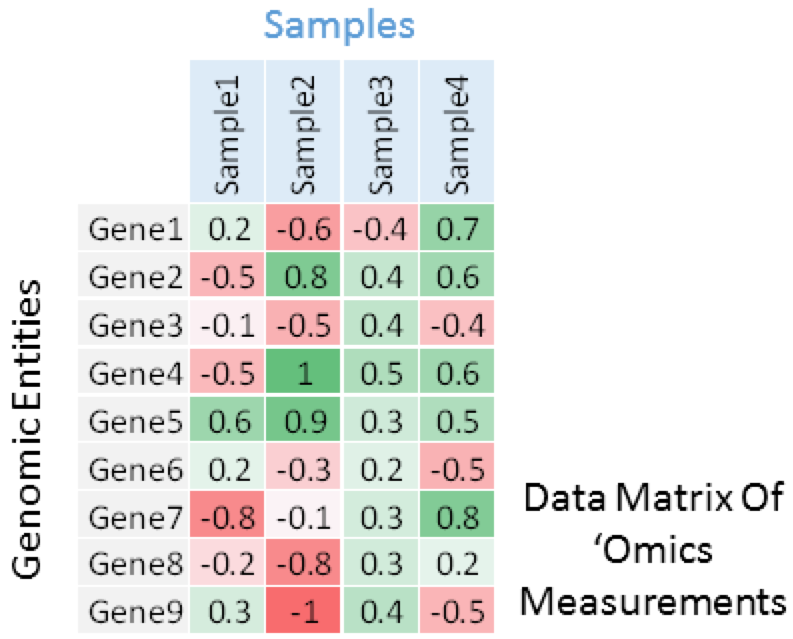

	Sample1	Sample2	Sample3	Sample4
Gene1	0.2	-0.6	-0.4	0.7
Gene2	-0.5	0.8	0.4	0.6
Gene3	-0.1	-0.5	0.4	-0.4
Gene4	-0.5	1	0.5	0.6
Gene5	0.6	0.9	0.3	0.5
Gene6	0.2	-0.3	0.2	-0.5
Gene7	-0.8	-0.1	0.3	0.8
Gene8	-0.2	-0.8	0.3	0.2
Gene9	0.3	-1	0.4	-0.5

Figure C. Sample of "omics" Spreadsheet.
Users must input genomic spreadsheets were the genes features are the rows and the samples are the columns.

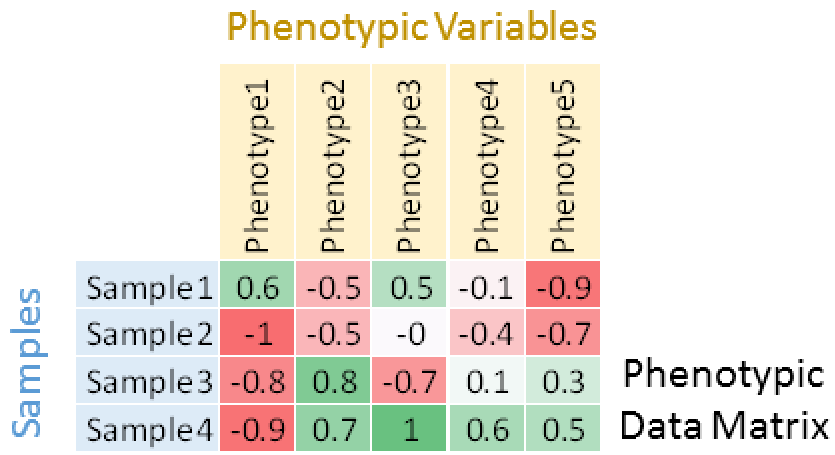

Figure D. Sample of Phenotypic Spreadsheet.
Users must input phenotypic spreadsheets were the samples are the rows and each column represent a distinct phenotype.

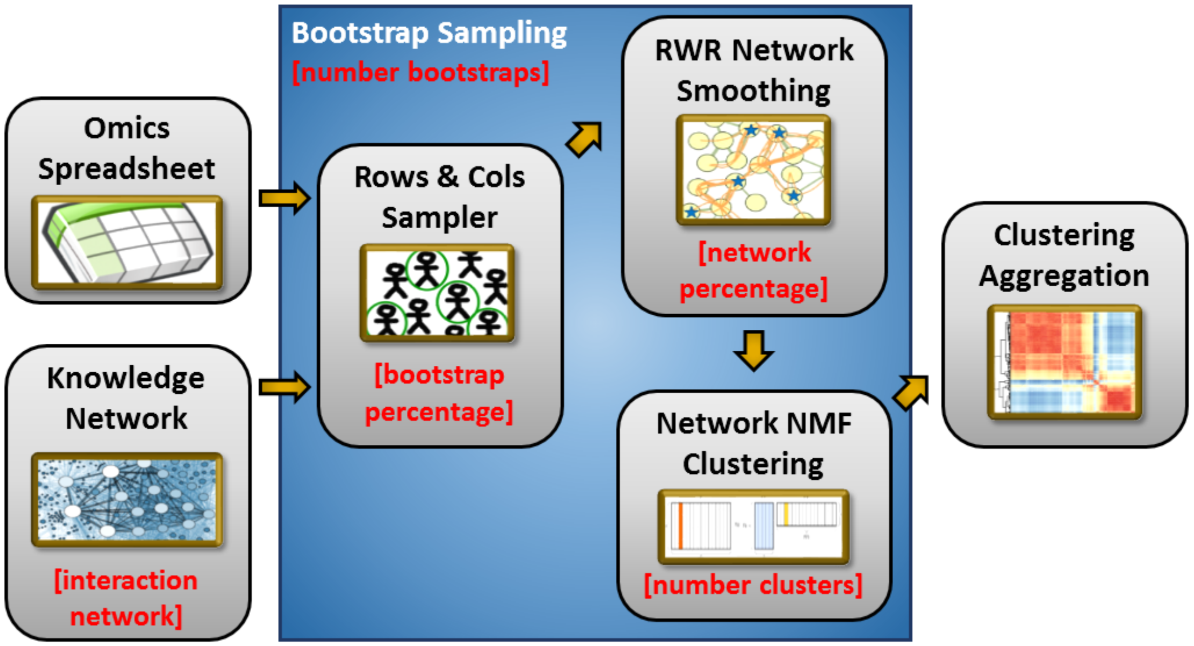

Figure E. Overview of Knowledge-Guided Sample Clustering.
For each bootstrap, the core clustering algorithm smooths the original features using the knowledge network and then performs NMF clustering. The consensus matrix is built from the bootstraps and finally clustered with K-means. Steps are shown with their corresponding user parameters (in red).

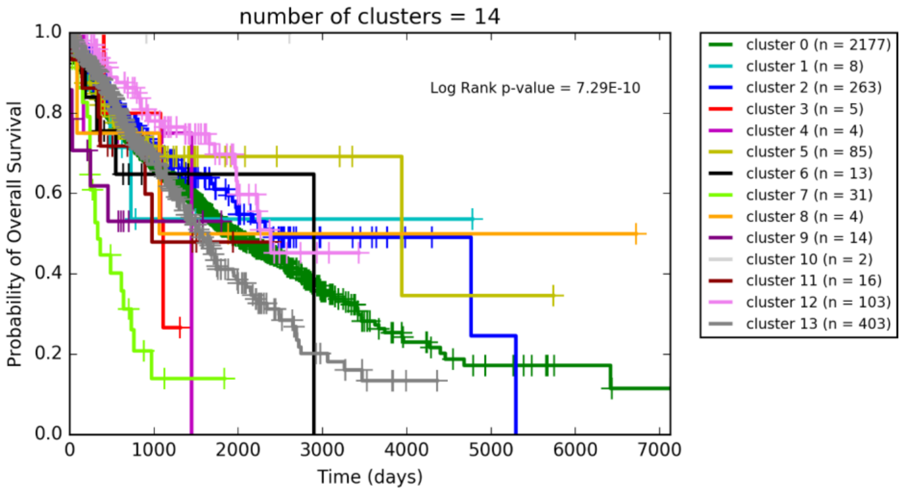

Figure F. Survival Analysis of “sc_noNet” Clustering.
Each cluster is plotted as a separate survival curve in the Kaplan-Meier plot and the p-value of the multivariate log rank test is reported.

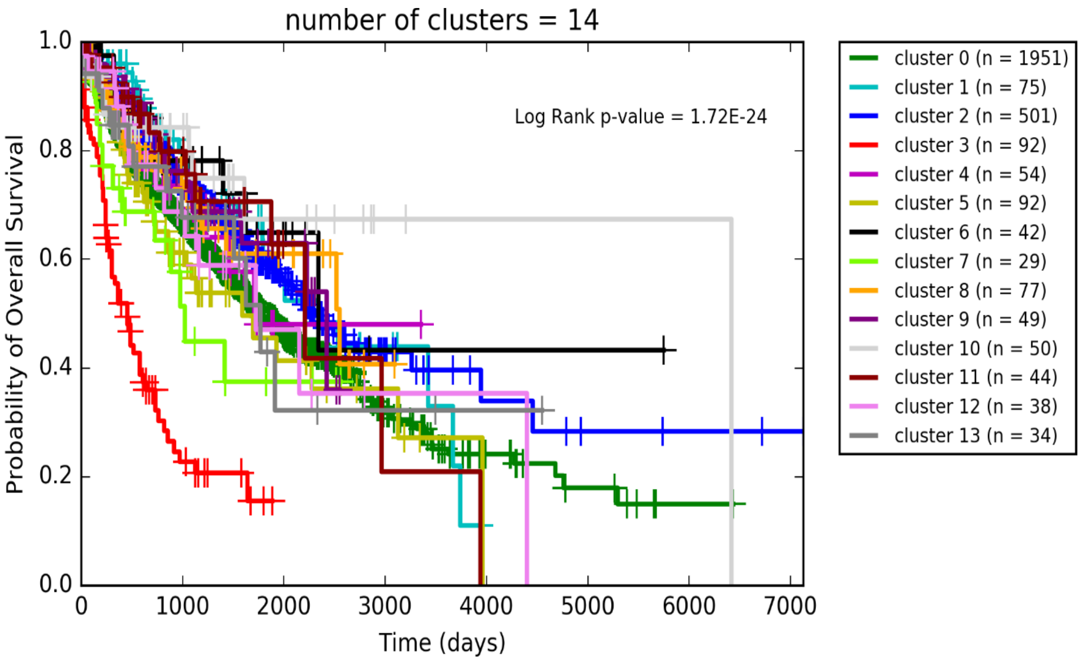

Figure G. Survival Analysis of “sc_sText” Clustering.
Each cluster is plotted as a separate survival curve in the Kaplan-Meier plot and the p-value of the multivariate log rank test is reported.

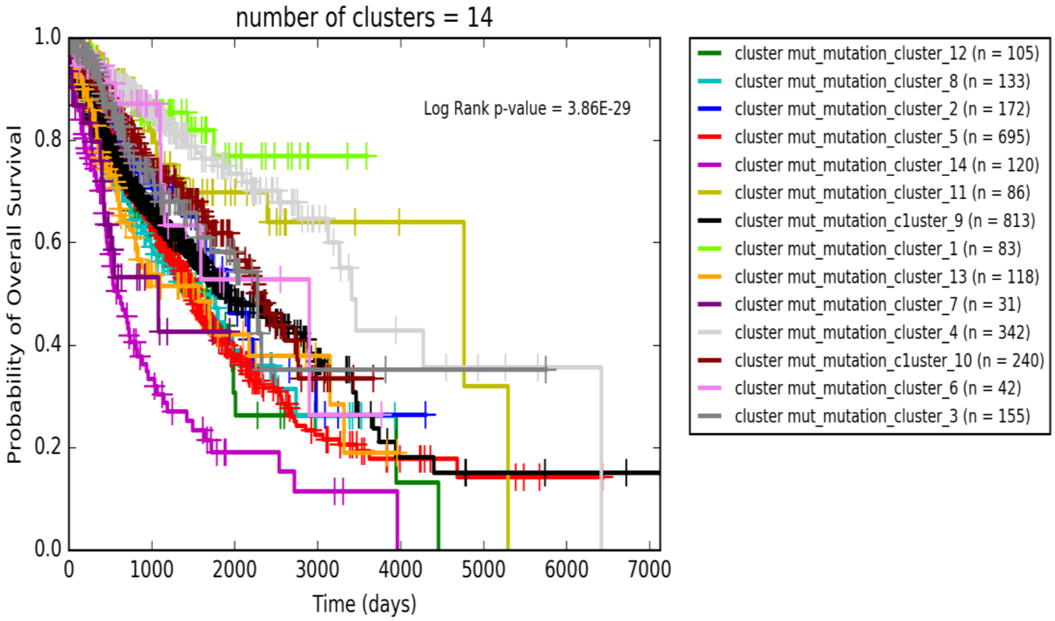

Figure H. Survival Analysis of "tcga_mut" Clustering.
Each cluster is plotted as a separate survival curve in the Kaplan-Meier plot and the p-value of the multivariate log rank test is reported.

Tables

Table E. PANCAN12 Mutation Sample Clusterings.

Shows statistics for each clustering of the mutation samples. The first three columns indicate the tool used to perform the clustering as well as additional details about the parameter settings. The next three columns indicate the number of clusters created as well as the size and percentage of the largest cluster. Finally, the last column shows the Kaplan-Meier p-value of the significance of the relationship between the clustering and survival outcome. Separated into sections by A) standard Sample Clustering on mutation data, B) knowledge-guided Sample Clustering on mutation data, C) original TCGA clusterings of samples, D) standard Clustering on alternative data types, and E) COCA analysis with Sample Clustering Pipeline.

Table F. PANCAN12 Mutation Samples Cluster Assignments.

For each PANCAN12 Mutation Sample in the omics spreadsheet (row), shows the cluster assignment for the six selected clusterings: "sc_noNet" - best hierarchical clustering, "sc_hnInt" and "sc_sText" - network-guided clusterings with HumanNet Integrated and STRING TextMining networks respectively, "disease" - grouping by TCGA primary disease, and "tcga_mut" and "tcga_coca", mutation-only and COCA clustering analysis in original TCGA paper.

Table G. Enrichment between Clusters.

For a cluster from our best network-guided method clusterings, "sc_hnInt", and from one of three original TCGA clusterings, shows the negative log10 p-value of the one-sided Fisher's

exact test for enrichment. The original clusterings are “disease” - grouping by TCGA primary disease, and “tcga_mut” and “tcga_coca”, mutation-only and COCA clustering analysis in TCGA PANCAN12 paper.

Appendix D: Characterization of Knowledge Guided Mutation Subtypes

Background

In our first case study, we presented the clustering of pan-cancer patient mutation profiles using the network based stratification (NBS) [9] method (see Appendix C in S2 File) and found that this yields better clustering of mutation data, with more size-balanced clusters and with survival analysis yielding stronger separations. We also noted that these clusters do not simply recapitulate the tumor types of patients, suggesting that the discovered clusters may reveal new structures in the data.

To explore this further and determine the molecular and functional characteristics of these clusters, if any, we identified the genes and pathways whose mutation status/counts are most discriminative of each cluster. We began by extracting the genes by patient matrix that had been subjected to clustering whose columns of mutation data have been transformed with into network-smoothed feature vectors using random walks with restart (RWR). For each of the 14 clusters in our ‘hnInt’ knowledge-guided clustering using the HumanNet Integrated network [2], we performed t-tests (using KnowEnG’s standard Gene Prioritization from Appendix A in S3 File) to identify the top 100 genes differentially mutated between the given cluster and all other clusters. We then performed apply the Fisher’s exact test to this gene sets with our Gene Set Characterization pipeline (see Appendix C in S4 File) to identify Gene Ontology [4], Reactome [14], Pathway Commons, and NCI pathways (downloaded from Enrichr [5]) enriched with these 100 genes. We repeated this for all 14 clusters separately.

Results

Table H in S2 Data shows basic information about the discovered clusters (rows) — their sizes, and how many patients of each cluster belongs to which tumor type. We note that clusters vary in size, between 3 and 993, with mean, median, and standard deviation of sizes being 234, 60, and 324 respectively. We also note that most clusters are mixed in terms of tumor types, but some are more dominated by a few tumor types, e.g., c2 is almost entirely AML, c3 is dominated by lung cancers (LUAD, LUSC), c4 is mostly breast and uterine. The two largest clusters are c0 (enriched in ovarian cancers) and c1 (enriched in kidney cancers).

Table I in S2 Data and Table J in S2 Data show genes that are most discriminative of each cluster and their enriched properties respectively. For example,

A. c0 and c1, the largest clusters, are enriched for general tumorigenic properties and pathways related to p53 and apoptosis.

B. Among the several genes whose mutation status is uniquely discriminative of c0 was SOCS1, an important tumor suppressor that attenuates cytokine signaling [15]. This is particularly interesting because the two most over-represented tumor types in c0 are ovarian and breast cancers, where prolactin and IL-6 secretion from the ovary and mammary gland induce growth by stimulating JAK/STAT signaling. The third largest cancer type in cluster c0 is head & neck cancer, where a high level of JAK/STAT signaling is also known to be important. Cluster c0 is also discriminated from other clusters by ARID1A mutation status. ARID1A is an important SWI/SNF tumor suppressor [16].
C. Cluster c1, which is highly overrepresented in kidney renal clear cell carcinoma, was uniquely marked by S100A16, S100A6, S100A5, and S100A3, among other genes. The finding of S100A as a marker of this cluster is potentially significant, given its tumor type composition (kidney and breast) [17].
D. Cluster c2 is marked by mutation status of genes in the HIF-1-alpha and HIPK20-related pathways, which is consistent with its AML-dominated composition [18].
E. Cluster c4 includes mainly breast and uterine carcinoma patients, and is associated with PIP3, PI3K/AKT and GAB1-related pathways, in agreement with prior knowledge about those tumor types [19].
F. Cluster c5 is associated with titin binding, muscle-related pathways.
G. Cluster c7 is associated with sarcoplasmic reticulum.
H. Cluster c8 is associated with protein autophosphorylation.
I. Cluster c9 is associated with neuronal action potential, KEAP1-NFE2L2.
J. Cluster c10 is associated with histone methyltransferase activity (H3-K36 specific).

In other words, the discovered clusters are characterized by mutations in genes from specific and distinct pathways, even when they are mixed in terms of tumor type representation.

We also paid special attention to the characteristics of the two largest clusters c0 and c1, both of which include a number of breast cancer patients but also include a large number of ovarian and kidney cancer patients respectively. In particular, we examined genes whose mutation status is discriminative between these clusters, and found that the genes marking cluster c1 are strongly enriched in calcium ion binding.

Tables

Table H. 'hnInt' Clusters by Disease Type.

For each of the 14 clusters (rows) from our knowledge guided clustering of the somatic mutation data using the HumanNet Integrated network, we show the number of samples from each tumor disease type (columns).

Table I. 'hnInt' Cluster Specific Genes.

For each of the 14 clusters from our knowledge guided clustering using the HumanNet Integrated network, we found the set of top 100 genes (rows) according to the a t-test on the network smoothed mutation values between the samples within each cluster (columns) vs all others. Membership in each of these genes sets is indicated with a table value of 1.

Table J. Top Pathways Enriched in Genes Specific for 'hnInt' Clusters.
For each top 100 cluster specific gene list from our 'hnInt' clustering, we return the top 5 enriched pathways using the one-sided Fisher's exact test (reported as the negative log10 p-value (neglog pval)). The number of genes annotated for these pathways (pathway size) and the source of the information is also reported (collection).

Appendix E: KnowEnG Jupyter Tools and Combining Multi-omics Clusterings

Overview

JupyterHub notebooks provide users with a web-browser interface for Python kernel code running on the cloud server hardware, allowing users to conveniently from a local machine complete big data computation on the server where the data is stored and analysis is executed. Notebooks avoid the need to move data through slow network connections and allow the use of cloud resources to complete data-wrangling tasks. The current collection of KnowEnG notebooks provide the means to perform basic spreadsheet matrix transformations such as transposing, selecting, thresholding and more specific, complex data manipulations without requiring a user to understand programming or transfer the data to a local spreadsheet software.

User Experience

KnowEnG provides a JupyterHub [https://jupyterhub.readthedocs.io/en/stable/] server instance that gives user access to secure notebooks for data manipulation tasks. Login to the JupyterHub server is handled by the open source access management platform CILogon [https://www.cilogon.org/home] that allows users to researchers to use login credentials from their home institutions or other existing research platform.

The JupyterHub server is separate from the KnowEnG platform, so users wishing to switch between the two must currently manually download and upload the files between servers. Inputs and outputs are (.tsv) tab separated value spreadsheet files. Once on the landing page of the Jupyter notebook, users may click on the ‘user_data/’ directory, and then use the file upload button on the upper right of the page to upload spreadsheet files. By clicking on the folder icon, the user can return to the landing page and start the transformation notebook by clicking the Spreadsheets_Transformation.ipynb file. Once all the cells of the notebook are running, selection of input files and data transformations is accomplished by using the labeled controls. An hourglass icon will appear in the figure tab when calculations are running, and when finished the results will appear in the ‘results/’ directory. More detailed instructions and examples can be found are the following resource:
[https://github.com/KnowEnG/Spreadsheets_Transformation/blob/master/docs/notebook_Readme.md].

The containerized versions of the spreadsheet transformations may be run wherever other pipeline containers are run if security requirements allow. The Docker images for the single-user local-server are located at:
[https://hub.docker.com/r/knowengdev/spreadsheets_transformation]. Users wishing to set up their own multi-user JupyterHub instance may use the following Docker image:
[https://hub.docker.com/r/knowengdev/jupyter_notebooks]

Description of Algorithm

The data transformations available in the current version of the KnowEnG Spreadsheets_Transformation.ipynb notebook include:

Category to Binary	convert single spreadsheet column to multiple (one per label) binary columns
Transpose	rotate the matrix such that rows become columns and columns become rows
Intersect	extract the rows in common from two spreadsheets into a new spreadsheet
Merge	combine all the rows and columns in two spreadsheets in a new spreadsheet
Select Rows	extract smaller spreadsheet from a spreadsheet and a list of rows
Average on Labels	process spreadsheet and sample labels to find averages per label
Subset on Label	extract smaller spreadsheet that contains only samples with phenotype label
Misc Transforms	take absolute value, z-transform, log-transform, or threshold of spreadsheet
Descriptive Stats	report min, max, sum, mean, median, stand dev, and variance by row or column

Methods

From the UCSC Cancer Genome Browser PANCAN12 TCGA clinical data column, "_PANCAN_miRNA_PANCAN", we extracted the miRNA clusterings in the original paper [6]. This clusterings was saved as a two column file, the first column with the TCGA sample identifiers and the second with the clinical data column cluster names.

We also downloaded the sample cluster assignment file, “sample_labels_by_cluster.txt”, from five runs of the Sample Clustering pipeline that we performed on the remaining five omics data types. Standard clustering with ’euclidean’ [affinity_metric] and ‘Ward’ [linkage_criteria] was used to produce the clusterings for “gc_methyl”, “gc_expr”, “gc_cnv”, and “gc_RPPA”. Knowledge-guided clustering using the HumanNet Integrated network, “sc_hnInt” (see Table E in S2 Data), was used for gene-level somatic mutation data. More details about these five clusterings can be found in Appendix C in S2 File.

Once we had gathered the files for all six data types, we uploaded them to the “user_data/CLUSTERS/” directory available in the KnowEnG Jupyter notebook hub server and removed the example files. We then started the “notebooks/Spreadsheet_Transformation.ipynb” notebook, ran all cells, and selected the “Category to binary” transformation mini-pipeline selecting “CLUSTERS” and “process directory” as the two parameters. This converted our six clustering files into a single clusters by samples matrix with binary 0/1 values indicating the sample’s membership in the cluster. This output file, “CLUSTERS_categorical_binary.tsv”, was found in the “/results/” directory of the notebook interface. We moved this output to the “user_data/” along with a list of the 3524 samples used in the COCA analysis and reran the

“Select Rows” cell and launched the minipipeline with the two files to remove the unwanted TCGA samples. Then, we moved the resulting output, ”CLUSTERS_categorical_binary_select_rows.tsv”, to “user_data/”, reran the “Transpose” cell, and launched that minipipeline with this file to reorient the final output matrix, "CLUSTERS_categorical_binary_select_rows.tsv". Finally, we downloaded this spreadsheet further analysis in the KnowEnG Platform. We renamed the cluster feature names for clarity, and the resulting table is available in Table K in S2 Data.

Resources

Public Tool [https://knowtebook.knoweng.org/hub/login]
Quickstart for JupyterHub
[https://github.com/KnowEnG/Spreadsheets_Transformation/blob/master/docs/notebook_Readme.md]
Spreadsheet Transformation Docker Repository
[https://hub.docker.com/r/knowengdev/spreadsheets_transformation]
Spreadsheet Transformation GitHub
[https://github.com/KnowEnG/Spreadsheets_Transformation]

Tables

Table K. Combined Multi-omics Clustering Matrix.
A matrix with 0/1 membership indicator values for each PANCAN12 sample (columns) and each different cluster from the original tcga.miRNA clustering, four standard KnowEnG Sample Clusterings of other data types, and one “sc_hnInt” knowledge-guided Sample Clustering on the mutation data.

Appendix F: COCA Analysis of Multi-omics TCGA Data

Methods

We next attempted to recreate the Cluster-Of-Cluster-Assignment (COCA) analysis from the TCGA PANCAN analysis of 12 cancer types [6] using the KnowEnG Platform. To do this, we simply need to submit the matrix we constructed in Appendix E in S2 File to the standard mode of the Sample Clustering pipeline and use the consensus clustering feature. The multi-omics matrix we previously created was a clusters by samples matrices with 0/1 membership indicator values that contained clusters from six different types of omics data. This matrix was a “recreation” of the original’s papers COCA analysis except using the results of the KnowEnG Sample Clustering on each original omics data spreadsheet (except miRNA, which was exactly identical). This “recreation” matrix is saved as Table K in S2 Data.

We submitted the “recreation” multi-omics matrix to the standard mode of Sample Clustering. We asked for hierarchical clustering to produce 13 [number_clusters] using the ‘euclidean’

[affinity_metrics] and the 'ward' [linkage_criteria]. We performed these runs with consensus clustering, with 200 [number_bootstraps] bootstraps, sampling 80% [bootstrap_percentage] of the tumor samples for each one. This is similar to the analysis in the original paper, which found 13 clusters using consensus clustering with hierarchical clustering and 80% sampling percentage. In the original paper, they used Pearson correlation as the affinity metric and ran the method for 1000 bootstrap iterations. The Docker image tags at the time of the analysis were knowengdev/data_cleanup_pipeline:10_12_2017 and knowengdev/general_clustering_pipeline:04_22_2018 and the clustering results are in Table L in S2 Data.

The resulting clustering, "recreation consensus COCA" is of equal quality to the original COCA clusters reported in the TCGA PANCAN analysis [6]. This evaluation was done using the Kaplan-Meier plots (Fig 3E, Figure I in S2 File) and p-values (panel E in Table E in S2 File) calculated from survival analysis on the "_OS" and "_OS_IND" clinical annotation fields. To recreate a figure from the original analysis of [6], our COCA cluster assignments were shown in the Spreadsheet Visualizer (Appendix G in S1 File) along with the mutation and copy number variation data (Table M in S2 Data) of selected genes.

Figures

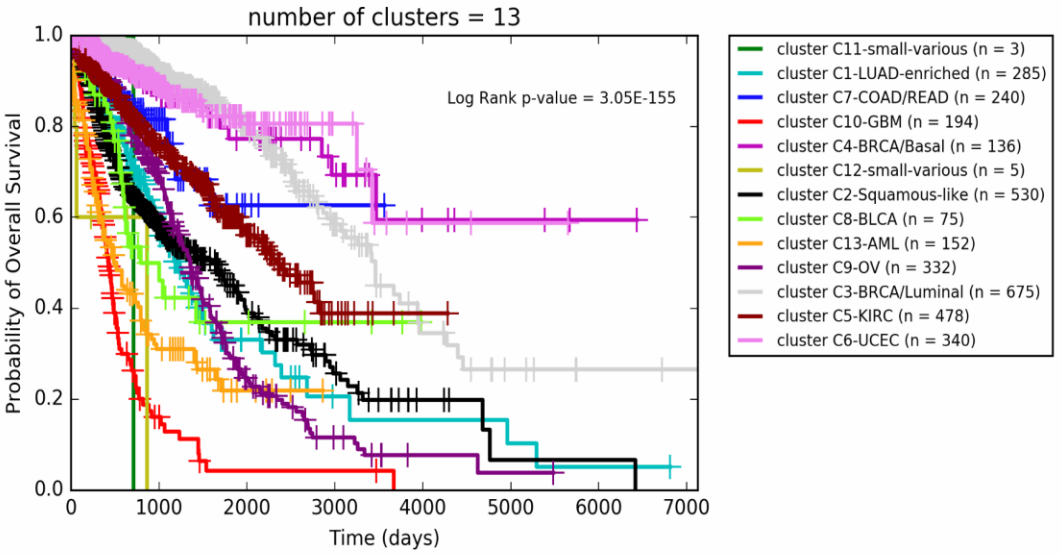

Figure I. Survival Analysis of "tcga_coca" Original COCA .

Each cluster is plotted as a separate survival curve in the Kaplan-Meier plot and the p-value of the multivariate log rank test is reported.

Tables

Table L. Cluster Assignments for COCA Survival Analysis.

Information about the TCGA samples from the COCA analysis including their COCA analysis cluster assignment 'coca_cluster' as well as their overall survival indicator, 'OS_IND', and number of days, 'OS_Days', taken from the TCGA clinical information.

Table M. Selected Gene Copy Number Variation Data.
For nine selected genes, shows the gene copy number score for all TCGA samples from the TCGA_PANCAN12_genecopynumber-2015-01-28 dataset.

Appendix G: Sample Clustering on METABRIC Transcriptomic Dataset

Methods

Data Collection

Following [20], we obtained a list of 253 genes that have been previously shown to be involved in the epithelial to mesenchymal transition (EMT) from [21]. In addition, we downloaded the transcriptomic and clinical data corresponding to the METABRIC study [22] from OASIS [http://oasis-genomics.org/] and the supplemental material of the original paper. We focused our analysis on the 1058 samples with gene expression and overall survival information. In all analysis, the log2 transformed and Z normalized probe intensities were used. Figure J in S2 File shows the Kaplan-Meier survival analysis of different PAM50 subtypes in this dataset.

Clustering of samples based on the EMT signature

We used standard mode of the Sample Clustering pipeline in KnowEnG to cluster samples above into two groups using only the expression of the EMT genes (henceforth the EMT signature). We ran [clustering_algorithm] hierarchical clustering using [affinity_metrics] Euclidean and [linkage_criteria] of 'average', 'complete', and 'Ward' without consensus clustering. The Docker image tags at the time of the analysis were knowengdev/data_cleanup_pipeline:01_04_2018 and knowengdev/general_clustering_pipeline:04_22_2018. Of the three clusterings obtained, two (obtained using average and complete linkage) were significantly biased: one cluster contained only one sample and the other cluster contained all other samples. Hence, we focused on results obtained using the 'Ward' linkage that provided less biased results. The Kaplan-Meier survival analysis did not show a significant distinction between the survival of the two clusters ($p = 0.12$, log-rank test, Figure K in S2 File), confirming the results of [20].

Next, we sought to determine whether the knowledge-guided Sample Clustering of KnowEnG could improve these results. Since this pipeline only accepts non-negative "omic" input datasets, we obtained the absolute value of the (normalized) EMT gene expression profiles. Intuitively, a value close to zero means that the gene is expressed close to the mean, while a large positive value shows that the gene is highly or lowly expressed compared to other genes (i.e. the gene is differentially expressed). We ran the knowledge-guided Sample Clustering using integrated HumanNet (hnInt) (Figure L in S2 File) and STRING TextMining (sText) (Figure M in S2 File). In both cases, the p-value of Kaplan-Meier log rank test was significant: $p = 7.56E-4$ for hnInt and p

= 3.08E-4 for sText, showing significant improvement compared to the standard mode of clustering.

Figures

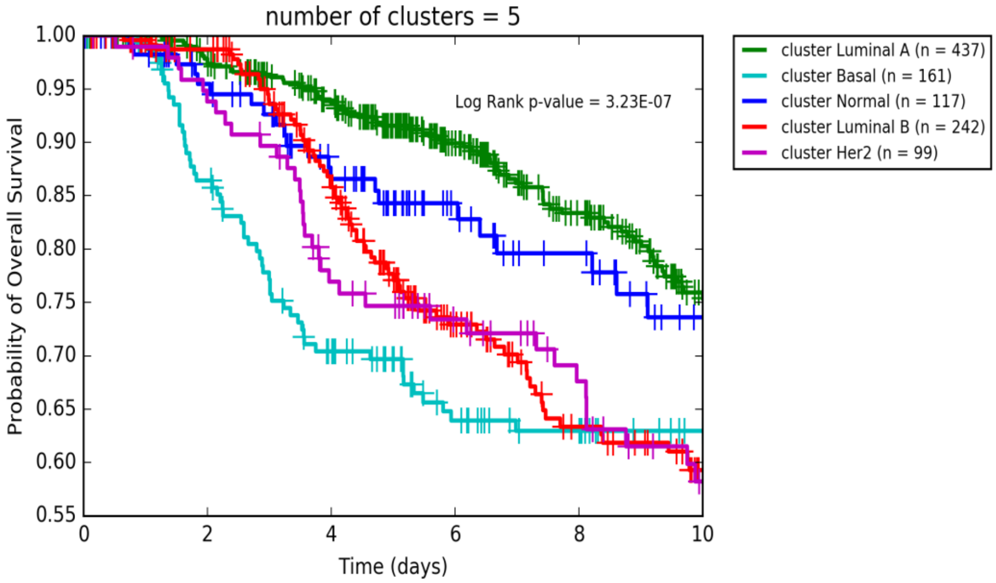

Figure J. Survival probabilities of PAM50 subtypes of METABRIC samples.
Each cluster is plotted as a separate survival curve in the Kaplan-Meier plot and the p-value of the multivariate log rank test is reported.

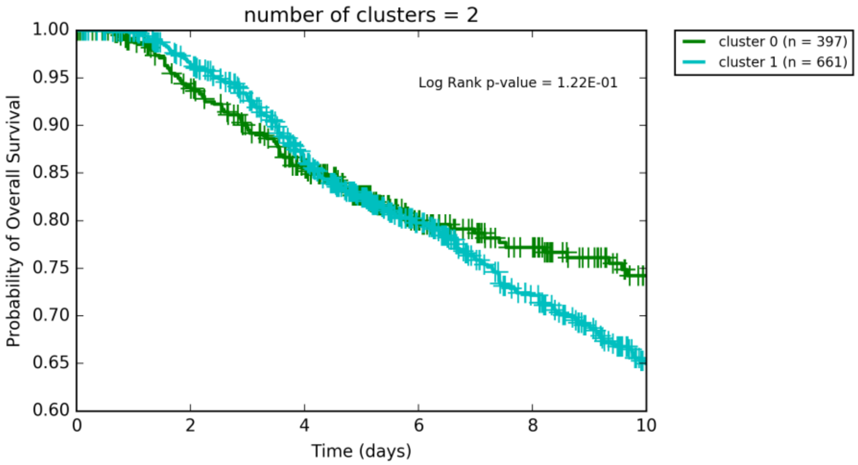

Figure K. Survival Analysis using EMT signature and Standard Sample Clustering.
Each cluster is plotted as a separate survival curve in the Kaplan-Meier plot and the p-value of the log rank test is reported.

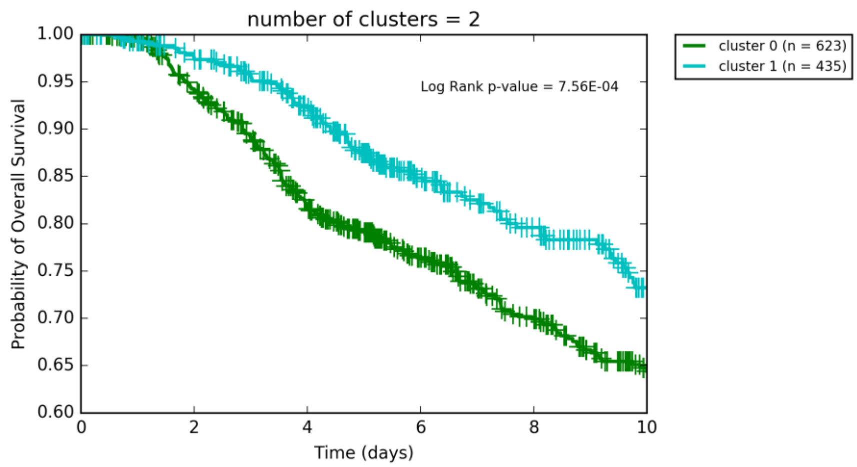

Figure L. Survival Analysis using EMT signature and Sample Clustering with hnInt.

Each cluster is plotted as a separate survival curve in the Kaplan-Meier plot and the p-value of the log rank test is reported.

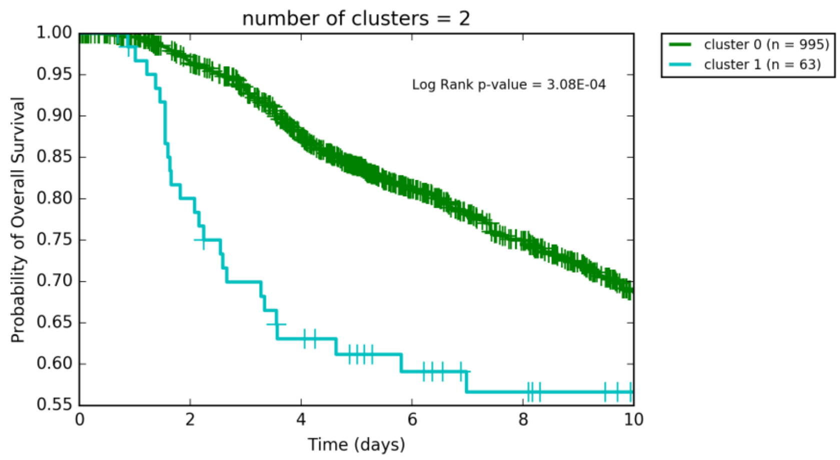

Figure M. Survival Analysis using EMT signature and Sample Clustering with sText.

Each cluster is plotted as a separate survival curve in the Kaplan-Meier plot and the p-value of the log rank test is reported.

References

1. Zerbino DR, Achuthan P, Akanni W, Amode MR, Barrell D, Bhai J, et al. Ensembl 2018. Nucleic Acids Res. 2018;46(D1):D754-D61. doi: 10.1093/nar/gkx1098. PubMed PMID: 29155950; PubMed Central PMCID: PMCPMC5753206.
2. Lee I, Blom UM, Wang PI, Shim JE, Marcotte EM. Prioritizing candidate disease genes by network-based boosting of genome-wide association data. Genome Res. 2011;21(7):1109-21. doi: 10.1101/gr.118992.110. PubMed PMID: 21536720; PubMed Central PMCID: PMCPMC3129253.
3. Szklarczyk D, Franceschini A, Wyder S, Forslund K, Heller D, Huerta-Cepas J, et al. STRING v10: protein-protein interaction networks, integrated over the tree of life. Nucleic Acids Res. 2015;43(Database issue):D447-52. doi: 10.1093/nar/gku1003. PubMed PMID: 25352553; PubMed Central PMCID: PMCPMC4383874.
4. Ashburner M, Ball CA, Blake JA, Botstein D, Butler H, Cherry JM, et al. Gene ontology: tool for the unification of biology. The Gene Ontology Consortium. Nat Genet. 2000;25(1):25-9. doi: 10.1038/75556. PubMed PMID: 10802651; PubMed Central PMCID: PMCPMC3037419.
5. Chen EY, Tan CM, Kou Y, Duan Q, Wang Z, Meirelles GV, et al. Enrichr: interactive and collaborative HTML5 gene list enrichment analysis tool. BMC Bioinformatics. 2013;14:128. doi: 10.1186/1471-2105-14-128. PubMed PMID: 23586463; PubMed Central PMCID: PMCPMC3637064.
6. Hoadley KA, Yau C, Wolf DM, Cherniack AD, Tamborero D, Ng S, et al. Multiplatform analysis of 12 cancer types reveals molecular classification within and across tissues of origin. Cell. 2014;158(4):929-44. doi: 10.1016/j.cell.2014.06.049. PubMed PMID: 25109877; PubMed Central PMCID: PMCPMC4152462.
7. Goldman M, Craft B, Swatloski T, Cline M, Morozova O, Diekhans M, et al. The UCSC Cancer Genomics Browser: update 2015. Nucleic Acids Res. 2015;43(Database issue):D812-7. doi: 10.1093/nar/gku1073. PubMed PMID: 25392408; PubMed Central PMCID: PMCPMC4383911.
8. Monti S, Tamayo P, Mesirov J, Golub T. Consensus clustering: a resampling-based method for class discovery and visualization of gene expression microarray data. Machine learning. 2003;52(1-2):91-118.
9. Hofree M, Shen JP, Carter H, Gross A, Ideker T. Network-based stratification of tumor mutations. Nat Methods. 2013;10(11):1108-15. doi: 10.1038/nmeth.2651. PubMed PMID: 24037242; PubMed Central PMCID: PMCPMC3866081.
10. Page L, Brin S, Motwani R, Winograd T. The PageRank citation ranking: Bringing order to the web. Stanford InfoLab, 1999.
11. Lee DD, Seung HS. Learning the parts of objects by non-negative matrix factorization. Nature. 1999;401(6755):788.
12. Li Y, Ngom A. A new Kernel non-negative matrix factorization and its application in microarray data analysis. CIBCB. 2012;371:378.
13. Cai D, He X, Wu X, Han J, editors. Non-negative matrix factorization on manifold. Data Mining, 2008 ICDM'08 Eighth IEEE International Conference on; 2008: IEEE.
14. Fabregat A, Jupe S, Matthews L, Sidiropoulos K, Gillespie M, Garapati P, et al. The Reactome Pathway Knowledgebase. Nucleic Acids Res. 2018;46(D1):D649-D55. doi: 10.1093/nar/gkx1132. PubMed PMID: 29145629; PubMed Central PMCID: PMCPMC5753187.
15. Sutherland KD, Lindeman GJ, Choong DY, Wittlin S, Brentzell L, Phillips W, et al. Differential hypermethylation of SOCS genes in ovarian and breast carcinomas. Oncogene. 2004;23(46):7726.
16. Guan B, Wang T-L, Shih I-M. ARID1A, a factor that promotes formation of SWI/SNF-mediated chromatin remodeling, is a tumor suppressor in gynecologic cancers. Cancer research. 2011;71(21):6718-27.
17. Takashi M, Haimoto H, Murase T, Mitsuya H, Kato K. An immunochemical and immunohistochemical study of S100 protein in renal cell carcinoma. Cancer. 1988;61(5):889-95.
18. Velasco-Hernandez T, Hyrenius-Wittsten A, Rehn M, Bryder D, Cammenga J. HIF-1α can act as a tumor suppressor gene in murine acute myeloid leukemia. Blood. 2014;124(24):3597-607.
19. Myers AP. New strategies in endometrial cancer: targeting the PI3K/mTOR pathway—the devil is in the details. Clinical Cancer Research. 2013;19(19):5264-74.
20. Emad A, Ray T, Jensen TW, Parat M, Natrajan R, Sinha S, et al. An epithelial-mesenchymal-amoeboid transition gene signature reveals molecular subtypes of breast cancer progression and metastasis. bioRxiv. 2017:219410. doi: 10.1101/219410.

21. Taube JH, Herschkowitz JI, Komurov K, Zhou AY, Gupta S, Yang J, et al. Core epithelial-to-mesenchymal transition interactome gene-expression signature is associated with claudin-low and metaplastic breast cancer subtypes. Proc Natl Acad Sci U S A. 2010;107(35):15449-54. doi: 10.1073/pnas.1004900107. PubMed PMID: 20713713; PubMed Central PMCID: PMCPMC2932589.
22. Curtis C, Shah SP, Chin SF, Turashvili G, Rueda OM, Dunning MJ, et al. The genomic and transcriptomic architecture of 2,000 breast tumours reveals novel subgroups. Nature. 2012;486(7403):346-52. doi: 10.1038/nature10983. PubMed PMID: 22522925; PubMed Central PMCID: PMCPMC3440846.
