# Supplementary material for: Knowledge-guided analysis of "omics" data using the KnowEnG cloud platform
Source: PLoS Biol. 2020 Jan 23;18(1):e3000583. doi: 10.1371/journal.pbio.3000583 (PMC6977717; doi:10.1371/journal.pbio.3000583)
Supplement: S3 File — Three appendices describing the pipelines, methods, and additional analyses that relate to the second case study of performing gene prioritization with TCGA transcriptomic data. TCGA, the Cancer Genome Atlas. (PDF) [file pbio.3000583.s003.pdf]

S3 File: Methods for Gene Prioritization Case Study

Table of Contents

Table of Contents **1**
Appendix A: Feature Prioritization on TCGA PANCAN12 Transcriptomic Datasets **3**
Overview 3
User Inputs 3
User Parameters 4
Preprocessing of the data 5
Description of Algorithm 5
Pipeline Outputs 6
Methods 7
Resources 7
Tables 8
Table A. Top 100 genes without using the Knowledge Network. 8
Table B. Top 100 genes using HumanNet Integrated Network. 8
Table C. Top 100 genes using STRING Text Mining. 8
Table D. Cross-dataset Intersection. 8
Table E. Intersections of Top Gene Results using No Network Knowledge. 9
Table F. Intersections of Top Gene Results using HumanNet Integrated. 9
Table G. Intersections of Top Gene Results using STRING Text Mining. 9
Appendix B: Characterization of Top Genes Identified for Each Tumor Type **9**
Methods 9
Figures 11
Figure A. IntOGen Cancer-Specific Drivers Identified using noNet and hnInt. 11
Figure B. IntOGen Cancer-Specific Drivers Identified using noNet and sText. 12
Figure C. IntOGen Driver Genes among Top Genes Identified using noNet and hnInt. 12
Figure D. IntOGen Driver Genes among Top Genes Identified using noNet and sText. 13
Figure E. COSMIC Driver Genes among Top Genes identified Using noNet and hnInt. 13
Figure F. COSMIC Driver Genes among Top Genes identified Using noNet and sText. 14
Figure G. Distribution of Most Enriched GO terms for Different Prioritization Methods. 14
Figure H. Most Enriched GO terms using hnInt versus noNet. 15
Figure I. Most Enriched GO terms using sText versus noNet. 15
Tables 15
Table H. List of IntOGen driver genes. 15
Table I. List of COSMIC driver genes. 15
Table J. List of cancer-specific IntOGen driver genes. 15
Table K. Cancer-specific prioritized genes. 15
Table L. Most significantly enriched GO terms using hnInt. 16
Table M. Most significantly enriched GO terms using sText. 16
Table N. Most significantly enriched GO terms using noNet. 16
Table O. P-value of the most significantly enriched GO terms. 16
Appendix C: Pan-Cancer Signature from Prioritized Genes **16**
Background 16
Results 17
Figures 17

Figure J. Survival Analysis of “expr_gp100_hnInt” Clustering......18
Tables......18
Table P. Analysis of Clustering with TCGA Expression Data......18
Table Q. Similarity Between Expression Clusterings......18
References......19

Appendix A: Feature Prioritization on TCGA PANCAN12 Transcriptomic Datasets

Overview

A widely popular application of high throughput omics profiling is identification of features (e.g. genes, proteins, mutations) likely to underlie particular phenotypic conditions or biological processes of interest. The Feature Prioritization pipeline in KnowEnG offers this general functionality, given a spreadsheet of features (features x samples) and a “phenotype spreadsheet” (phenotypes x samples) that represents one or more phenotypic labels for each sample in the feature spreadsheet. The features may correspond to omics data (e.g. expression of genes, somatic mutations, etc.), but they may also be more general (e.g. clinical features such as age).

Knowledge-Guided Feature Prioritization (Gene Prioritization)

One of the major capabilities provided in KnowEnG is knowledge-guided gene prioritization, which identifies genes that are most related to a phenotype of interest. For this mode of operation, the feature spreadsheet should contain gene-level omics measurements. To enable this capability, KnowEnG utilizes a generalized version of a computational tool called ProGENI [1], which we have previously developed. This method is built based on the premise that if known biological relationships of the genes (in the form of a gene interaction network) is properly included in this task, both the direct and indirect influence of the genes on the phenotype can be captured, resulting in a more accurate prioritization. In [1], this method was used to identify genes whose basal mRNA expression determines the sensitivity of cell lines to various drugs. Through extensive evaluations and follow-up siRNA knockdown experiments, we showed that this method significantly improves the accuracy of state-of-the-art gene prioritization methods [1].

Standard Gene Prioritization

In cases where the feature spreadsheet does not contain gene-level measurements, this pipeline supports standard feature prioritization. In this mode, the Knowledge Network is not used.

User Inputs

The Gene Prioritization pipeline has two primary inputs:

1. a required “feature spreadsheet” that contains measurements corresponding to different features of a collection of biological samples, and
2. a required “phenotype spreadsheet” that contains numerical (e.g., drug response, patient survival, etc.) or categorical (e.g., cancer subtype, metastatic status, experiment vs. control) information on the samples.

The feature spreadsheet should be formatted as a (features x samples) spreadsheet. The first column should contain the feature names and the first row should contain the sample names. If this spreadsheet contains missing values, several options are provided to handle these missing values (see User Parameters). The phenotype data is submitted as a separate spreadsheet formatted as samples x phenotype. The first column should contain the sample names and the

first row should contain the name of different phenotypes. The phenotype file can contain continuous-valued, binary, or categorical data. The missing values are allowed in the phenotype spreadsheet. For each phenotype, only samples with values will be used for the prioritization task. For more details about the input spreadsheets, see Figure C in S2 File and Figure D in S2 File in Appendix C in S2 File and data preparation in: [https://github.com/KnowEnG/quickstart-demos/blob/master/pipeline_readmes/README-DataPrep.md].

User Parameters

After submission of the two spreadsheets above, the user is given various options depending on their data and goal.

Global parameters

The user can choose how to deal with missing values in the omics spreadsheet:

- [omics_missing_value] - the options for handling missing values (NA) are
    - Average: Replace the missing value of a feature in a sample with the average expression of the same feature across other samples.
    - Remove: Remove any samples that contain missing values.
    - Reject: Reject the user's spreadsheet if any missing value exists.

Several parameters are needed for the underlying algorithm:

- The user is asked whether they would like to use the Knowledge Network. For the standard Feature Prioritization pipeline the user should choose 'No' and for the knowledge-guided mode 'Yes' .
- [primary_prioritization_method] - options are t-test (for binary) or absolute Pearson correlation (for continuous-valued phenotype). If the standard mode of this pipeline is used, the [primary_prioritization_method] is used for prioritization. In the standard mode, edgeR is an optional prioritization method that can be used when the supplied feature spreadsheet represents RNA-seq counts and the phenotype spreadsheet contains binary phenotypes. In the knowledge-guided mode, [primary_prioritization_method] is only one part of the whole prioritization pipeline. With a categorical phenotype (with more than two categories), a t-test should be chosen in standard Feature Prioritization mode and the pipeline will automatically identify genes related to one category vs. all other categories for every single category.
- [num_exported_features_per_phenotype] - the number of high priority features to return in the output result files that are formatted for further pipeline analysis.

If bootstrap sampling is selected, the following options need to be specified:

- [num_bootstraps] - an integer value
- [bootstrap_percentage] - percent of the samples to be randomly selected in each bootstrap sampling

Knowledge-Guided Only Parameters

To choose the network to be used in the analysis, the user should select:

- [species] - out of the twenty species in the Knowledge Network of the gene features.

- [interaction_network] - the gene-gene network available in the Knowledge Network for the selected species to use in the Gene Prioritization

Several parameters are needed for the underlying algorithm:

- [network_percentage] - controls the extent of influence the interaction network has in the transformation
- [num_response_correlated_features] - number of genes to be used as the restart set in the second RWR of the pipeline

Preprocessing of the data

Once the user selects the pipeline inputs and parameters, a simple preprocessing step occurs before the main algorithm.

Preprocessing of the feature spreadsheet: First, based on the user's selected option ([features_missing_value]), the missing values in the feature spreadsheet are handled. Then, rows or samples with duplicate names are dropped from the analysis. Also, if a feature name is missing, the corresponding row is removed from the analysis.

Preprocessing of the phenotype spreadsheet: First, there is a quick check to ensure that the sample names in the feature and phenotypic spreadsheets overlap, otherwise an error is returned. Missing values (NAs or NaNs) are accepted in the phenotype spreadsheet. If a phenotype of interest contains those values, samples for which the phenotype value is missing are not included in the analysis. If several phenotypes are provided in the phenotype spreadsheet, each phenotype is handled separately, ensuring that the maximum numbers of samples are used in the analysis. If t-test is chosen, the phenotype spreadsheet must contain phenotypes with at least two distinct categories, otherwise it is rejected. If number of categories is larger than two, then the phenotype spreadsheet is expanded to enable the comparison of each category with all other categories pooled together (one vs. all strategy).

Knowledge-Guided Only

In the knowledge-guided mode, the input gene names and identifiers of the feature spreadsheet are mapped to stable Ensembl identifiers of the appropriate [species] using the KN_Mapper tool and the Redis database of gene aliases that accompanies the current Knowledge Network build. Unmapped rows (either missing or ambiguous mappings) are dropped along with the rows that contain duplicated mapped gene identifier. Finally, the gene identifiers of the spreadsheet are compared to the gene identifiers of the selected [interaction_network]. If there is no intersection between these identifier lists, then the algorithm is terminated.

Description of Algorithm

Knowledge-Guided Mode:

Given the gene-level omics (feature) spreadsheet and the selected [interaction_network], this method first performs a network-based smoothing (using a random walk with restart algorithm, described in Appendix C in S2 File) of the omics data such that the new value assigned to each

gene also includes the omics characteristics of the gene's network neighbors, properly weighted to reflect the strength of their relationship. For details of this step, see [1]. Then, these smoothed values are compared with the phenotypic labels of the samples and using a statistical test ([primary_prioritization_method]) the top [num_response_correlated_features] genes are selected that are most "associated" (both positively and negatively) with the phenotype. This set of genes is then used as the restart set in an RWR algorithm on the same network, to obtain a score (i.e. equilibrium probability) for all the genes in the network. These scores are then normalized with respect to another score that represents the global topology of the network in order to remove the network bias. This normalized score is used to rank all the genes and identify top genes most related to the phenotype in light of the Knowledge Network. If bootstrap option is selected by the user, the procedure above is repeated [num_bootstraps] times, on randomly selected subsets of the samples (size of the subsets are specified using [bootstrap_percentage]) and the generated ranked lists of the genes are aggregated into a final robust ranking. A complete description of this method is provided in [1].

Standard Mode:

In this mode, the method used for feature prioritization is determined by [primary_prioritization_method]. The feature values of the samples are compared to the phenotypic labels of the samples and using a statistical test ([primary_prioritization_method]) features are ranked. If the bootstrap option is selected by the user, the procedure above is repeated [num_bootstraps] times on randomly selected subsets of the samples (size of the subsets are specified using [bootstrap_percentage]), and the generated ranked lists of the features are aggregated into a final robust ranking.

Pipeline Outputs

KnowEnG Platform Interface

The Feature Prioritization pipeline in the KnowEnG platform produces a visualization component that allows users to interactively view the results of the pipeline. The amount of data being visualized in the heatmap can be controlled by choosing the number of top features displayed per phenotype and turning on and off individual phenotypes. This allows users to both reduce the scale of the heatmap visualization and to focus on those aspects of the analysis that are of most interest. The users are also able to see "top features" score curves for each phenotype, which may help guide the investigation of results. Colored cells in the visualization heatmap represent the score of a given feature/phenotype correlation. Darker colors generally indicate a more significant correlation. Specifically, the darkest color in the color scale is always assigned to the greatest score found among all features for all of the displayed phenotypes. The lightest color in the color scale is assigned to the lowest score among the selected "top features" for all of the displayed phenotypes, and this assignment is updated whenever the user adjusts the number of "top features." Scores less than the lowest "top feature" score are mapped to gray. Interactive sorting options and heatmap cell rollovers further help to illuminate the results.

Downloadable Files

The downloadable zip archive contains several files. One file contains only the ranked list of features for each phenotype. Separately for each phenotype, there is a file that contains the

ranked list of features and the scores that were used to rank them and several other types of information. Also, one file contains the top ranked [num_exported_features_per_phenotype] features for each phenotype that is formatted such that it can be directly sent to the Gene Set Characterization pipeline. More information about the outputs of the pipeline and their structure can be found at [https://github.com/KnowEnG/quickstart-demos/blob/master/pipeline_readmes/README-FP.md].

Methods

In our second case study, we sought to identify genes associated with each tumor type in the PANCAN12 dataset [2]. We downloaded the expression spreadsheet and clinical annotations in the file "TCGA_PANCAN12_exp_HiSeqV2-2015-01-28.tgz" from the UCSC Cancer Genome Browser [3] in September 2017. This dataset contained expression of 16,114 genes in 3598 samples. To run the standard Feature Prioritization, we selected the 'average' option for [features_missing_value] to impute missing gene expression values. For each cancer type, the phenotype label of each sample represented whether the sample corresponds to the cancer type of interest ('1') or not ('0'). We used t-test as [primary_prioritization_method] for comparing the expression values and the phenotype and did not use the bootstrap sampling option.

For the knowledge-guided Gene Prioritization, we ran the analysis with both STRING text mining (sText) and HumanNet integrated (hnInt) network for [interaction_network] (see Appendix C in S2 File for more details on these networks) in separate tests. We selected the probability of restart as 0.5 (i.e. the [network_percentage] equal to 50), used t-test as [primary_prioritization_method] for comparing the smoothed expression values and the phenotype, selected the [num_response_correlated_features] as 50, and did not use the bootstrap sampling option.

The list of top 100 genes for each cancer type is provided in Tables A, B, and C in S3 Data when using the standard Feature Prioritization (noNet), the knowledge-guided prioritization with hnInt and with sText, respectively. Next, we compared the number of top genes (for each cancer type) that are identified using pairs of these methods (Table D in S3 Data). In average, approximately 52.72% of the identified genes differed between any two methods, with the biggest difference observed between hnInt and sText results with an average difference of 58.25%. Next, we compared the similarity between top 100 genes of any two cancer types using the same method. The results are provided in Tables E, F, and G in S3 Data for noNet, hnInt, and sText, respectively. The highest similarity was observed between colon_adenocarcinoma and rectum_adenocarcinoma with 78% using noNet, 71% using hnInt, and 67% using sText. head_&_neck_squamous_cell_carcinoma and lung_squamous_cell_carcinoma also showed a high degree of similarity with 27% using noNet, 31% using hnInt, and 50% using sText.

Resources

Gene Prioritization Pipeline

KnowEnG Platform Tool
[https://platform.knoweng.org/static/#/pipelines/feature_prioritization]
Quickstart Guide [https://knoweng.org/wp-content/uploads/2017/08/GP_Quickstart.pdf]

YouTube Tutorial [https://www.youtube.com/watch?v=Vp76-Oz-Yuc]
Data Preparation Guidelines [https://github.com/KnowEnG/quickstart-demos/blob/master/pipeline_readmes/README-DataPrep.md]
Downloadable Results Description [https://github.com/KnowEnG/quickstart-demos/blob/master/pipeline_readmes/README-FP.md]

Seven Bridges Cancer Genomics Cloud
Public Tool [https://cgc.sbgenomics.com/public/apps#mepstein/knoweng-geneprioritization-public/gene-prioritization-workflow/]
Quickstart Guide [https://knoweng.org/wp-content/uploads/2018/02/GP_CGC_Quickstart.pdf]

Docker and GitHub Repositories
Knowledge-Guided Docker [https://hub.docker.com/r/knowengdev/gene_prioritization_pipeline/]
Knowledge-Guided GitHub [https://github.com/KnowEnG/Gene_Prioritization_Pipeline]
Standard Docker [https://hub.docker.com/r/knowengdev/feature_prioritization_pipeline/]
Standard GitHub [https://github.com/KnowEnG/Feature_Prioritization_Pipeline]
Data Cleanup Docker [https://hub.docker.com/r/knowengdev/data_cleanup_pipeline/]
Data Cleanup GitHub [https://github.com/KnowEnG/Data_Cleanup_Pipeline]
Pipeline Utilities Docker [https://hub.docker.com/r/knowengdev/base_image/]
Pipeline Utilities GitHub [https://github.com/KnowEnG/KnowEnG_Pipelines_Library]

Tables

Table A. Top 100 genes without using the Knowledge Network.

This table provides a ranked list of top 100 genes identified for each cancer type using the standard mode of operation of Gene/Feature Prioritization pipeline (without the use of Knowledge Network).

Table B. Top 100 genes using HumanNet Integrated Network.

This table provides a ranked list of top 100 genes identified for each cancer type using the knowledge-guided mode of operation of Gene Prioritization pipeline using the HumanNet Integrated Network.

Table C. Top 100 genes using STRING Text Mining.

This table provides a ranked list of top 100 genes identified for each cancer type using the knowledge-guided mode of operation of Gene Prioritization pipeline using the STRING Text Mining Network.

Table D. Cross-dataset Intersection.

This table provides the number of top 100 genes that are shared between the results of different methods for each cancer type.

Table E. Intersections of Top Gene Results using No Network Knowledge.
This table provides the number of top 100 genes that are shared between different cancer types, where the top genes are identified using the standard (noNet) mode of Feature Prioritization.

Table F. Intersections of Top Gene Results using HumanNet Integrated.
This table provides the number of top 100 genes that are shared between different cancer types, where the top genes are identified using the knowledge-guided mode of operation of the Gene Prioritization pipeline using the HumanNet Integrated network.

Table G. Intersections of Top Gene Results using STRING Text Mining.
This table provides the number of top 100 genes that are shared between different cancer types, where the top genes are identified using the knowledge-guided mode of operation of the Gene Prioritization pipeline using the STRING Text Mining network.

Appendix B: Characterization of Top Genes Identified for Each Tumor Type

Methods

To assess the relevance of the genes identified using knowledge-guided as well as standard modes of Feature Prioritization pipeline for each cancer type (Appendix A in S3 File), we obtained the list of cancer driver genes from IntOGen] [www.intogen.org] [4] and COSMIC [https://cancer.sanger.ac.uk/cosmic] [5]. The list of driver genes from IntOGen, which contained 475 genes, is shown in Table H in S3 Data. The list of driver genes from COSMIC, which contained 699 genes, is shown in Table I in S3 Data.

Characterization of top genes using cancer-specific driver genes

First, we sought to determine the overlap between top genes identified for each cancer type (referred to as hnInt, sText, and noNet following the notation in Appendix A in S3 File) with the driver genes of the same cancer in IntOGen. IntOGen did not contain driver genes for KIRC. Also, the driver genes for COAD and READ were reported in a single group. Table J in S3 Data shows the list of driver genes annotated in IntOGen, separately for each cancer type present in the PANCAN12 dataset. Next, we used the standard mode of KnowEnG Gene Set Characterization pipeline (which implements Fisher's Exact test) to compare the list of top 100 genes identified for each cancer type with the drivers of that cancer (see Appendix C in S4 File for a description of this pipeline). Figures A and B in S3 File show the number of driver genes (and their corresponding p-values) for hnInt vs. noNet and sText vs. noNet, respectively. As can be seen in these figures, for five (out of eleven) cancer types, the enrichment p-values of top genes identified using knowledge-guided analysis (hnInt or sText) are smaller than 0.05, while this is true only for one cancer type when using the standard mode of Gene Prioritization (noNet). Table K in S3 Data lists the prioritized cancer specific drivers from Figure A in S3 File, as well as an indicator if the driver was found by the noNet or hnInt methods or both.

Characterization of top genes using all driver genes

Next, we sought to determine the overlap between top genes identified for each cancer type with all the driver genes in IntOGen and COSMIC. Similar to the previous analyses, we used the standard mode of KnowEnG Gene Set Characterization pipeline to compare the list of top 100 genes identified for each cancer type with cancer driver genes. Figures C and D in S3 File show the number of driver genes from IntOGen (and their corresponding p-values) for hnInt vs. noNet and sText vs. noNet, respectively. As can be seen in these figures, the enrichment p-value of top genes identified using hnInt for six cancer types and using sText for five cancer types are smaller than 0.05, while this is true only for two cancer types when using the standard mode of Gene Prioritization (noNet).

Figures E and F in S3 File show the number of driver genes from COSMIC (and their corresponding p-values) for hnInt vs. noNet and sText vs. noNet, respectively. As can be seen in these figures, the enrichment p-value of top genes identified using hnInt for six cancer types and using sText for five cancer types are smaller than 0.05, while this is true only for two cancer types when using the standard mode of Gene Prioritization (noNet).

Characterization of top genes using all driver genes

To gain further insights into the highly ranked genes reported for each tumor type, we performed functional enrichment analysis using KnowEnG's Gene Set Characterization pipeline (standard mode) to identify Gene Ontology (GO) terms most associated with these gene sets. Tables L, M, and N in S3 Data show the top 10 enriched GO terms for each cancer type, where the top 100 genes are identified using hnInt, sText, and noNet, respectively. The extent to which significant functional properties can be associated with a gene set extracted by genomics analyses is one measure of the utility of that gene set [6]. Thus, we summarized the results of gene set characterization by noting the most statistically significant functional enrichment (of genes prioritized) for each tumor type (Table O in S3 Data). We noted that the enrichments were significantly more prominent when genes were identified using hnInt compared to noNet ($p=2.2E-3$, Wilcoxon signed rank test), as depicted in Figures G and H in S3 File. Similarly, the enrichments were significantly more prominent when genes were identified using sText compared to noNet ($p=4.4E-3$, Wilcoxon signed rank test), as depicted in Figures G and I in S3 File. These results provide further evidence of the value of knowledge-guided gene prioritization.

Figures

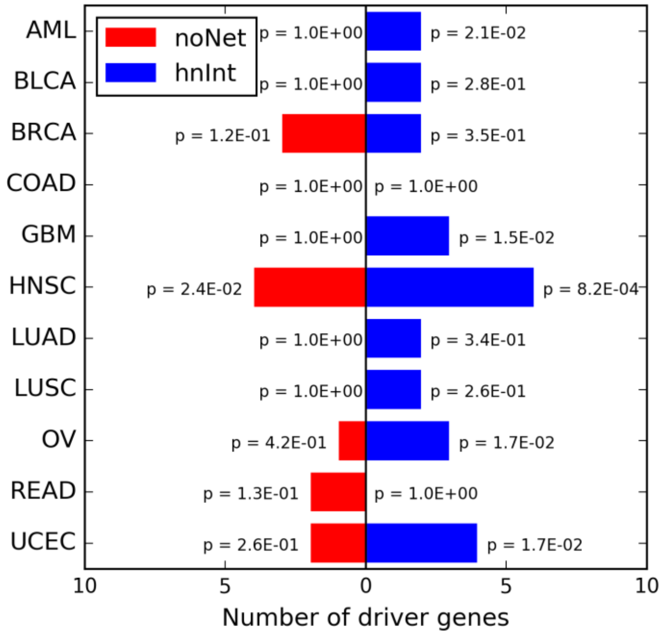

Figure A. IntOGen Cancer-Specific Drivers Identified using noNet and hnInt. The bars represent the number of cancer-specific driver genes (from IntOGen) among the top 100 genes identified using noNet (red) and hnInt (blue) for each cancer type. The p-values represent the significance of enrichment, calculated using Fisher's exact test.

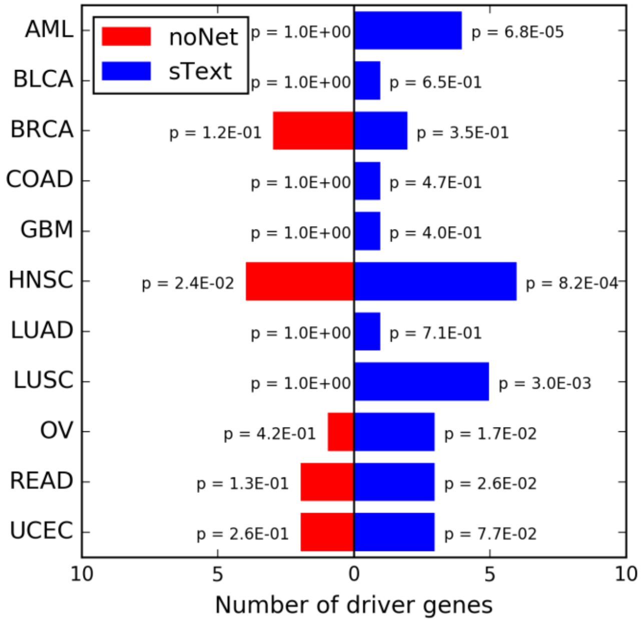

Figure B. IntOGen Cancer-Specific Drivers Identified using noNet and sText.
The bars represent the number of cancer-specific driver genes (from IntOGen) among the top 100 genes identified using noNet (red) and sText (blue) for each cancer type. The p-values represent the significance of enrichment, calculated using Fisher's exact test.

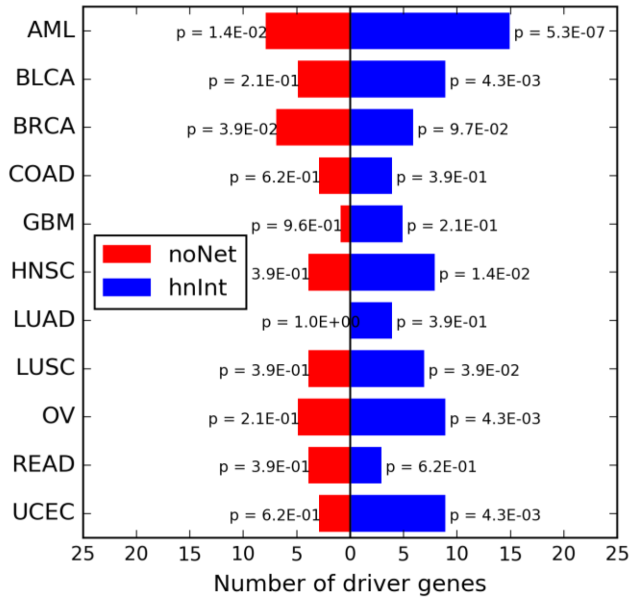

Figure C. IntOGen Driver Genes among Top Genes Identified using noNet and hnInt.
The bars represent the number of driver genes (from IntOGen) among the top 100 genes identified using noNet (red) and hnInt (blue) for each cancer type. The p-values represent the significance of enrichment, calculated using Fisher's exact test.

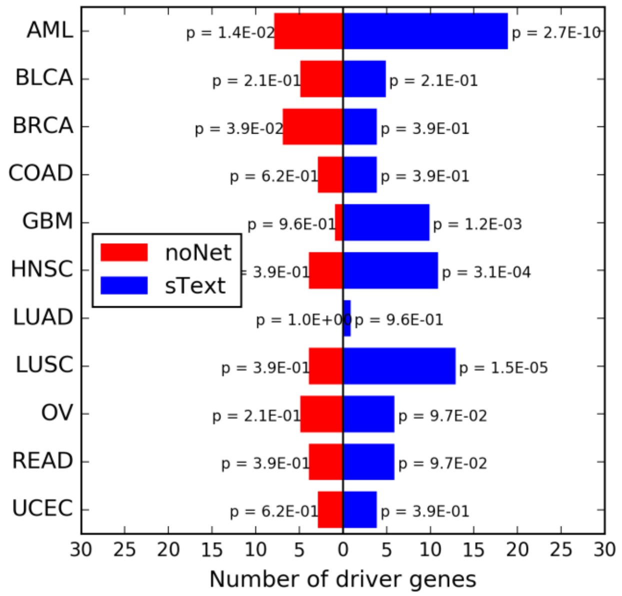

Figure D. IntOGen Driver Genes among Top Genes Identified using noNet and sText.

The bars represent the number of driver genes (from IntOGen) among the top 100 genes identified using noNet (red) and sText (blue) for each cancer type. The p-values represent the significance of enrichment, calculated using Fisher's exact test.

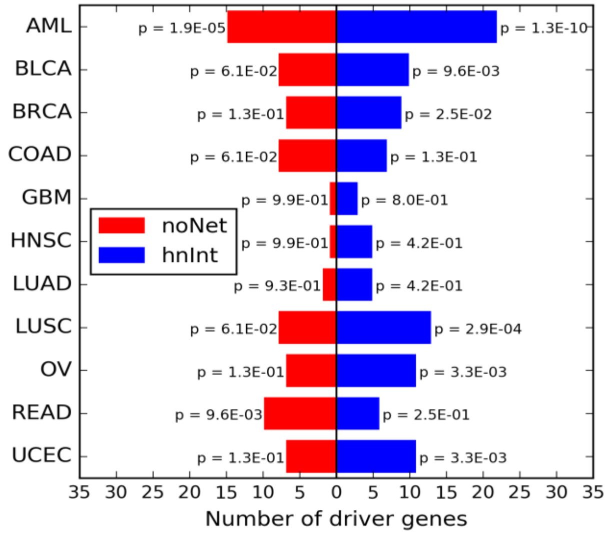

Figure E. COSMIC Driver Genes among Top Genes identified Using noNet and hnInt.

The bars represent the number of driver genes (from COSMIC) among the top 100 genes identified using noNet (red) and hnInt (blue) for each cancer type. The p-values represent the significance of enrichment, calculated using Fisher's exact test.

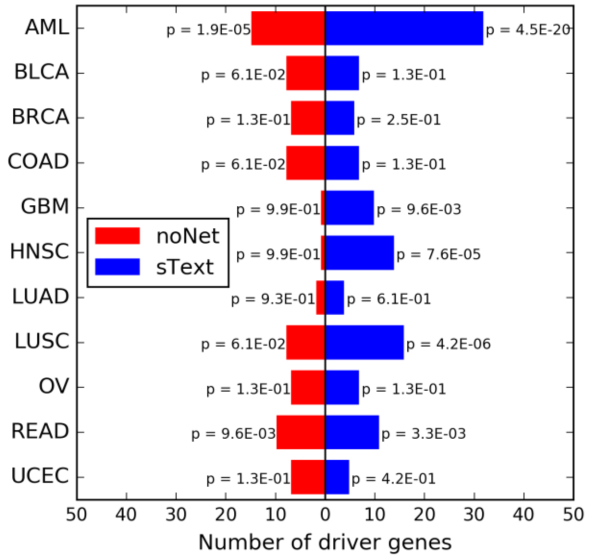

Figure F. COSMIC Driver Genes among Top Genes identified Using noNet and sText.

The bars represent the number of driver genes (from COSMIC) among the top 100 genes identified using noNet (red) and sText (blue) for each cancer type. The p-values represent the significance of enrichment, calculated using Fisher's exact test.

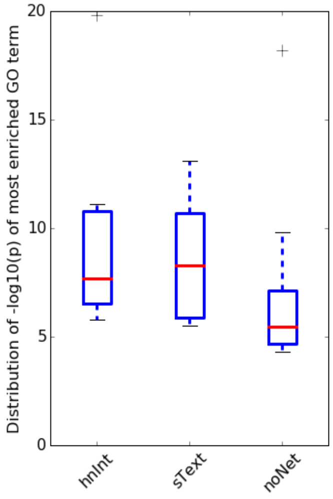

Figure G. Distribution of Most Enriched GO terms for Different Prioritization Methods.

For each cancer type and each prioritization method, the list of top 100 genes is identified and GO enrichment analysis (using Fisher's exact test) is performed to identify the most significantly enriched GO term. Each box shows the distribution of -log10(p) of these significantly enriched GO terms for different cancer types. The red line represents the median.

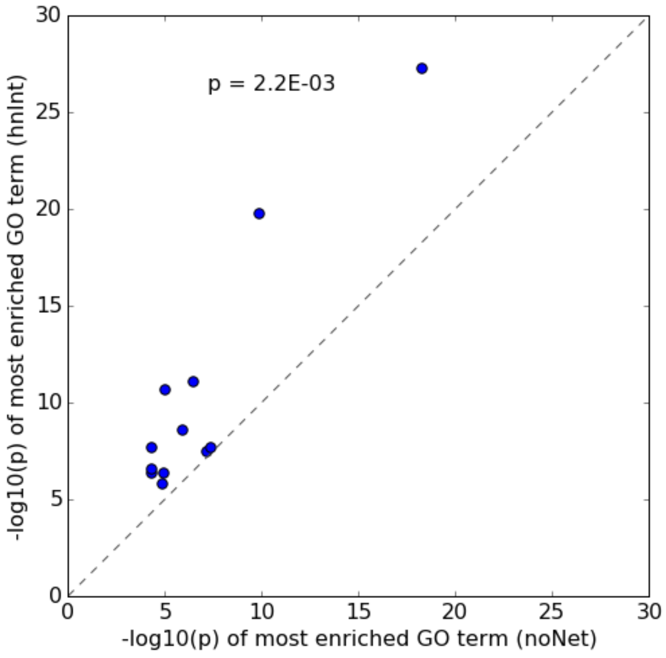

Figure H. Most Enriched GO terms using hnInt versus noNet.
Each circle represents one cancer type. The p-value of improvement is calculated using Wilcoxon signed rank test.

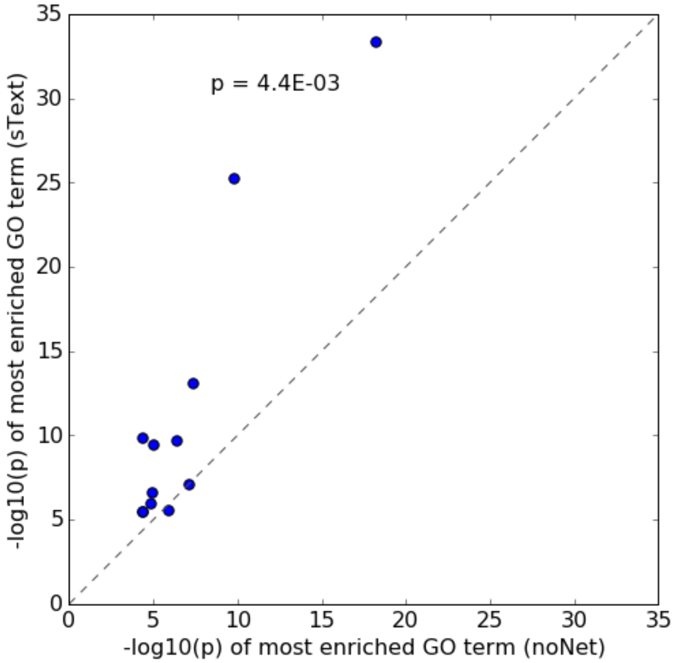

Figure I. Most Enriched GO terms using sText versus noNet.
Each circle represents one cancer type. The p-value of improvement is calculated using Wilcoxon signed rank test.

Tables

Table H. List of IntOGen driver genes.
This table provides the list of all the driver genes in IntOGen.

Table I. List of COSMIC driver genes.
This table provides the list of all the driver genes in COSMIC.

Table J. List of cancer-specific IntOGen driver genes.
This table shows the list of driver genes for each cancer type present in the PANCAN12 dataset.

Table K. Cancer-specific prioritized genes.
This table lists all of the prioritized genes from the HumanNet Integrated network ‘hnInt’ and no Knowledge Network ‘noNet’ runs that are also cancer-specific genes for the matching cancer type. It list the ‘gene’ name, ‘ensembl’ gene id, the ‘cancer’ type for each gene. The ‘set_type’

column separate genes into three categories: those found in ‘both’ runs, or those found only in the network guided run for ‘hnInt_only’ or the other ‘noNet_only’.

Table L. Most significantly enriched GO terms using hnInt.

This table shows the top 10 GO terms and their enrichment p-values for each cancer type present in PANCAN12 dataset. The enrichment analysis was performed using the standard mode of KnowEnG’s Gene Set Characterization pipeline (using Fisher’s exact test). The gene sets for each cancer type contained the top 100 genes identified using knowledge-guided gene prioritization with hnInt.

Table M. Most significantly enriched GO terms using sText.

This table shows the top 10 GO terms and their enrichment p-values for each cancer type present in PANCAN12 dataset. The enrichment analysis was performed using the standard mode of KnowEnG’s Gene Set Characterization pipeline (using Fisher’s exact test). The gene sets for each cancer type contained the top 100 genes identified using knowledge-guided gene prioritization with sText.

Table N. Most significantly enriched GO terms using noNet.

This table shows the top 10 GO terms and their enrichment p-values for each cancer type present in PANCAN12 dataset. The enrichment analysis was performed using the standard mode of KnowEnG’s Gene Set Characterization pipeline (using Fisher’s exact test). The gene sets for each cancer type contained the top 100 genes identified using standard Gene Prioritization (noNet).

Table O. P-value of the most significantly enriched GO terms.

This table shows the top p-value associated with the most enriched GO term for each cancer type and each prioritization method.

Appendix C: Pan-Cancer Signature from Prioritized Genes

Background

The second case study was about using knowledge-guided Gene Prioritization in order to identify lists of 100 genes that were specific to each pancan12 cancer disease type (Appendix A in S3 File). We then sought to identify whether these sets of prioritized genes were able to form a ‘signature’ for tumor type, i.e., a representative collection of genes that captures much of the diagnostic or prognostic value of the entire expression profile. In order to perform this analysis, we created a union gene set from the twelve tumor type specific top100 gene lists. We repeated this process for all three gene prioritization analyses each run with different prior knowledge,

1) no network, ‘noNet’ (Table A in S3 Data),
2) HumanNet Integrated network, ‘hnInt‘ (Table B in S3 Data).
3) STRING TextMining, ‘SText’ (Table C in S3 Data)

These three union gene sets resulted in 993, 985, and 963 genes respectively. We then used our standard hierarchical-based Sample Clustering (Appendix C in S2 File) with ‘Euclidean’ affinity and ‘Ward’ linkage separately to find 16 clusters (as done in original pancan12 analysis [2]) using the full pancan12 14373 gene expression matrix (expr_All), and that expression matrix subsetted for each of our three union gene sets (expr_gp100_noNet, expr_gp100_hnInt, expr_gp100_sText).

Results

Table P in S3 Data shows the details and the Kaplan-Meier survival analysis p-values for the clusters resulting from these four sample clusterings. Indeed, we observed that pan-cancer subtypes obtained from clustering only the expression of the HumanNet Integrated network informed tumor-associated genes were just as predictive of survival (Kaplan Meier p-value 3.8E-175, Figure J in S3 File) as the above-mentioned clusters based on entire expression profiles (p-value 1.2E-169). This result held whether we used a different network, STRING TextMining, or no network in the Gene Prioritization step. This means we can reduce from ~14K to 1K genes without losing the ability to identify meaningful clusters that relate to overall survival. Interestingly, although the clusters identified by this method had slightly improved survival outcome predictive ability, they were not as strongly related to tumor type as the clusters identified with the expression data and the Cluster-Of-Cluster-Assignments (COCA) in the original paper [2]. Neither were these new clusters strongly similar to each other, suggesting that each different modality of Gene Prioritization enable the discovery of a different by equally good clustering that relates to survival separation (Table Q in S3 Data).

Figures

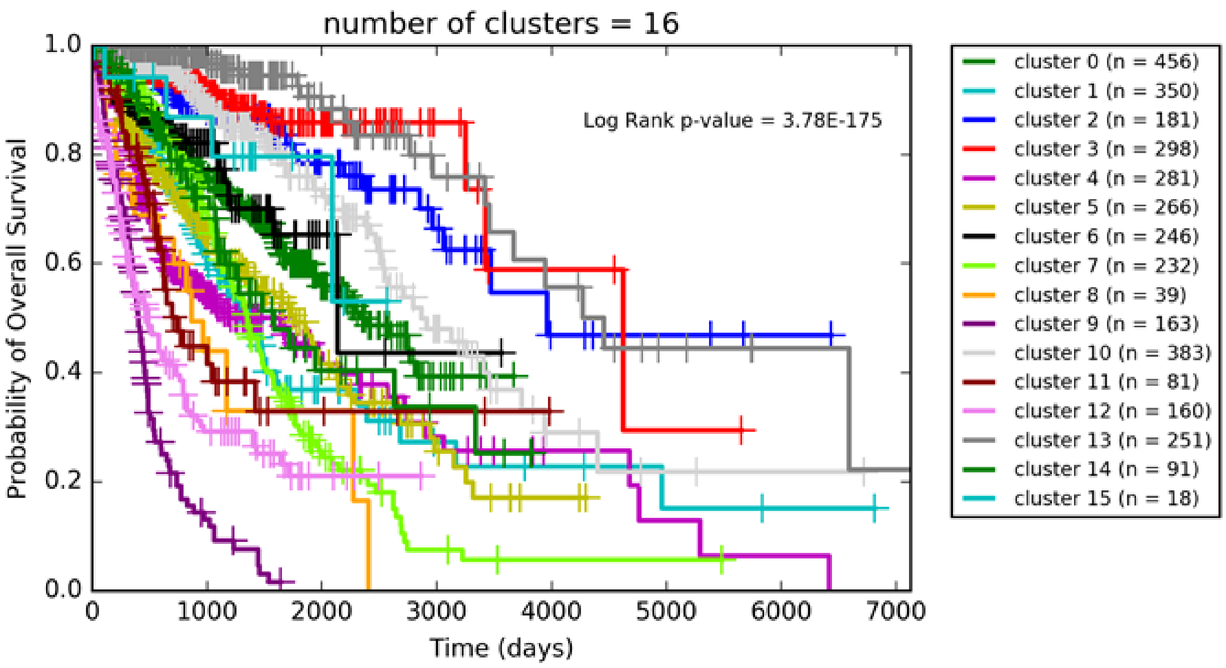

Figure J. Survival Analysis of “expr_gp100_hnInt” Clustering.
Each cluster is plotted as a separate survival curve in the Kaplan-Meier plot and the p-value of the multivariate log rank test is reported.

Tables

Table P. Analysis of Clustering with TCGA Expression Data.
Shows statistics for various methods for clustering the gene expression data. The first column ('Alias') shows the name given to each clustering run. The first three rows show clusterings from the original PANCAN12 paper analysis and the last four rows show the results of our General Clustering pipeline using Ward linkage with Euclidean affinity on the PANCAN12 gene expression data. The final three rows only use genes that were in the top 100 prioritized gene lists for disease type. We report the number of clusters ('nClust'), the number of gene features used in the clustering ('nFeatures') and the Kaplan-Meier p-value of the significance of the relationship between the clustering and survival outcome.

Table Q. Similarity Between Expression Clusterings.
Shows the adjusted Rand Index for each pairing of the seven selected clusterings in this analysis: “disease” - grouping by TGCA primary disease, and “tcga_expr” and “tcga_coca”, expression-only and COCA clustering analysis in original TCGA paper, and “expr_gp100_hnInt”, “expr_gp100_sText”, and “expr_gp100_noNet”- clustering of expression data using genes from gene prioritization for disease type with HumanNet Integrated, STRING Textmining, and no prior knowledge networks respectively.

References

1. Emad A, Cairns J, Kalari KR, Wang L, Sinha S. Knowledge-guided gene prioritization reveals new insights into the mechanisms of chemoresistance. Genome Biol. 2017;18(1):153. doi: 10.1186/s13059-017-1282-3. PubMed PMID: 28800781; PubMed Central PMCID: PMCPMC5554409.
2. Hoadley KA, Yau C, Wolf DM, Cherniack AD, Tamborero D, Ng S, et al. Multiplatform analysis of 12 cancer types reveals molecular classification within and across tissues of origin. Cell. 2014;158(4):929-44. doi: 10.1016/j.cell.2014.06.049. PubMed PMID: 25109877; PubMed Central PMCID: PMCPMC4152462.
3. Goldman M, Craft B, Swatloski T, Cline M, Morozova O, Diekhans M, et al. The UCSC Cancer Genomics Browser: update 2015. Nucleic Acids Res. 2015;43(Database issue):D812-7. doi: 10.1093/nar/gku1073. PubMed PMID: 25392408; PubMed Central PMCID: PMCPMC4383911.
4. Rubio-Perez C, Tamborero D, Schroeder MP, Antolin AA, Deu-Pons J, Perez-Llamas C, et al. In silico prescription of anticancer drugs to cohorts of 28 tumor types reveals targeting opportunities. Cancer Cell. 2015;27(3):382-96. doi: 10.1016/j.ccell.2015.02.007. PubMed PMID: 25759023.
5. Forbes SA, Beare D, Boutselakis H, Bamford S, Bindal N, Tate J, et al. COSMIC: somatic cancer genetics at high-resolution. Nucleic Acids Res. 2017;45(D1):D777-D83. doi: 10.1093/nar/gkw1121. PubMed PMID: 27899578; PubMed Central PMCID: PMCPMC5210583.
6. Choobdar S, Ahsen ME, Crawford J, Tomasoni M, Lamparter D, Lin J, et al. Open Community Challenge Reveals Molecular Network Modules with Key Roles in Diseases. bioRxiv. 2018:265553. doi: 10.1101/265553.
