# Supplementary material for: Knowledge-guided analysis of "omics" data using the KnowEnG cloud platform
Source: PLoS Biol. 2020 Jan 23;18(1):e3000583. doi: 10.1371/journal.pbio.3000583 (PMC6977717; doi:10.1371/journal.pbio.3000583)
Supplement: S4 File — Four appendices describing the data, pipelines, methods, and additional analyses that relate to the third case study of using KnowEnG pipelines to analyze LUSC signatures in ESCC samples on a third-party system. ESCC, esophageal squamous cell carcinoma; KnowEnG, Knowledge Engine for Genomics; LUSC, lung squamous cell carcinoma. (PDF) [file pbio.3000583.s004.pdf]

S4 File: Signature Analysis and Gene Set Characterization

Table of Contents

Table of Contents 1
Methods 3
Resources 4
Spreadsheet Builder Pipeline 4
Tables 4
Table A. SB-CGC Datasets. 4
Appendix B: KnowEnG Signature Analysis on LUSC Subtypes 4
Overview 4
User Inputs 4
User Parameters 5
Data Preprocessing 5
Description of Algorithm 5
Pipeline Outputs 5
Methods 6
Resources 7
Tables 8
Table B. Best Match Signatures of ESCC Samples. 8
Table C. Best Match Signatures of LUSC Samples. 8
Table D. Signature Analysis of ESCA and LUSC Samples. 8
Table E. Top100 Gene Sets for Each Tumor Subtype from ESCC Samples. 8
Table F. Overlap Between Top100 Gene Sets. 8
Appendix C: KnowEnG Gene Set Characterization 8
Overview 8
User Inputs 9
User Parameters 9
Data Preprocessing 10
Description of Algorithm 10
Pipeline Outputs 11
Methods 11
Resources 12
Figures 13
Figure A. Overview of Knowledge-Guided Gene Set Characterization. 13
Tables 13
Table G. Comparison of Results on ESCC Subtype Gene Sets. 13
Table H. Shared Results on ESCC Subtype Gene Sets. 14
Table I. DRaWR Specific Results on ESCC Subtype Gene Sets. 14
Appendix D: Consistency Analysis of DRaWR 14
Overview 14
Results 14
Recovering Annotations with Biased User Gene Sets 14
Recovering Annotations with Partial User Gene Sets 16
Tables 17
Table J. dbGaP and DisGeNet Disease Gene Sets Mapping. 17

Table K. GSC Mode Comparison with dbGaP Test Sets. 17
Table L. Summary of GSC Mode Comparison with dbGaP. 17
Table M. GSC Mode Comparison with Subset Test Sets. 17
Table N. Summary of GSC Mode Comparison with Subsets. 18
References 19

Appendix A: Selection of TCGA Data from SB-CGC

Methods

In this case study, we sought to recreate part of the genomic subtype signature analysis performed by the Cancer Genome Atlas Research Network in their recent study on the genomic characterization of oesophageal carcinoma [1]. We decided to recreate this analysis, not only with KnowEnG developed tools, but also as demonstration of the portability of those tools. For this reason, all of the analyses in this case study are run with our tools on the Seven Bridges [https://www.sevenbridges.com/] NCI Cancer Genomic Cloud (SB-CGC) [http://www.cancergenomicscloud.org/].

The first step of this case study was to extract the relevant gene expression datasets from the SB-CGC and reformat them into a transcriptomics spreadsheet to be analyzed by KnowEnG pipelines. We created two datasets, one for Esophageal Carcinoma (ESCA), the original samples studied in the TCGA paper [1], and the other for Lung Squamous Cell Carcinoma (LUSC), a related cancer type. In order to create each of these datasets, we used the SB-CGC "Data Browser" feature to explore and select the appropriate TCGA RNA-seq data. To do this, a user 1) selects the "TCGA GRCh38" dataset, 2) adds files with "Data category" = "Transcriptome Profiling" and "Data format" = "TXT", and 3) and selects investigations where the "Disease Type" is one of the two disease types of interest, either ESCA or LUSC. This produces a list of files for each cancer type, which then need to be copied to an SB-CGC project. When we performed these steps, each sample produced three files, '*.htseq.counts.tz', *.FPKM-UQ.txt', '*.FPKM.txt'. For our recreation, we kept only the '*.FPKM.txt' files and deleted the other two types. More detailed information about this selection process can be found here [https://github.com/KnowEnG/KnowEnG_CWL/tree/master/CGC#gathering-the-tcga-input-files].

This selection process obtained 173 files for ESCA samples and 551 files LUSC samples (Table A in S8 Data). We then used the KnowEnG Spreadsheet Builder, a custom tool built specifically for converting TCGA files and their metadata on the SB-CGC into omics and phenotypic spreadsheets for KnowEnG Analysis. For each of the two datasets, we ran the Spreadsheet Builder workflow [https://cgc.sbgenomics.com/public/apps#mepstein/knoweng-spreadsheetbuilder-public/spreadsheet-builder/] on SB-CGC using the FPKM files as inputs. We ran these workflows using the 'sample_id' as the "Metadata Sample ID Key" that matches samples between the omics and phenotypic spreadsheets. We also ran with a Filter Threshold of "1" and a Filter Minimum Percentage of "0.1", which filtered out genes that did not have a FPKM of at least 1 in at least 10% of samples. After filtering, we were left with 17842 gene features. Finally, we set the Spreadsheet Builder "Normalization Flag" which scales the log2(FPKM +1) to mean zero and standard deviation 1 and outputs these z-scores of the log transformed expression values. Each of these two workflow runs took 5-10 minutes on the SB-CGC, costing ten cents in direct AWS costs, using spot instances. The runs produced a transcriptomic spreadsheet for each cancer type and a phenotypic spreadsheet that contained the corresponding clinical TCGA data. The combined phenotypic spreadsheets are available in Table A in S8 Data.

Resources

Spreadsheet Builder Pipeline

Seven Bridges Cancer Genomics Cloud

Spreadsheet Builder Workflow Tool
[https://cgc.sbgenomics.com/public/apps#mepstein/knoweng-spreadsheetbuilder-public/spreadsheet-builder/]
Tutorial for Workflow [https://github.com/KnowEnG/KnowEnG_CWL/tree/master/CGC#running-the-spreadsheet-builder-workflow]

Docker and GitHub Repositories

Spreadsheet Builder CWL [https://cgc.sbgenomics.com/raw/mepstein/knoweng-spreadsheetbuilder-public/spreadsheet-builder/1]
Spreadsheet Builder Docker [https://hub.docker.com/r/mepsteindr/spreadsheet_preprocess/]
Spreadsheet Builder GitHub [https://github.com/KnowEnG/SpreadsheetPreprocess]
Pipeline Utilities Docker [https://hub.docker.com/r/knowengdev/base_image/]
Pipeline Utilities GitHub [https://github.com/KnowEnG/KnowEnG_Pipelines_Library]

Tables

Table A. SB-CGC Datasets.

Shows the phenotypic information about the RNA-seq samples extracted from the SB-CGC TCGA dataset for ESCA and LUSC cancer types. Three columns were added to the left side of the table as an indicator of which samples relate to which cancer type. The first column shows which samples were mapped to ESCC subgroups in the original analysis.

Appendix B: KnowEnG Signature Analysis on LUSC Subtypes

Overview

The KnowEnG Signature Analysis pipeline is a basic analysis tool that calculates the similarity between the shared features of a sample and a known omics signature. This type of analysis is useful in bioinformatics when you are given a large collection of samples described by their “omics” (genomic, transcriptomic, etc.) profiles and want to map them to a limited library of subtypes characterized from previous studies.

User Inputs

The Signature Analysis pipeline has two primary inputs:

1. A required “omics spreadsheet” that will have its samples mapped to the subtype signatures based on the similarity between their feature profiles, and

2. A required “signature spreadsheet” that contains the omics features and average values of known omics subtypes of interest.

Both the “omics” and “signature” spreadsheet are expected to be a matrices of numeric values with named rows representing features and named columns representing samples or signatures respectively (see Figure C in S2 File). It is important that the measurements represented in the matrices are similarly derived and therefore appropriate for comparison, e.g. both spreadsheets contain gene expression values and were normalized in analogous ways. Also, each feature’s name must match exactly between the omics and signature spreadsheet for that feature to be included in the comparison. More information about the formatting of the input files can be found at our data preparation resource [https://github.com/KnowEnG/quickstart-demos/blob/master/pipeline_readmes/README-DataPrep.md].

User Parameters

There is only a single parameter that the user must select to run the Signature Analysis Pipeline:

- [similarity_measure] to define the similarity between each sample and signature. Currently ‘cosine’ similarity is supported as well as ‘Pearson’ and ‘spearman’ correlation.

Data Preprocessing

A simple preprocessing step occurs before the main Signature Analysis algorithm that checks that there are no missing or non-numeric values and then extracts and reorders the shared features between the omics and signature spreadsheets.

Description of Algorithm

Once the omics and signature spreadsheet are harmonized with the shared features in the same order, the KnowEnG Signature Analysis pipeline calculates the similarity between every sample in the omics spreadsheet and every signature using the [similarity_measure] selected by the user.

Pipeline Outputs

KnowEnG Platform Interface

Running the KnowEnG Signature Analysis pipeline in the KnowEnG Platform will produce results that can be viewed interactively. The Signature Analysis visualization, similar to the Feature Prioritization pipeline visualization, provides a panel that enables control of the amount of data being visualized in the primary heatmap result. By filtering on a signature score threshold across samples and turning on and off individual signatures, the user can both reduce the scale of the heatmap visualization and focus on those aspects of the analysis that are of most interest. The user is also able to see score curves for samples by signatures, which may help guide the investigation of results.

Downloadable Files

There are two primary downloadable files of the Signature Analysis pipeline. The first is a signatures by samples matrix where each cell contains the calculated similarity value. The

second is a samples by signatures matrix of indicator 0/1 values, where a 1 indicates that the signature is the best match for the input sample. More information about the outputs of the pipeline and their structure can be found at [https://github.com/KnowEnG/quickstart-demos/blob/master/pipeline_readmes/README-SA.md].

Methods

For this case study, we wished to reproduce Extended Data Figure 6 [https://www.nature.com/articles/nature20805/figures/12] of the TCGA analysis of oesophageal carcinomas [1]. In order to do this, we needed first to retrieve the LUSC genomics subtypes collected for the original analysis. These lung squamous cell carcinoma signatures were created from mRNA expression measured by microarrays in a study by Wilkerson et al. [2]. We downloaded the “predictor.centroids” data from this study from the supplementary website [http://cancer.unc.edu/nhayes/publications/scc/]. This file contains four subtypes of LUSC, ‘basal’, ‘primitive’, ‘classical’, and ‘secretory’ described by their normalized expression values of 208 genes. We used the “File” management features of the SB-CGC to upload this signature spreadsheet into our project.

We next ran the Signature Analysis workflow [https://cgc.sbgenomics.com/public/apps#mepstein/knoweng-signature-analysis-public/] in the SB-CGC for each of two transcriptomic samples spreadsheets we had constructed previously, ESCC and LUSC (see Appendix A in S4 File). In the SB-CGC implementation, the workflow maps the gene aliases from both the samples and the signatures spreadsheets to common Ensembl identifiers using the KN_Mapper (see Appendix E in S1 File) before running the Signature Analysis pipeline, so we provided the [species_taxon_id] of ‘9606’ for human. For each samples spreadsheet, we provided our LUSC signatures and used the ‘spearman’ correlation as our [similarity_measure] to run the Signature Analysis pipeline. Each workflow run took under 5 minutes to run and cost five cents using spot instances. Eleven of the original 208 signature genes were unmapped by KN_Mapper.

For the “similarity_matrix” output file from the run with ESCA samples, we extracted 79 samples that were flagged as esophageal squamous cell carcinoma (ESCC) in the original analysis [1]. Note, eleven of the 90 samples in the original analysis are not present in the SB-CGC TCGA dataset. These ESCC samples were placed into one of three subgroups by the original analysis (ESCC1, ESCC2, and ESCC3), which we make available in the first column of Table A in S8 Data. We removed negative Spearman correlations and plot the correlations of the samples to the 4 LUSC subtypes in the style of Extended Data Figure 6 from the original paper [1] (see Fig 5C and Table B in S8 Data). We also repeated this process from the “similarity_matrix” output file from the run with LUSC samples to produce Fig 5D (Table C in S8 Data). For the 5 groups of samples “ESCC1”, “ESCC2”, “ESCC3”, “other ESCA”, and “LUSC”, we calculated the average non-negative correlation of these samples to each subtype and counted the number of samples with a best match to each subtype (Table D in S8 Data). The results are not identical to the results presented in the original Extended Data Figure 6 for a few possible reasons: 1) the normalization procedure in the original paper does not match the SB-CGC FPKM data and our

normalization, 2) the original figure used Pearson rather than Spearman correlation, 3) a few genes were removed from our correlation calculation due to mapping ambiguities.

We continued the analysis of the 79 ESCC samples by finding out for each tumor subtype, which genes were differentially expressed between the samples that mapped to that subtype and the samples that mapped to other subtypes. This was done using the standard mode of the KnowEnG Feature Prioritization pipeline (see Appendix A of S3 File) on the SB-CGC [https://cgc.sbgenomics.com/public/apps#workflow/mepstein/knoweng-geneprioritization-public/gene-prioritization-workflow]. We took the assignment matrix of each ESCC sample to its best match signature (similarity_matrix.binary.tsv) from the ESCA Signature Analysis run and filtered for the 79 ESCC samples. We then launched the Feature (Gene) Prioritization workflow on the SB-CGC. For the omics spreadsheet input, we used the ESCA transcriptomic samples spreadsheet that was also the input to Signature Analysis. For the phenotypic spreadsheet input, we used the assignment matrix mentioned above. We also provided the species taxonomy identifier again (9606) and requested the 't_test' [primary_prioritization_method]. We chose to not run the knowledge-guided mode, so no network related parameters were specified. The method was also run without bootstrap sampling. We ran this workflow on the SB-CGC, which completed in under 5 minutes and for less than five cents using spot instances.

The result was a list of 100 genes for each of four tumor subtypes reported in the top_genes_per_phenotype_matrix.txt (Table E in S8 Data). We compared the genes of these four top100 gene sets to each other and to the set of 208 genes from the original LUSC signatures (Table F in S8 Data). While 31 genes were shared in the top100 prioritized genes for the basal and classical subtypes, the secretory subtype was very distinct with no genes overlapping the other top100 lists. These four top100 lists and the top_genes_per_phenotype_matrix of Feature Prioritization were used as inputs to the Gene Set Characterization workflow described in the next section.

Resources

Signature Analysis Pipeline

KnowEnG Platform Tool [https://platform.knoweng.org/static/#/pipelines/signature_analysis]
Data Preparation Guidelines [https://github.com/KnowEnG/quickstart-demos/blob/master/pipeline_readmes/README-DataPrep.md]
Downloadable Results Description [https://github.com/KnowEnG/quickstart-demos/blob/master/pipeline_readmes/README-SA.md]

Seven Bridges Cancer Genomics Cloud

Public Tool [https://cgc.sbgenomics.com/public/apps#mepstein/knoweng-signature-analysis-public/]
Quickstart Guide [https://knoweng.org/wp-content/uploads/2018/07/SA_CGC_Quickstart.pdf]
Combined Workflow Tutorial [https://github.com/KnowEnG/KnowEnG_CWL/tree/master/CGC#running-the-gene-set-characterization-workflow]

Docker and GitHub Repositories

Signature Analysis Docker [https://hub.docker.com/r/knowengdev/signature_analysis_pipeline/]
Signature Analysis GitHub [https://github.com/KnowEnG/Signature_Analysis_Pipeline]
Data Cleanup Docker [https://hub.docker.com/r/knowengdev/data_cleanup_pipeline/]
Data Cleanup GitHub [https://github.com/KnowEnG/Data_Cleanup_Pipeline]
Pipeline Utilities Docker [https://hub.docker.com/r/knowengdev/base_image/]
Pipeline Utilities GitHub [https://github.com/KnowEnG/KnowEnG_Pipelines_Library]

Tables

Table B. Best Match Signatures of ESCC Samples.

Filtered output of Signature Analysis, mapping each of 79 ESCC samples from one of three 'cluster's to their 'best' match LUSC subtype signature by the maximum non-negative spearmen correlation of each subtype (columns C-F).

Table C. Best Match Signatures of LUSC Samples.

Results of Signature Analysis pipeline on 550 LUSC samples to find their 'best' match LUSC subtype signature by the maximum non-negative spearmen correlation of each subtype (columns B-E).

Table D. Signature Analysis of ESCA and LUSC Samples.

The TCGA samples from ESCA were divided into four “Sample Groups”, three groups of ESCC samples defined in [1], and one other for all remaining samples. We calculated the average non-negative correlation of these samples to each LUSC subtype signature and counted the number of samples with a best match to each subtype.

Table E. Top100 Gene Sets for Each Tumor Subtype from ESCC Samples.

Output of Feature (Gene) Prioritization, capturing top-100 gene lists for each tumor subtype based on the ESCC samples of that subtype compared to all other ESCC samples.

Table F. Overlap Between Top100 Gene Sets.

For the row and column gene set, shows the number of genes that are present in both. All gene sets are 100 genes except the LUSC-sig_genes set which is the 197 mapped genes from the original LUSC signatures.

Appendix C: KnowEnG Gene Set Characterization

Overview

The KnowEnG Gene Set Characterization pipeline performs the important role in the analysis of gene sets derived from omics data to identify the most related, previously curated pathways, functions, or experimentally annotated genes. This analysis provides researchers with context and information about their gene set of interest that can spur further hypotheses and investigations. The Gene Set Characterization pipeline is available in two modes: 1) a “knowledge-guided” mode that integrates analysis of prior knowledge gene annotations with

known gene-gene relationships from the KnowEnG Knowledge Network and 2) a "standard" mode that performs the most common statistical enrichment test.

Knowledge-Guided Gene Set Characterization

The knowledge-guided mode of the Gene Set Characterization pipeline is based on the method of Discriminative Random Walk With Restart (DRaWR) [3]. The fundamental idea of this analysis is that curated gene set annotations are incomplete, biased, and noisy. By integrating these annotations with additional knowledge about characterized relationships between genes and by calculating network-based distances between gene sets, we can find relevant and informative relationships that would not be captured by standard gene set overlap/enrichment. The KnowEnG Gene Set Characterization allows users to run this network-guided analysis on their gene set with many different types of gene interaction networks.

Standard Sample Gene Set Characterization

The KnowEnG Gene Set Characterization pipeline supports the standard statistical overlap test frequently used in gene enrichment analysis. In this mode, the user has access to the extensive collection of gene set annotations in the KnowEnG platform but does not make use of the gene-gene interaction networks from the Knowledge Network.

User Inputs

The primary input of the Gene Set Characterization pipeline is one or more gene sets. This can be provided to the KnowEnG platform in either of two ways:

1. For a single gene set, a list of gene symbols can be pasted into the platform with each gene symbol on its own line.
2. For multiple user gene sets, a "spreadsheet" of gene set membership can be uploaded with genes for rows, separate user gene sets as columns, and 0/1 indicator values.

For the knowledge-guided mode of Gene Set Characterization, the indicator values of the spreadsheet matrix can be substituted with non-negative "importance" values of the gene's membership to the gene set. For example, the absolute value of the differential expression (DE) fold change or the negative log of DE significance p-value can be used as importance scores.

More information about the formatting of input files can be found at our data preparation resource [https://github.com/KnowEnG/quickstart-demos/blob/master/pipeline_readmes/README-DataPrep.md].

User Parameters

There are a number of parameters that the user must select to run the Gene Set Characterization Pipeline.

Global Parameters

All modes of Gene Set Characterization require the user to select the

- [species] - for the user gene sets out of the twenty species in the Knowledge Network
- [public_collection] - gene sets to compare their user submitted gene sets to. The public collections available in KnowEnG, for example, include Gene Ontology, Protein Domain

Family annotations, GEO expression gene sets. The platform allows the users to select any number of these collections for simultaneous analysis.

Knowledge-Guided Only Parameters

In the prior knowledge guided mode, the user must specify the

- [interaction_network] - the gene-gene network available in the Knowledge Network for the selected species to use to create connections between the annotated genes
- [network_percentage] - the extent of influence the interaction network has in contributing to the ranking of annotation gene sets.

Data Preprocessing

Once the user selects the Gene Set Characterization inputs and parameters, a simple preprocessing step occurs before the main algorithm. If there are any missing or non-positive values, then the pipeline halts and produces a failure message. The input gene names and identifiers are first mapped to stable Ensembl identifiers of the appropriate [species] using the KN_Mapper tool (see Appendix E in S1 File) and the Redis database of gene aliases that accompanies the current Knowledge Network build. Unmapped rows (either missing or ambiguous mappings) are dropped along with the rows that contain duplicated mapped gene identifier. If any negative values are present, the pipeline will return an error message. If the user provided their input gene set as a pasted gene list, then the platform converts this list into a single column spreadsheet format. This spreadsheet will have one row for each Ensembl protein coding gene with a 1 for each gene that is in the user pasted gene list and a zero elsewhere.

Description of Algorithm

Standard Gene Set Characterization

The KnowEnG Platform provides users with the capability to run standard statistical enrichment of their submitted gene set(s) with the many annotation gene sets of the [public_collection]. This statistical test is performed with the one-sided Fisher's exact test [https://docs.scipy.org/doc/scipy-0.14.0/reference/generated/scipy.stats.fisher_exact.html at its core. For each user gene set and public collection gene set, the test is performed and the significance of the overlap p-value is returned. The gene universe for the test is defined as the intersection of the user's gene universe and the specific public collection's gene universe.

Knowledge-Guided Clustering

The knowledge-guided mode of the Gene Set Characterization pipeline implements the DRaWR algorithm in [3] and a more detailed description can be found there. The first step of this approach is to combine the [public_collection] gene annotations with the gene-gene [interaction_network] to construct a single heterogeneous network with both gene and annotation nodes and annotation-gene edges from the [public_collection] data and gene-gene edges from the [interaction_network]. The weights on these edges are then normalized for each of the two different edge types separately. A "baseline" random walk with restart (described in more detail in Appendix C in S2 File) is performed with all genes providing the restart node set and the [network percentage] providing the contribution of the heterogeneous network edges

(Figure A in S4 File). For each user gene set "query", a second random walk is performed using only the gene nodes of that gene set as the RWR restart set. If "importance" scores were provided in the submitted spreadsheet, the probability of returning to a gene of the restart set is proportional to its normalized importance score. Finally, the difference between the converged node state vector value of the "query" RWR and the "baseline" RWR is calculated, and the annotation nodes with the greatest difference are returned. These high-ranking annotation nodes are, according to the RWR guilt-by-association process, unusually well connected to the user gene set relative to their overall connection to genes in the network.

Pipeline Outputs

KnowEnG Platform Interface

Running the KnowEnG Gene Set Characterization (GSC) Pipeline in the KnowEnG Platform will produce results that can be viewed interactively. The GSC visualization displays the association score between user gene sets and the public gene sets calculated selected during pipeline run. This score is displayed in a heatmap view that can be manipulated using various sorting functions to help users visually group the strongest associations. The tool also allows users to drill into each cell for information about the degree of overlap and size and function of the public set. A hierarchical list of collection membership is also provided. Here the user can drill down and easily explore membership, as well as filter the heatmap view by toggling the display of the public gene sets individually or by category.

Downloadable Files

The primary downloadable file of the Gene Set Characterization pipeline is the ranking of [public_collection] annotation terms for their relatedness to the user-submitted gene set(s) along with the corresponding scores from the appropriate analysis mode. *In knowledge-guided mode, additional files containing metadata about the [interaction_network], data preprocessing, and pipeline run are also provided.* More information about the outputs of the pipeline and their structure can be found at [https://github.com/KnowEnG/quickstart-demos/blob/master/pipeline_readmes/README-GSC.md].

Methods

For each of the four top-100 gene lists that were derived from the subtype annotation of the ESCC samples (see Appendix B in S4 File and Table E in S8 Data), we performed the standard and the knowledge-guided mode of the Gene Set Characterization Workflow in the SB-CGC [https://cgc.sbgenomics.com/public/apps#workflow/mepstein/knoweng-genesetcharacterization-public/gene-set-characterization]. The input gene set spreadsheet for this step is the top_gene_per_phenotype_matrix.txt from the previous workflow task runs. Both runs were done using the taxonomy identifier for humans (9606) with the 'enrichr_pathway' public gene set collection from the Knowledge Network which is from the Enrichr [4] gene set resource and their extracted pathways from WikiPathways [5] and NCI Pathway Interaction Database [6] [https://s3.amazonaws.com/KnowNets/KN-20rep-1706/userKN-20rep-1706/Property/9606/enrichr_pathway/9606.enrichr_pathway.edge]. This collection had 30,214 gene annotations for 646 pathways, 437 from WikiPathways and 209 from NCI. For the knowledge-guided mode run, we selected the 469,784 edge, 15,999 node, HumanNet [7]

Integrated gene-gene [interaction_network] [https://s3.amazonaws.com/KnowNets/KN-20rep-1706/userKN-20rep-1706/Gene/9606/hn_IntNet/9606.hn_IntNet.edge] and a [network_percentage] of 50%. Each workflow run took under 5 minutes to run and cost less than five cents using spot instances.

The gsc_results.txt results of these two runs were downloaded from the SB-CGC and compiled to compare the top ranking annotation genes sets for each tumor subtype between the standard, 'Fisher', and the knowledge-guided, 'DRaWR' mode of the Gene Set Characterization workflow. Table G in S8 Data shows the top 10 pathway annotations for each subtype for both modes. We found that the two different modes frequently agree in their top 10 returned annotation gene sets. Thirteen pathway-subtype associations ranked in the top 10 results for both modes (Table H in S8 Data). Six of these 15 were supported by existing literature relating the pathways to squamous cell cancer studies and outcomes [8-11]. We were also interested in examining the pathway annotations that only were discovered by the knowledge-guided method. Twelve pathway-subtype associations were in the top 10 for 'DRaWR' mode and not in the top 25 for 'Fisher' mode (Table I in S8 Data). Among these, seven associations were supported by literature that related the pathway to esophageal or squamous cell cancers [12-18]. Five pathway-subtype associations were only in the top 10 for the 'Fisher' mode and not in the top 25 for the knowledge-guided run.

Resources

Gene Set Characterization Pipeline

KnowEnG Platform Tool [https://platform.knoweng.org/static/#/pipelines/gene_set_characterization]
Quickstart Guide [https://knoweng.org/wp-content/uploads/2017/08/GSC_Quickstart.pdf]
YouTube Tutorial [https://www.youtube.com/watch?v=nP4wtVZOY3E]
Data Preparation Guidelines [https://github.com/KnowEnG/quickstart-demos/blob/master/pipeline_readmes/README-DataPrep.md]
Downloadable Results Description [https://github.com/KnowEnG/quickstart-demos/blob/master/pipeline_readmes/README-GSC.md]

Seven Bridges Cancer Genomics Cloud

Public Tool [https://cgc.sbgenomics.com/public/apps/#mepstein/knoweng-genesetcharacterization-public/]
Quickstart Guide [https://knoweng.org/wp-content/uploads/2017/12/GSC_CGC_Quickstart.pdf]
Combined Workflow Tutorial [https://github.com/KnowEnG/KnowEnG_CWL/tree/master/CGC#running-the-signature-analysis-workflow]

Docker and GitHub Repositories

Gene Set Characterization Docker [https://hub.docker.com/r/knowengdev/geneset_characterization_pipeline/]
Gene Set Characterization GitHub [https://github.com/KnowEnG/GeneSet_Characterization_Pipeline]

Data Cleanup Docker [https://hub.docker.com/r/knowengdev/data_cleanup_pipeline/]
Data Cleanup GitHub [https://github.com/KnowEnG/Data_Cleanup_Pipeline]
Pipeline Utilities Docker [https://hub.docker.com/r/knowengdev/base_image/]
Pipeline Utilities GitHub [https://github.com/KnowEnG/KnowEnG_Pipelines_Library]

Figures

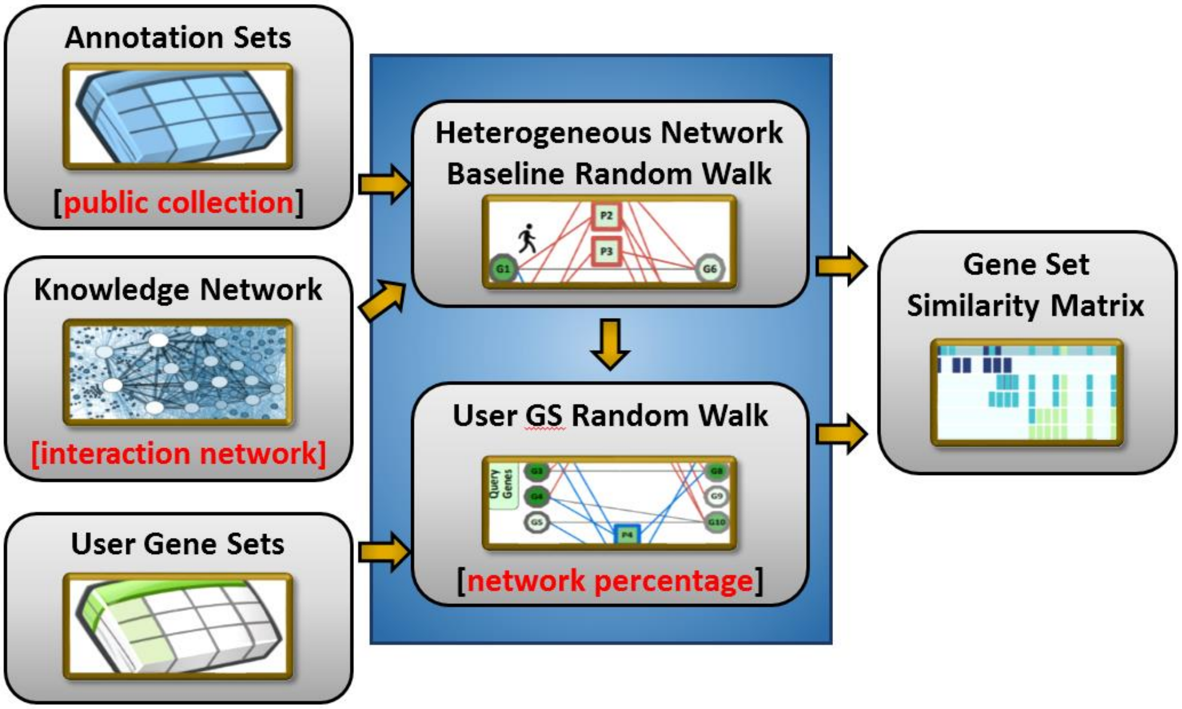

Figure A. Overview of Knowledge-Guided Gene Set Characterization.
A heterogeneous network is built from the selected gene-gene [interaction_network] and the annotation gene sets of the [public_collection]. A "baseline" random walk is allowed to converge using the [network percentage] and all genes as equal probability restarts. For each submitted user gene set, a random walk that only restarts the the users genes is performed and annotation set nodes are evaluated by their increase from their baseline.

Tables

Table G. Comparison of Results on ESCC Subtype Gene Sets.
Shows the ranking of pathway annotation terms for the four top-100 differentially expressed gene lists for each of the four subtypes from the ESCC tumor samples. Each row represents a "Pathway", "Tumor Subtype" association identified in the top 10 by either the standard, "Fisher", mode or knowledge-guided, "DRaWR", mode of the Gene Set Characterization pipeline. The "Source" and "Size" of the pathway annotation gene set provided, as well as the annotation gene set rank for both methods. The "Fisher_pval" significance of the enrichment of the pathway and tumor subtype gene list is also provided. The "Category" shows whether the association is in the top 10 for only one or both modes.

Table H. Shared Results on ESCC Subtype Gene Sets.

Shows the ranking of pathway annotation terms for the four top-100 differentially expressed gene lists for each of the four subtypes from the ESCC tumor samples. Each row represents a "Pathway"-"Tumor Subtype" association identified in the top 10 by both the standard, "Fisher", mode or knowledge-guided, "DRaWR", mode of the Gene Set Characterization pipeline. The "Source" and "Size" of the pathway annotation gene set provided, as well as the annotation gene set rank for both methods. The "Fisher_pval" significance of the enrichment of the pathway and tumor subtype gene list is also provided.

Table I. DRaWR Specific Results on ESCC Subtype Gene Sets.

Shows the ranking of pathway annotation terms for the four top-100 differentially expressed gene lists for each of the four subtypes from the ESCC tumor samples. Each row represents a "Pathway"-"Tumor Subtype" association identified in the top 10 by the knowledge-guided, "DRaWR", mode and not the standard, "Fisher", mode of the Gene Set Characterization pipeline. The "Source" and "Size" of the pathway annotation gene set provided, as well as the annotation gene set rank for both methods. The "Fisher_pval" significance of the enrichment of the pathway and tumor subtype gene list is also provided.

Appendix D: Consistency Analysis of DRaWR

Overview

Gene set characterization is the task of associating pathway or functional annotations with user provided gene sets. In KnowEnG, the Gene Set Characterization (GSC) pipeline offers a 'standard' mode of gene set enrichment tests using the hypergeometric test and implemented with the one-sided Fisher's exact test (Fisher). The knowledge-guided mode of the GSC pipeline (see Appendix in S4 File) is based on the Discriminative Random Walks with Restart algorithm [3], DRaWR. The fundamental idea of DRaWR is that curated pathway and functional annotations gene sets are incomplete, biased, and noisy. By integrating these annotations with additional knowledge about characterized relationships between genes and by calculating network-based distances between gene sets, we can find relevant and informative relationships that would not be captured by standard gene set overlap/enrichment.

We conducted a few additional analyses to explore the extent to which this fundamental idea is validated in the setting of associating Gene Ontology [19] (GO) annotation terms with disease based user gene sets. We found that DRaWR provided a complementary benefit for input disease gene sets that are largely incomplete and biased and is more robust to experimentally noisy input sets, although at the potential sacrifice of specificity.

Results

Recovering Annotations with Biased User Gene Sets

In our first set of experiments, we downloaded two databases of disease gene sets, dbGaP [20] and DisGeNet [21]. For many disease phenotypes, dbGaP (database of Genotypes and

Phenotypes) has performed extensive genome-wide association studies (GWAS) and has produced the genomic variants that correlate with the disease and the related set of nearby disease genes. DisGeNet curates a much larger collection of gene-disease associations by integrating information from many curated databases as well as from text mined associations extracted from MEDLINE (http://www.disgenet.org/dbinfo). In this set of experiments, we will perform gene set characterization to find Gene Ontology terms associated with the disease gene sets of dbGaP and DisGeNet. Since the gene sets for DisGeNet are assumed to be more complete (since they use multiple data types and sources), the top ranked GO terms associated with each DisGeNet disease gene set using the standard 'Fisher' enrichment test will be considered the ground truth in our experiments. We will call these ground truth sets of GO terms of each disease, 'Truth.Fisher.DisGeNet'.

The data for our experiments were downloaded from the KnowEnG Knowledge Network (see Appendix E in S1 File). Of the 16,558 Gene Ontology annotation terms for *Homo sapiens*, with 266,358 annotations covering 19,512 genes, we only consider 3,586 GO terms with at least 10 and no more than 2000 annotated genes. The dbGaP dataset contained 12,424 annotations for 344 disease phenotype gene sets covering 5,530 genes. The DisGeNet dataset has 429,036 annotations for 15,093 MESH disease terms covering 17,374 genes. We manually mapped (Table J in S8 Data) 245 dbGaP disease phenotypes to the MESH disease terms of DisGeNet (21 terms could not be mapped and 79 dbGaP terms with fewer than 9 annotated genes were discarded).

For these 245 disease terms, we ran three GSC tests and compared the top ranked 1% of the 3586 selected Gene Ontology terms for each run. The first run, previously described 'Truth.Fisher.DisGeNet', was treated as the ground truth since the Fisher method is well established and DisGeNet is a more comprehensive database. We also set up two additional runs for the two different GSC methods, which used the less complete, GWAS-only disease gene sets from dbGaP. We called the resulting top GO terms from these runs 'Fisher.dbGaP' and 'DRaWR.dbGaP'. The DRaWR run was conducted with the Blastp [22] Protein Sequence Similarity knowledge network (https://github.com/KnowEnG/KN_Fetcher/blob/master/Contents.md#homo-sapiens) as the 'interaction network' and with 0.5 as the 'network_percentage' which controls the influence the knowledge network has on the results. We then check the significance of the overlap of these two run GO term lists with the ground truth GO term list and calculate the difference between the log10 of their p-values as our score. A positive score means that DRaWR was better at recovering the ground truth GO terms than Fisher when using the corresponding dbGaP gene set as an input, and a negative score means that Fisher was better (Table K in S8 Data).

Even though this experimental setup likely is biased to favor the 'Fisher.dbGaP' method over the 'DRaWR.dbGaP' approach because the ground truth is based on the Fisher method, we actually find that the top GO terms of 'DRaWR.dbGaP' is more consistent with 'Truth.Fisher.DisGeNet' for 108 of our disease terms, compared to more consistent for the 'Fisher.dbGaP' test in 65 disease terms. (71 disease term showed no difference in the two approaches for recovering the ground truth GO terms). When we look at the disease terms that

performed better with DRaWR, we find that generally these disease gene sets have an unusually low number of dbGaP genes compared to the number of DisGeNet genes (Table L in S8 Data). We also see that for most of these diseases, the average of the negative log10 p-values is significant for the 'DRaWR.dbGaP' to ground truth comparison, but not the 'Fisher.dbGaP' comparison. For the disease gene sets where the Fisher methods was better able to recover the ground truth GO terms than the DRaWR method, we typically see that both methods were doing well (average negative log10 p-value significant), but the magnitude of the significance of the GO term overlap with the Fisher method was very large. We also see this tends to happen when the original dbGaP test set is unusually high or the DisGeNet truth gene set is unusually small. It is likely in these cases that the DisGeNet truth and dbGaP test gene sets are nearly identical and thus not surprising that performing the Fisher enrichment on both returns extremely similar top GO terms. Overall, these analyses show that DRaWR might outperform Fisher in recovering the true functional annotations of a when the input gene set is incomplete or suffers from a particular bias (e.g. the experimental or literature research method is costly and only applied to a subset of all possible genes).

Recovering Annotations with Partial User Gene Sets

In the next series of experiments, we wanted to quantify how the two modes of GSC compare at producing the correct functional annotations when only a small fraction of the full gene set is provided. To set up this experiment, we again treat the 'Truth.Fisher.DisGeNet' set of top 1% of GO annotations for each disease as the ground truth. However, rather than using test gene sets from dbGaP, we subsample from the full DisGeNet disease gene sets. For this analysis, we focus on 1299 DisGeNet disease terms that were annotated with between 50 and 5000 genes. We produced the 'DRaWR.DisGeNet.Full' top GO term for each disease by running DRaWR on the disease gene list. We then sampled the full list 20 times, taking either 20% of the full list or a maximum of 50 genes each time. Each of these twenty samples were sent to GSC to produce twenty top 1% GO term lists for each mode. As before, the significance of the enrichment of the ground truth list and each test list was computed and returned as the negative log10 p-value. These 'nlog10p' values were then averaged for the twenty runs of each mode, 'DRaWR.DisGeNet.Sampled' and Fisher.DisGeNet.Sampled'. These two averages are subtracted to capture the orders of magnitude difference in the significance ('Diff Score') of the ability of the two methods to recover the ground truth top GO annotations (Table M in S8 Data).

We find when running the above method that DRaWR excels at returning the correct top GO terms compared to Fisher for large DisGeNet disease sets when only a very small percentage of the gene set was provided to the algorithm. We note in this experiment, for 78% of the disease gene sets, the "Fisher.DisGeNet.Sampled" results were more similar (Diff Score < -1) to the the 'Truth.Fisher.DisGeNet' than the 'DRaWR.DisGeNet.Sampled' results (Table N in S8 Data). This again is expected by the ground truth is provided with the Fisher method. However, we note that 'DRaWR.DisGeNet.Sampled' still showed significant improvement (Diff Score > 1) for 8% of all diseases. These improved cases were enriched with especially large DisGeNet disease gene sets where sampling only 50 genes was especially harsh (average less than 10%). In these cases, the Fisher method performed much worse than average, while the DRaWR method was still able to return the GO terms of the 'Truth.Fisher.DisGeNet' at or above

its average. Even when very few genes are provided, the knowledge-guided analysis mode is able to use the neighbors in the interaction network to fill in the blanks find the correct functional annotations, whereas standard methods are less suited for this task.

Tables

Table J. dbGaP and DisGeNet Disease Gene Sets Mapping.

Shows the term names and number of genes in the mapped pairs of disease sets from dbGaP and DisGeNet.

Table K. GSC Mode Comparison with dbGaP Test Sets.

For each row, shows the 'Disease MESH term' associated for the disease of the row, as well as the number of 'dbGaP genes' in the test gene set and the number of 'DisGeNet' genes in the gene set used to determine the ground truth of associated GO terms. We compare the top 1% of GO terms from the ground truth to the top 1% (about 35) GO terms from running our knowledge-guided GSC with DRaWR on the dbGaP genes ('DRaWR.dbGaP'). This produces a number of overlapping GO terms, the significance p-value for that overlap, and the negative log10 version of that p-value (columns D-F). We repeat the process by running the standard mode of GCS on the same inputs ('Fisher.dbGaP') and summarizing the number of top GO terms that match the ground truth (columns G-H). Finally, we subtract our negative log10 p-values to produce our 'Score' for comparing the two GSC modes, which shows the orders of magnitude improvement of the enrichment with the truth significance of one mode vs the other (positive scores for improvement with DRaWR). We also produce scoring bins, 'Sc Bin' by powers of two.

Table L. Summary of GSC Mode Comparison with dbGaP.

For each power of two bin of our comparison score (which shows the orders of magnitude change between the 'DRaWR.dbGaP' and 'Fisher.dbGaP' runs enrichment with the 'Truth.Fisher.DisGeNet' top GO terms), we show the number of our disease term with comparison scores in that bin, 'Num Diseases'. We show the average number of dbGaP and DisGeNet genes in those diseases (columns C and D). We also show that average negative log10 p-values of the enrichment of the top GO terms of the two runs, 'DRaWR.dbGaP' and 'Fisher.dbGaP', for the top GO terms of the ground truth (columns E and F).

Table M. GSC Mode Comparison with Subset Test Sets.

For the disease that each row represents, we sample twenty time the 'Sample Size' number of genes from the full number of 'DisGeNet Genes' and calculate the 'Sample Perc' percentage. The top 1% of GO terms returned from running Fisher on the full set is kept as the ground truth ('Truth.Fisher.DisGeNet') and that is compared to the returned top GO terms from 1) the full set using BlastP homology knowledge-guided GSC, 'DRaWR.DisGeNet.Full', 2) the twenty samples also using the same DRaWR settings, 'DRaWR.DisGeNet.Sampled', and 3) the twenty samples using the standard enrichment, 'Fisher.DisGeNet.Sampled'. The negative log10 p-value of the enrichment between the truth and the GO list of 1) is returned and the average of those twenty

values are returned for 2) and 3) (columns F-H). Finally, to compare how the two modes performed on the sampled test sets, we subtract their average values, 'Diff Score'.

Table N. Summary of GSC Mode Comparison with Subsets.

Each row in Table M is assigned to one of three groups by their 'Diff Score' which captures whether the 'Fisher.DisGeNet.Sampled' or 'DRaWR.DisGeNet.Sampled' results are more similar to the ground truth of 'Truth.Fisher.DisGeNet'. The row is called DRaWR Better' if the 'Diff Score'>1, 'Fisher Better' if the 'Diff Score'<-1, and 'No Signif Diff' otherwise. The table below shows the count of the rows for each of the three categories as well as the average values for the corresponding columns in Table M.

References

1. Cancer Genome Atlas Research N, Analysis Working Group: Asan U, Agency BCC, Brigham, Women's H, Broad I, et al. Integrated genomic characterization of oesophageal carcinoma. Nature. 2017;541(7636):169-75. doi: 10.1038/nature20805. PubMed PMID: 28052061; PubMed Central PMCID: PMCPMC5651175.
2. Wilkerson MD, Yin X, Hoadley KA, Liu Y, Hayward MC, Cabanski CR, et al. Lung squamous cell carcinoma mRNA expression subtypes are reproducible, clinically important, and correspond to normal cell types. Clin Cancer Res. 2010;16(19):4864-75. doi: 10.1158/1078-0432.CCR-10-0199. PubMed PMID: 20643781; PubMed Central PMCID: PMCPMC2953768.
3. Blatti C, Sinha S. Characterizing gene sets using discriminative random walks with restart on heterogeneous biological networks. Bioinformatics. 2016;32(14):2167-75. doi: 10.1093/bioinformatics/btw151. PubMed PMID: 27153592; PubMed Central PMCID: PMCPMC4937193.
4. Chen EY, Tan CM, Kou Y, Duan Q, Wang Z, Meirelles GV, et al. Enrichr: interactive and collaborative HTML5 gene list enrichment analysis tool. BMC Bioinformatics. 2013;14:128. doi: 10.1186/1471-2105-14-128. PubMed PMID: 23586463; PubMed Central PMCID: PMCPMC3637064.
5. Slenter DN, Kutmon M, Hanspers K, Riutta A, Windsor J, Nunes N, et al. WikiPathways: a multifaceted pathway database bridging metabolomics to other omics research. Nucleic Acids Res. 2018;46(D1):D661-D7. doi: 10.1093/nar/gkx1064. PubMed PMID: 29136241; PubMed Central PMCID: PMCPMC5753270.
6. Schaefer CF, Anthony K, Krupa S, Buchoff J, Day M, Hannay T, et al. PID: the Pathway Interaction Database. Nucleic Acids Res. 2009;37(Database issue):D674-9. doi: 10.1093/nar/gkn653. PubMed PMID: 18832364; PubMed Central PMCID: PMCPMC2686461.
7. Lee I, Blom UM, Wang PI, Shim JE, Marcotte EM. Prioritizing candidate disease genes by network-based boosting of genome-wide association data. Genome Res. 2011;21(7):1109-21. doi: 10.1101/gr.118992.110. PubMed PMID: 21536720; PubMed Central PMCID: PMCPMC3129253.
8. Zhang J, Jiao Q, Kong L, Yu J, Fang A, Li M, et al. Nrf2 and Keap1 abnormalities in esophageal squamous cell carcinoma and association with the effect of chemoradiotherapy. Thorac Cancer. 2018;9(6):726-35. doi: 10.1111/1759-7714.12640. PubMed PMID: 29675925; PubMed Central PMCID: PMCPMC5983206.
9. Peng L, Linghu R, Chen D, Yang J, Kou X, Wang XZ, et al. Inhibition of glutathione metabolism attenuates esophageal cancer progression. Exp Mol Med. 2017;49(4):e318. doi: 10.1038/emm.2017.15. PubMed PMID: 28428633; PubMed Central PMCID: PMCPMC6130218.
10. Schmelzle M, Dizdar L, Matthaei H, Baldus SE, Wolters J, Lindenlauf N, et al. Esophageal cancer proliferation is mediated by cytochrome P450 2C9 (CYP2C9). Prostaglandins Other Lipid Mediat. 2011;94(1-2):25-33. doi: 10.1016/j.prostaglandins.2010.12.001. PubMed PMID: 21167292.
11. Szumilo J, Burdan F, Zinkiewicz K, Dudka J, Klepacz R, Dabrowski A, et al. Expression of syndecan-1 and cathepsins D and K in advanced esophageal squamous cell carcinoma. Folia Histochem Cytobiol. 2009;47(4):571-8. doi: 10.2478/v10042-008-0012-8. PubMed PMID: 20430722.
12. Casazza A, Finisguerra V, Capparuccia L, Camperi A, Swiercz JM, Rizzolio S, et al. Sema3E-Plexin D1 signaling drives human cancer cell invasiveness and metastatic spreading in mice. J Clin Invest. 2010;120(8):2684-98. doi: 10.1172/JCI42118. PubMed PMID: 20664171; PubMed Central PMCID: PMCPMC2912191.
13. Chen D, Hu Q, Mao C, Jiao Z, Wang S, Yu L, et al. Increased IL-17-producing CD4(+) T cells in patients with esophageal cancer. Cell Immunol. 2012;272(2):166-74. doi: 10.1016/j.cellimm.2011.10.015. PubMed PMID: 22082565.
14. Liao YM, Kim C, Yen Y. Mammalian target of rapamycin and head and neck squamous cell carcinoma. Head Neck Oncol. 2011;3:22. doi: 10.1186/1758-3284-3-22. PubMed PMID: 21513566; PubMed Central PMCID: PMCPMC3108931.
15. Song L, Wang X, Feng Z. Overexpression of FOXM1 as a target for malignant progression of esophageal squamous cell carcinoma. Oncol Lett. 2018;15(4):5910-4. doi: 10.3892/ol.2018.8035. PubMed PMID: 29552222; PubMed Central PMCID: PMCPMC5840556.
16. Ebihara Y, Miyamoto M, Shichinohe T, Kawarada Y, Cho Y, Fukunaga A, et al. Over-expression of E2F-1 in esophageal squamous cell carcinoma correlates with tumor progression. Dis Esophagus. 2004;17(2):150-4. doi: 10.1111/j.1442-2050.2004.00393.x. PubMed PMID: 15230729.

17. Kiyosue T, Kawano S, Matsubara R, Goto Y, Hirano M, Jinno T, et al. Immunohistochemical location of the p75 neurotrophin receptor (p75NTR) in oral leukoplakia and oral squamous cell carcinoma. Int J Clin Oncol. 2013;18(1):154-63. doi: 10.1007/s10147-011-0358-4. PubMed PMID: 22170235.
18. Wang W, Wang R, Zhang Z, Li D, Yut Y. Enhanced PPAR-gamma expression may correlate with the development of Barrett's esophagus and esophageal adenocarcinoma. Oncol Res. 2011;19(3-4):141-7. PubMed PMID: 21473290.
19. Ashburner M, Ball CA, Blake JA, Botstein D, Butler H, Cherry JM, et al. Gene ontology: tool for the unification of biology. The Gene Ontology Consortium. Nat Genet. 2000;25(1):25-9. doi: 10.1038/75556. PubMed PMID: 10802651; PubMed Central PMCID: PMCPMC3037419.
20. Tryka KA, Hao L, Sturcke A, Jin Y, Wang ZY, Ziyabari L, et al. NCBI's Database of Genotypes and Phenotypes: dbGaP. Nucleic Acids Res. 2014;42(Database issue):D975-9. doi: 10.1093/nar/gkt1211. PubMed PMID: 24297256; PubMed Central PMCID: PMCPMC3965052.
21. Pinero J, Queralt-Rosinach N, Bravo A, Deu-Pons J, Bauer-Mehren A, Baron M, et al. DisGeNET: a discovery platform for the dynamical exploration of human diseases and their genes. Database. 2015;2015.
22. Altschul SF, Gish W, Miller W, Myers EW, Lipman DJ. Basic local alignment search tool. J Mol Biol. 1990;215(3):403-10. doi: 10.1016/S0022-2836(05)80360-2. PubMed PMID: 2231712.
