# Supplementary material for: Knowledge-guided analysis of "omics" data using the KnowEnG cloud platform
Source: PLoS Biol. 2020 Jan 23;18(1):e3000583. doi: 10.1371/journal.pbio.3000583 (PMC6977717; doi:10.1371/journal.pbio.3000583)
Supplement: Attachment — Submitted filename: Response to Reviewers.pdf [file pbio.3000583.s009.pdf]

October 15, 2019

Dear Dr. Roberts,

Thank you for your consideration of the revised version of our manuscript entitled “Knowledge-guided analysis of ‘omics’ data using the KnowEnG cloud platform”. We thank the reviewers for their thoughtful reading of our initial submission and for examining the contents of the platform and the accompanying supplemental materials. We appreciate their recognition that our system represents a modern platform that provides analytical functionality that would otherwise not be available to non-specialist researchers. We also are grateful for their constructive comments on our presentation of the platform in the original submission.

We have attempted to address the reviewers’ concerns, to the extent possible, by providing a point-by-point response to each comment along and by making several changes to the KnowEnG cloud platform and the manuscript. Specifically, we have modified the manuscript to clarify 1) the significance and flexibility of the functionalities provided by our platform, 2) the importance and broad applicability of its knowledge-guided analysis algorithms, and 3) how the chosen case studies demonstrate these principles. These main manuscript improvements required the creation of an additional main figure, numerous changes to the existing supplemental materials, as well as two new supplementary notes containing additional user guides and new analyses. To address reviewers’ concerns about the flexibility of the KnowEnG system and potential missing capabilities, we also added two new features to the platform that enable users to perform differential expression analysis on RNA-seq raw counts with edgeR as well as to upload customized networks of prior knowledge to integrate in their analysis runs. We believe the manuscript and the platform have been improved by incorporating the comments raised by the reviewers and the academic editor, and we hope that they consider this revision worthy of your journal.

Sincerely,

Saurabh Sinha

Professor and University Scholar,
Department of Computer Science,
co-Director, NIH Center of Excellence for Big Data to Knowledge,
University of Illinois at Urbana-Champaign
sinhas@illinois.edu
www.sinhalab.net

**RESPONSE TO REVIEWER #1**

We thank the reviewer for their positive comments about our work and are greatly encouraged by their assessment of KnowEnG as a "significant and very welcome development that has potential of helping with 'democratization' of analytical tools accessibility." The reviewer also commended the system's ability to help users with "complex analytical tasks" in "a few relatively straightforward steps", stating that our pipelines could "result in significant time saving for bioinformatics professionals" and "enables analyses that would otherwise not be accessible to non-specialists". The reviewer also found the Knowledge Network as a "potentially game changing aspect of the system" and suggested that "the network itself can become an important test-bed for novel computational algorithms leveraging the whole network". We sincerely thank the reviewer for recognizing the value of our work.

**RESPONSE TO REVIEWER #2**

**Comment:** There are parts of this paper that I like, such as its focus on FAIR principles and use of modern platforms for implementing its functionality. Overall though, I feel that the benefits of the platform are oversold and/or unsubstantiated, and the focus of the paper is too mixed to have a clear impact.

**Response:** We thank the reviewer for their appreciation of our "focus on FAIR principles and use of modern platforms" and apologize that they found some of the system's benefits unsubstantiated. We sincerely hope that our substantial reworking of the manuscript, following the reviewer's suggestions below, address these concerns.

**Comment**: the authors state it marks "a significant step towards realizing the 'FAIR' vision". I don't find the support in the paper for this statement particularly compelling. There is no comparison to other extremely widely used platforms such as Galaxy, GenomeSpace, Terra, or DNANexus (some are not even mentioned).

**Response:** The reviewer makes a fair point, and we apologize for the previously terse reference to the rich milieu of existing frameworks that we hope KnowEnG can be accepted into. We have now added an extensive discussion section comparing what we believe are the combination of features that make KnowEnG unique and complementary to existing tools. We have also acknowledged the tremendous role played by several popular frameworks in moving towards 'FAIR' software in genomics and clarify KnowEnG's place in the context of their contributions.

If we consider non-commercial platforms with web portals that offer multiple analytics functionalities for genomics data sets, some prominent examples that come to mind include Galaxy, GenomeSpace, cBioPortal, NCI Genomic Data Portal (GDC), FireCloud/Terra, DNANexus, GenePattern, GeneWeaver and GeneMANIA. However, as we explain below, none of these systems offers the *combination* of (i) advanced data mining algorithms that can incorporate gene networks in their analysis, (ii) ready availability of several knowledge-bases in the back-end, to enable such network-guided analysis, (iii) diverse downstream analysis tasks geared for user-provided omics spreadsheets, from any of 20 different species, (iv) adherence to FAIR principles and Cloud-based scalable implementations, as well as (v) an online interface for visualizing data , that KnowEnG offers.

*Galaxy* [1]: This widely adopted online portal connects to several external databases, and provides many analytical functionalities. It offers convenient 'wrappers' around popular external tools, mainly for NGS data analysis, and many other tasks such as metagenomics, phylogenetics, and sequence analysis, which are outside the scope of KnowEnG. However, Galaxy is not geared towards knowledge-guided machine learning analysis of users' omics data spreadsheets. Here, omics data spreadsheet refers to a matrix of 'genes x conditions' where each entry is a numeric measurement on the gene, including mutation counts, expression, or any other gene-level statistic. Galaxy's functions are useful in creating those spreadsheets, but the 'downstream' analyses offered by it are relatively basic, and do not include sample clustering, gene prioritization for numeric phenotypic scores, signature analysis, nor do they include the powerful spreadsheet visualization capability of KnowEnG.

*FireCloud* [2], now *Terra* (https://terra.bio/), is a platform that provides access to TCGA data, along with tools for characterizing/annotating individual genomes; its tools for cohort level analysis identify consistent patterns of cancer-related mutations and copy number variations. It also allows users to analyze their own data, but does not offer knowledge network-guided analysis, integration of knowledge-bases in those analyses, and has limited visualization capabilities. The free-to-use analyses available on DNANexus [3], a commercial platform providing cloud-based analysis and management of genomic data, are similarly distinct from our system in that they focus on the processing of sequencing

reads without tools or datasets for downstream, knowledge-guided analysis. It is unclear from the website of DNANexus to what extent any of these features would exist in the analysis suite offered to premium customers.

*cBioPortal* [4] is a web portal with an API for accessing and visualizing cancer data, while *NCI Genomic Data Commons (GDC)* [5] is a web portal for accessing harmonized cancer datasets, and its 'DAVE' tools offer visualizations and useful descriptive statistics. However, these data portals are mainly geared towards visualization and exploration of omics spreadsheets extracted from the data sets they host rather than users' spreadsheets, and also differ significantly from KnowEnG in terms of analytics tasks offered, including knowledge-guided tools. Moreover, *FireCloud, cBioPortal,* and *NCI GDC* are entirely focused on cancer data analysis. KnowEnG tools are meant for analysis of user-provided spreadsheets from any biological domain, in any of its 20 currently supported species, and as we explain below, they are of general value in any biological domain even though our case studies have been focused on cancer data analysis.

*GenePattern* [6] is a web-based portal that offers a number of 'modules' for genomics data analysis, similar to 'KnowEnG pipelines', with similar functions. However, these general-purpose machine learning tools do not fall in the genre of 'knowledge-guided analysis', and thus do not offer the option of exploiting prior knowledge. On the other hand, *GeneWeaver* [7] and *GeneMANIA* [8] are online portals for knowledge-guided analysis of user data, but are limited to gene set inputs (similar to our gene set characterization pipeline) and do not provide tools such as sample clustering and gene prioritization for spreadsheet analysis.

*GenomeSpace* [9] is a warehouse of virtual workflows ('recipes') that show users how to answer certain questions using several external tools and databases, and these diverse tools include some of the analytical tasks offered in KnowEnG, such as clustering and gene set characterization. However, *GenomeSpace* does not perform any analysis itself, and acts more as a 'data highway' for passing data from one tool to another. As such, it belongs to a fundamentally different genre of analysis frameworks and is dependent on external tools and computing platforms to ensure scalability of its workflows. Moreover, the tools 'hosted' by *GenomeSpace* do not include the capability of knowledge network-guided analysis other than the desktop tool *geWorkBench*.

*geWorkBench* [10]: This Java-based open-source platform provides standard tools for common tasks such as sample clustering, gene prioritization, and gene set enrichment, comparable to 'baseline' options in the corresponding KnowEnG pipelines. It also provides specialized systems transcriptomics tools, including ARACNe [11], MARINa [12], Viper [13], and MINDy [14], that analyze a gene expression spreadsheet and a network simultaneously. We thus consider *geWorkBench* to be closest in spirit to KnowEnG. However, there are key differences as well. *Firstly,* geWorkBench has to be installed on the user's machine, and its web version has a small subset of tools from its desktop version, reflecting the challenges of offering a centralized web portal to compute-intensive tools with complex inputs. KnowEnG pipelines, on the other hand, embrace the use of computationally intensive but often trivially parallelizable techniques such as bootstrap sampling, because of the scalability afforded by Cloud computing. *Secondly,* in geWorkBench tools, the network is typically used as a 'filter', while the KnowEnG algorithms typically use network relationships to augment the spreadsheet rather than constrain the search. This permits use of a far broader class of networks than in geWorkBench. *Thirdly,* the scope of KnowEnG's analytics goes beyond the systems transcriptomics tasks tackled by the network-guided methods of geWorkBench.

In summary, a careful comparison of the features and goals of some of the major contemporary analytical frameworks reveals that KnowEnG brings complementary capabilities to the user, either in terms of the actual analyses allowed (compared with *Galaxy*, *cBioPortal*, *NCI GDC*, *FireCloud*), in allowing

knowledge-guided analysis (compared with *GenePattern*), in focusing on spreadsheet data analysis (compare with *GeneMANIA*, *GeneWeaver*), or in providing a consolidated Cloud-based back-end for scalable computation (compared with *GenomeSpace*, *geWorkBench*). Importantly, KnowEnG also supports analysis for 20 species currently, making its scope broader than several of the above-mentioned tools that are restricted to human genomic analysis. This is a major feature since knowledge-guided analysis requires integration of prior knowledge-bases with tools in a species-specific manner.

To make this point clearer in the manuscript, we have added a slightly abbreviated version of the above comparisons to a subsection ("Comparison with existing frameworks") of the Discussion.

**Comment:** There is no discussion of how many users the platform has.

**Response:** We appreciate the concern the reviewer is raising here, but our intention is to use this publication as our first major announcement that the Knowledge Network datasets and interoperable knowledge-guided downstream analysis tools of the KnowEnG Platform are available and free to use. This first broadcast was targeted to the cancer research community, although we designed our system for much wider audiences. While we do not currently have hundreds of active users, we do have many users that were introduced to the KnowEnG platform directly by us. For example, for the last three sessions of the Computational Genomics Course [https://publish.illinois.edu/computational-genomics-course/], each offered to about 80 researchers at the University of Illinois and the Mayo Clinic, we dedicated an entire day to knowledge-guided analysis and taught participants how to use KnowEnG and other tools with sample datasets. Several times participants have adapted the KnowEnG analysis for their own research problems and have continued to use the tool outside of the course to generate hypotheses and insights about their own data. We have also introduced the tool to individual researchers and research labs who have been excited by the new approaches to analyze their data and who have continued to submit their own data analyses. We did not include these anecdotal user stories in the manuscript.

**Comment:** Statements like "its tools being applicable to any data set comprising gene-level measurements or scores for a collection of samples" are abstract and do not spell out why the tools are more valuable than those already available through other online platforms. Instead, there are three examples focused on cancer analysis, with only a reassurance that "The scope of KnowEnG analytics goes far beyond cancer analysis". It is possible that KnowENG does represent a significant leap as a general platform, but the manuscript does not support this claim adequately. Instead, it reads like a platform the authors developed informed by their research needs and now claim as relevant to other users without adequate evidence for this claim.

**Response:** We agree with the reviewer's point, and in response we have attempted to revise the manuscript making the two ideas raised more concrete and clear by adding: (i) the unique value of KnowEnG tools relative to other online platform tools, and (ii) specific examples outside of cancer datasets where these analyses are needed. We elaborate on these two points below.

(i) KnowEnG pipelines analyze spreadsheets that tabulate numeric information about each gene (row) in each sample (column). The information may come from a variety of sources, e.g., high throughput transcriptomics assays using various technologies, mutation counts at the gene level, copy number variations, etc. The analytical approaches do not make strong assumptions about the source of the data. The particular functionalities offered by KnowEnG, especially sample clustering, gene set characterization and gene prioritization, make it comparable to only a select few of the popular web-based tools today (see the new subsection of the Discussion mentioned above, "Comparison with existing frameworks"), and the option of performing these tasks in a manner that accounts for the Knowledge Network makes it unique relative to those tools as well.

To make the value of knowledge-guided analysis more clear to the reader, we have added sentences to the introduction that highlight some of the many ways in which this type of analysis is currently being used in the literature for various tasks including: i) clustering of samples into cancer subtypes [15-18], ii) finding markers and drivers of disease [19-24], iii) prediction of patient survival [25, 26] or cancer metastases [27], iv) characterization of experimental gene sets [28-32], and v) prediction of gene functions [33-35]. KnowEnG offers such knowledge-guided analysis methods as optional 'advanced' modes of its supported pipelines. It should also be noted while these methods are being used frequently in genomic data analysis, there are currently few online systems where they can be run. For the most part, only users who are able to install and run these tools on the command line have access to them. Even then, before they can start, the user often has to struggle with collecting and preparing the prior knowledge datasets into the acceptable formats that will likely vary by each tool.

(ii) KnowEnG pipelines can be used in a variety of scenarios that resemble the case studies outlined by us. To make this clear, we added the following paragraphs to a new subsection of the Discussion called "Applications to other biological domains".

The gene prioritization pipeline may be used in any scenario where a spreadsheet of gene-level measurements (expression levels, mutation counts, copy numbers, epigenomic measurements, etc.) are available on a collection of samples, along with a phenotypic score for each sample. For instance, this pipeline was used by Emad et al. [36] to identify genes whose basal expression in a cancer cell line is predictive of the cell line's response to a cytotoxic treatment. Similar analyses have been performed in other published studies [37], although without incorporating a knowledge network. Other examples of potential applications to gene prioritization for numeric phenotypes include identifying genes whose brain expression levels are predictive of pheromone response in honeybees [38], discovering genes predictive of growth rate in bacteria or yeast [39], and identifying gene families whose size (number of paralogs) in a species is correlated with a numeric score of that species, e.g., eusociality index in bees [40]. Indeed, the potential of this line of analysis is evidenced by the recent publication of a tool specifically for relating expression to traits ('TraitCorr' [41]) as an R package. The task of identifying differentially expressed genes between two conditions (binary phenotypes) can also be performed, in a knowledge-guided manner, using the gene prioritization pipeline. The high utility of this task needs no introduction, and many tools are available for it [42]. The unique value of the KnowEnG pipeline is that common statistical tests used for this task, e.g., t-test or EdgeR [43], can be combined with 'smoothing' of gene expression values based on the Knowledge Network as well as subjected to 'bootstrapping' for robustness. (We and others have already demonstrated the value of network-smoothing and bootstrapping in prior work on gene prioritization [36].) These additional features of the pipeline are well-supported by a cloud-based platform that offers easy scalability and pre-stored knowledge networks, thus avoiding the hassles of maintaining compute clusters and downloading large networks for a more traditional 'local computation' such as those using Bioconductor packages [44].

Clustering is a pervasive operation in bioinformatics and finds uses in many scenarios. Such clustering may be performed within KnowEnG for any of the 20 species supported by it, for any set of experimental conditions, and for any type of omics data that assign numeric measurements to genes, to reveal hidden groupings among the conditions. While the common tools for gene expression clustering focus on the task of clustering genes, the KnowEnG Sample Clustering pipeline is geared towards finding groups of samples/conditions that have similar expression profiles. This distinction is crucial to its use of a knowledge network to guide the clustering, lends it a complementary strength, and is expected to be of increasing utility in the future as the practice of profiling tissue samples from individuals grows more popular [45]. The most common uses of sample clustering are in identifying subgroups in cancer patients, based on transcriptomic as well as other omics data sets, e.g., identifying breast cancer subgroups from

copy number variations [46], colon cancer subgroups from gene expression data [47], refinement of breast cancer subtypes based on miRNA expression profiles [48], subtyping of different cancers from somatic mutation data [49] to name a few. Other uses of clustering to group samples include clustering of type 2 diabetes patients as well as obese and healthy subjects to find that T2D and obesity have similar expression profiles [50], grouping of brain transcriptomes of honey bee nurses and foragers of different ages to show that each behavioral group has similar profiles [51], clustering of Arabidopsis plants treated with plant activators [52], etc.

The Gene Set Characterization pipeline addresses one of the most commonly performed tasks in genomics analysis, which is sometimes referred to as 'gene set enrichment analysis', which may be performed using the GSEA tool [53] or through Hypergeometric tests. Studies that use this analytical operation are too numerous to list here, but its popularity is evidenced by the huge following that online tools such as DAVID [54] and Enrichr [55] have. We nevertheless included this pipeline in KnowEnG because it is a natural follow-up for the gene prioritization pipeline, and we expect users to make use of it every time they identify top genes associated with a phenotype. Moreover, the KnowEnG pipeline offers two complementary approaches to the above task – the popular approach based on Hypergeometric tests (as in DAVID) and a novel approach based on Random Walks with Restarts (RWR), which we have published previously [28] and whose unique value we further demonstrate in Supplementary Note SN11. The RWR-based method not only provides an alternative approach to identifying pathways, Gene Ontology terms, etc. most relevant to a given set of genes, it does so while accounting for gene-gene relationships encoded in a knowledge network, according unique value to the KnowEnG pipeline. We have also published the use of the RWR-based method to characterize gene sets arising out of a brain transcriptomic study of social behavior in three different species [56].

**Comment:** The second focus is that users can use KnowENG to reveal insights into their data. Perhaps this is my bias, but if I were to analyze an 'omics dataset I had generated, I would likely have a hypothesis in mind and care deeply about the underlying "knowledge graph" that was used to support clustering/prioritization/etc. For example, I may want to use a graph that only includes datasets in tissues relevant to my disease, or to exclude certain datasets I may not trust.

**Response**: We agree with the reviewer in that many users would hope to use a knowledge network/graph of their own choosing. We have heard the request for this feature several times, but the new feature was not ready at the time of the initial submission. However, we are happy to report that the first version of this feature is now available in the KnowEnG web platform and made possible by our 'Network Prepper' tool (more information can be found in Supplementary Note SN5). This tool allows users to transform their own gene-gene knowledge networks (e.g. tissue-specific interactions or co-expression networks derived from the data) into the format and internal stable identifiers necessary for compatibility with the KnowEnG knowledge-guided analysis pipelines. We have now mentioned the availability of this feature in the Discussion.

We also believe that many researchers face a point where they have profiled a collection of biological samples carefully selected to reflect their question, and then seek to perform a relatively broad statistical analysis of those data to identify or rank strong 'signals' emerging from the data. At this point, they may not have specific hypotheses in mind, e.g., the constitution of unknown subgroups among samples, specific genes that characterize those subgroups, or pathways involved in a biological process. They look to tools to perform this task for them, and ideally, they would then use their expertise to critically examine the signals identified by the tools and proceed to form and test specific hypotheses. It is in this intermediate step of exploratory analysis that KnowEnG pipelines can be especially useful, particularly for the ease of performing the exploration. Such researchers may not care as deeply about

or have access to the most appropriate prior knowledge network and will thus benefit from the several general options available by default in the KnowEnG platform.

**Comment:** Without the ability to tailor data/analysis to my hypothesis, the described workflows will only yield broad insights; e.g. "A log-rank test revealed highly significant distinction across the clusters in terms of survival probabilities (p-value 3.7E-33)". This is nice but leaves me unclear on what the next steps would be (are the results of a KnowENG analysis publishable without anything further? do they suggest an experiment?). Lines 165-174 seem to support this view:

**Response:** We agree with the reviewer about the 'broad' nature of insights revealed by many of the analyses in the case studies presented. Ideally, emergence of broad insights should be followed by closer examination of those insights, and to an extent, the workflows in the case studies are meant to reflect this process. For example, in case study 1, a sample clustering step (see Supplementary Methods SM3) reveals that the omics profiles are distinct and biologically meaningful (e.g., exhibit different survival characteristics in patients). This is followed up by additional analyses (detailed in Supplementary Note SM7) that use Gene Prioritization to be more specific and identify genes discriminative of those distinct groups and then finally Gene Set Characterization to help make sense of the list of genes thus revealed, in terms of pathways likely affected. We also wish to point out that one of the reasons we chose our case studies to mimic the analyses in two high profile publications [57, 58] is that the results of those analyses are clearly 'publishable', and the original papers often provide examples of the follow-on experiments suggested by the results (e.g., genes to over express or knock-down, pathways to target, etc.).

For an example of how the KnowEnG tools have been used to generate hypotheses, which led to a series of additional experiments, we point to our study using the knowledge-guided Gene Prioritization pipeline [36] (called ProGENI in its original manuscript). In this study of sensitivity of tumor cell lines to cytotoxic drug treatments, we found that analysis of basal mRNA expression data using the prior knowledge of protein-protein interactions improved our ability to recover genes who were known in the literature to modulate the responses to the administered drug. For top prioritized genes where no publications existed, we set up and conducted siRNA knockdown experiments, which confirmed that a high percentage of these putative modulators indeed had an effect on the sensitivity of cancer cell lines to treatment with the drug. The use of the knowledge-guided Gene Prioritization and the follow up experiments ultimately led to insights into the mechanisms of drug resistance and possible genes to target to overcome the phenomenon.

**Comment:** It is not surprising to me that these clusters would not match tumor types, since the KnowENG analysis was not designed to find tumor types (instead, its reliance on large-scale databases makes it unsurprising it clusters according to genes/pathways, since most databases will capture pathway information).

**Response:** In the indicated paragraph (Lines 165-174), we sought to point out how knowledge-guided sample clustering can provide researchers with patient subtypes that are potentially more interesting than a simple grouping of patients by tumor of origin. We demonstrated earlier in the manuscript that standard clustering approaches on very sparse mutation data frequently provide meaningless clusterings, which have no relation to survival outcome. Using the final multi-omics clustering Hoadley et al.[57] find clusters that also have a significant relationship to survival outcome, but those clusters are remarkably similar to just grouping the patients by tumor type. The knowledge-guided clustering of mutation data only (using our method or the pathway-based method in Hoadley et al.[57]) shows that patient subtypes exist that do not follow tumor type closely, but that are still predictive of survival outcome. We agree with the reviewer's point that these patient subtypes are also more likely to have mutations in genes in the particular pathways embedded in the prior knowledge. That is in fact one of

the goals of treating this sparse data with knowledge-guided analysis. In Supplementary Note SN7, we examine *which* pathways relate to *which* subtypes and highlight that although these subtypes are mixed in terms of tumor types, each knowledge-guided subtype relates to specific and often distinct pathways.

**Comment:** What if I (as in Hoadley et al) wanted to cluster by tumor type? Likely I would need to take much more care in defining my underlying analysis and prior data; can I do that via KnowENG?

**Response:** If a user wants to view patient data by tumor type, this functionality is available in our 'Spreadsheet Visualizer' tool. The Spreadsheet Visualizer offers users a quite simple way to upload and view the genomics data, the clinical data (which includes the tumor of origin), and the results of the various clustering approaches. In this visualization tool, the user can group the patient samples by any of the categorical clinical features (e.g. tumor type) or any of the Sample Clustering pipeline results. They can also get statistics about significant associations of these particular groups with each other and with any of the genomic features (see Figure 3E and more detail in Supplementary Note SN8). Within the Spreadsheet Visualizer, the Kaplan-Meier curves for the patient grouping (Fig 3B and 3D) can be plotted and the significance calculated.

It is also possible to more carefully define the data used in the analysis to reflect the user's own prior knowledge of a biological system. For example, in the "Clustering for patient stratification" subsection of Case Study 1 in the Results, we performed Sample Clustering on gene expression data from breast cancer patients only (more details in Supplementary Methods SM6). Additionally, rather than performing the clustering on all measured genes, we modified the gene expression spreadsheet to only contain a subset of 253 genes previously known to relate to the epithelial to mesenchymal transition, a process involved in tumor metastasis. Unsurprisingly, the subtypes we found using this more carefully designed dataset had a significant relationship to survival outcome. We have developed several tools for this sort of manipulation of omics spreadsheets and made them available to users through our JupyterHub server (see Supplemental Methods SM4). With these 'Spreadsheet Transformation' tools and the 'Network Prepper' tool for uploading custom prior knowledge networks, there are several ways in the KnowEnG system to produce specific inputs for more precise analysis.

**Comment:** What if I wanted to understand whether the KnowENG clustering was driven by one dataset, or "promiscuous" genes across the network that had a high weight in the analysis? Can I use KnowENG to conduct follow-up analyses?

**Response:** We thank the reviewer for these excellent questions and have a number of relevant responses. First, in the application of the Sample Clustering pipeline to mutation data, there are two outputs that are immediately available that could help an interested user resolve this question. First is the 'top_feature_by_cluster.txt' file that enumerates the top 100 genes per cluster by their 'network-smoothed' mutation scores. This score is an average of the stationary probabilities calculated on each sample by the random walk with restart algorithm on the knowledge network [15] (these probabilities roughly capture the proximity of each gene in the network to other genes with mutations in each sample (see Supplementary Methods SM3 for more detail)). The clustering pipeline produces the 'top_feature_by_cluster.txt' file because it can be directly passed to the Gene Set Characterization pipeline, which returns enriched pathways of those top genes for each cluster. The particular values for the network-smoothed mutation scores for each cluster are available in another file, 'feature_averages_by_cluster.txt', which the user is able to download and manipulate via other spreadsheet tools (e.g. Microsoft Excel).

A second option that often exists for the investigator is to run the Gene Prioritization pipeline in KnowEnG with the original input and the clustering results of Sample Clustering. This option is distinct from the previous case because the top genes returned by this method for each cluster will not

necessary be the highest scoring, but the most significantly different from the cluster to all others. A version of this process is demonstrated in Supplementary Note SN7. Both of these two options highlight the value of being able to chain together multiple pipelines of KnowEnG for deeper analysis.

While we are still developing network visualization tools for deployment inside the KnowEnG platform, a third option available to the user would be to explore the mutation scores mentioned above in the context of the Knowledge Network using external tools. The prior knowledge networks are easily downloadable [https://github.com/KnowEnG/KN_Fetcher/blob/master/Contents.md] and are compatible with popular tools such as Cytoscape [59] or NDex [60]. This option would help users understand if the most important genes from the knowledge-guided results are also hubs in the Knowledge Network. Finally, we point out that the Sample Clustering pipeline (like others in KnowEnG) comes with a 'bootstrapping' option, which repeats the clustering analysis many times each time using a different random subset of the gene features. If a few 'promiscuous' genes did exist that would adversely skew a single Sample Clustering result, they would not be consistently present across all repeated runs of the analysis, making the final clustering reported on the consensus results robust to their effect.

**Comment:** It is possible that KnowENG in fact addresses my comments above. But the manuscript as written does not make it clear that it does. The manuscript should either (a) focus on the value of a new FAIR platform, by comparing KnowENG to existing platforms (see above), supporting that many users have found it to be of value, and being more clear on the value added by its tools; or (b) focus on KnowENG as enabling researchers to conduct specific analyses/workflows, by giving evidence that it yields publishable insights, or at minimum suggests follow-up experiments/analyses.

**Response:** We hope that we have addressed the reviewer's comments above, and in doing so have argued successfully why we consider KnowEnG to be a valuable new FAIR platform (in comparison to existing tools), as well as how it enables researchers to conduct common types of analysis with publishable results and suggestions for follow-up experiments. We have, however, been unable to draw the focus on only one or the other of the two points above, since we believe them to be too intertwined to separate. As we detailed in the responses above, we have added a substantial subsection about related online frameworks and the unique strengths of KnowEnG (focus point 'a' above) to the Discussion section. Our 'Results' section is written in a manner more geared towards focus point 'b' above, i.e., to show examples of publishable insights and follow-up suggestions arising from the KnowEnG workflows. We have now modified the structure of the Results section to make this point clearer, with a new initial subsection ("Overview of three 'case studies'") and new schematic diagram (Fig 2) outlining what insights the case studies hope to demonstrate.

**RESPONSE TO REVIEWER #3**

**Comment:** Interpretation of the deluge of biomedical data, wether publicly available or user-generated, is at the basis for the majority of current research. Tools like KnowEng represent the link between researchers with a strictly "wet" background" and the multitude of tools that allow meaningful interpretation. We like the integrative approach of KnowEng and the fact that it is based on current and sensible software principles (i.e. interoperability, web-based access, etc).

**Response:** We are grateful to the reviewer for their favorable assessment of the motivations and overall design principles of KnowEnG.

**Comment:** If we were to evaluate this manuscript in terms of the concept it implements, our concerns would only be the fact that most bioinformatics analysis nowadays are made in the R framework, and we are not sure how this fits in the KnowEng framework. We routinely create custom scripts to perform very specific analysis steps, and if KnowEng does not allow for those to be integrated, it would affect the flexibility of the framework.

**Response:** We agree with the sentiment expressed by the reviewer that repositories of R packages such as Bioconductor [61] mean that a substantial number of bioinformatics analyses today are done in R. Although our current KnowEnG pipelines are all written in Python, the overall framework was designed in such a way that adding a new R based pipeline is as straightforward as adding additional Python ones. The key reason underlying this is that all pipelines are launched as separate, short running Docker [62] containers that perform the necessary computation. These Docker containers are unique to each pipeline, and therefore, can have different software, libraries, and source code installed inside. From the perspective of the KnowEnG platform, only the command string is needed to execute the analysis tool inside the running containers, the language that was used to implement the pipeline tool is irrelevant. This is also true for running our tools in the Seven Bridges Cancer Genomics Cloud (SB-CGC). The Common Workflow Language description that describes the pipeline's inputs, outputs, and executions steps does not care about the language used to write the tool since it knows the appropriate Docker container to run. To clearly describe the simple steps necessary for adding new pipelines to our public KnowEnG Platform server, our JupyterHub server, the SB-CGC platform, or our AWS CloudFormation template, we have written the new Supplementary Note SN12. Hopefully, following this first guide will help developers better understand what is required for new pipelines to be integrated into the KnowEnG framework. Additionally, the flexibility of the KnowEnG framework is now more fully discussed in a new subsection of the Discussion ("Flexibility of KnowEnG functionalities").

**Comment:** However, our main concern is related to the software engineering part of the software. For example, we looked at the feature prioritization pipeline. This step is, in our opinion, one of the most crucial parts of every pipeline that makes use of high-throughput data. For example, in transcriptomic experiments, when all (or most) genes/transcripts are quantified, the presence of noise is inevitable, and feature prioritization is absolutely mandatory for removing such noise and extract meaningful variables. KnowEng implements two very simple and outdated methods for the prioritization of important features. We do understand that these are very general methods and as such they will be appropriate (in that they do not make too many assumptions on the distribution of the data) for most datasets, but today, these would cut out the majority of the existing data. In particular, both Pearson correlation and Student's t-test would be inappropriate for RNA-Seq data due to the particular distribution of count data (Negative binomial), and it seems that KnowEng does not allow for using appropriate tools like EdgeR or DeSeq. Even for microarray data we would never use such measures, instead preferring moderated t-test (as in the Limma package, see Smith et al 2005) or SAM (Tusher et al, 2001).

**Response:**

This is an important concern and we appreciate the reviewer for raising it. As the reviewer notes, we made the decision to implement broadly applicable statistics that could be used by many communities rather than a handful of very specific models that work for certain communities or data types. This seemed like a practical first step, with the intention being that the FAIR-inspired framework would allow for the easy integration of highly specialized tool in later stages (see previous response and the new Supplementary Note SM12). Because we chose to implement our pipelines with simple statistics, our platform assumes that users of KnowEnG will perform the appropriate normalization on their data before uploading it to our platform. We consider such pre-processing as vitally important, but an 'upstream' task for which numerous platforms exist (Galaxy, Seven Bridges, FireCloud/Terra, etc.) and which are able to prepare the spreadsheets that KnowEnG analyzes. The fact that such normalization is often technology-dependent led us to not incorporate it into the more 'downstream' analyses offered by KnowEnG, and instead presume it to be performed prior.

In the case of prioritization with the Pearson correlation, it is intended more for situations where the researcher has access to a numeric score or label for each sample (the 'phenotype') and wishes to prioritize genes whose scores (typically, expression) correlate with the phenotype. This is different from the task of identifying differentially expressed genes, i.e., when the phenotype is binary (one group versus another). In this different scenario, the more common approach is to normalize expression data as appropriate for the tool and then find gene-phenotype correlations [37, 39, 41]. We also note that this approach with normalization and correlation was deployed successfully in the original ProGENI paper from which the Gene Prioritization pipeline derives its method and where we were able to experimentally validate several of the identified genes as modulators of drug resistance in cell lines.

Finally, in response to this and the previous comment by the reviewer, we decided to add edgeR [43] support for differential expression analysis to the standard mode of the Gene (Feature) Prioritization pipeline that does not use the knowledge network. Two primary reasons for this addition were i) because it is possible that some users might want to come to the KnowEnG platform with a spreadsheet of raw RNA-seq counts and ii) to demonstrate the ease in which R based analysis tools can be incorporated in the KnowEnG framework. This edgeR functionality supports examining raw counts with respect to binary or categorical phenotypes. In the case of multiple categories, differential expression values will be calculated separately for each category in a one vs all setting. When users launch this new option for the pipeline on their raw count data, edgeR uses the TMM method to normalize the data and the quasi-likelihood F-tests to calculate the significance of the differential expression. The R based edgeR tool runs in a Docker container with R, Bioconductor, and the package already installed and does not disrupt the structure of the Python-based KnowEnG platform (as noted in the previous response).

**Comment:** This only points to the main shortcoming of all bioinformatics pipeline frameworks: since it is impossibile to allow for all state of the art tools to be present in a single framework, the tool MUST be flexible enough for users to be able to easily plug in new blocks of the pipeline as needed. This ability needs to follow well established software engineering principles. If KnowEng supports such flexibility, this is never explained in the text nor in the supplementary information. We understand that PlosBio could be seen as more "bio" oriented, but it is a software tool we are talking about, and as such the software part has to be described in detail and justified.

**Response:** We agree with the reviewer and hope we have made a convincing argument to the ease of adapting new tools into the KnowEnG framework by adding the R based edgeR to the Gene Prioritization pipeline and the new 'Network Prepper' tool (that allows users to upload their own knowledge networks; see Supplementary Note SN5) to the platform during the time of the revision. We also very carefully laid out an overview of the steps required to integrate a new pipeline in the new

Supplementary Note SN12, which interested developers with their own analysis algorithms can use as a starting point. Currently, some manual development effort is required to integrate these new pipelines. However, our hope is that by building our framework on Docker and the Common Workflow Language, at some point the KnowEnG platform will be able to run knowledge-guided and standard analysis pipelines that are submitted by outside developers. We note that Case Study 3 clearly demonstrates that this is currently possible in the Seven Bridges Cancer Genomics Cloud using these same technologies.

**Comment:** The results presented in the manuscript are great, but they represent a small set of datasets analyzed. In order to accept this manuscript, we would need these aspects to be present and clear in the text.

**Response:** We thank the reviewer for all their comments and now present revised, clearer arguments about the generalizability of KnowEnG analysis in the Discussion section. We have added an extensive discussion of why we believe the pipelines we have chosen to support initially are like many analysis scenarios faced by genomic biologists (see subsection "Applications to other biological domains"). We have also added a comprehensive discussion comparing the KnowEnG software to other analysis frameworks (see subsection "Comparison with existing frameworks") and a discussion of the features of KnowEnG implementation that afford flexibility in its usage (see subsection "Flexibility of KnowEnG functionalities").

**RESPONSE TO ACADEMIC EDITOR**

**Comment:** I have been through the work, read the reviews and also had a look at the website. I too have major reservations about this paper. On the positive side, there has clearly been a lot of time and effort put into this platform and they have gone to considerable lengths to support users with the provision of tutorials etc. I too like the emphasis on cloud compute and FAIR principles, even if as reviewer 2 states they are invoked but it is not necessarily spelt out how they conform to them. The user interfaces also look pretty clean and useable/useful and KnowEnG may indeed be a useful new platform.

**Response:** We thank the academic editor for their positive comments about the efforts we have undertaken to support users with tutorials, emphasize cloud compute and FAIR principles, and create clean looking and useable/useful interfaces. To respond to reviewer 2's comment, we have now added to the Discussion text about how KnowEnG system adheres to FAIR principles in the context of alternative genomics software tools as well as by emphasizing the flexibility those principles enable in our framework**.**

**Comment:** On the negative side I share many of the reservations of reviewers 2, 3. I also did not like the way the resource was presented. Each functionality is described and then applied to a different 'use case' dataset and in each instance we were told that KnowEnG outperformed standard methods. Ultimately I found this a pretty unsatisfactory way to present the functionality of the tool as the reader is not really given enough information about each dataset, the insights generated are provided out of context and I found these sections to just provide a superficial overview of a dataset I was not invested in and therefore it ended up being a dull read.

**Response:**

We hope our responses to address the specific reservations of reviewers 2 and 3 also assuage some of the concerns of the editor. The analyses presented in this manuscript were selected to recapitulate findings from high impact publications likely familiar to the cancer and wider communities, so as to highlight how the KnowEnG pipelines can be easily applied to uncover broad insights from omics data sets. The case studies were designed to also illustrate how popular knowledge-guided analysis and multi-step analyses can lead to more specific hypotheses being generated (such as genes and pathways involved in a process) and can be performed seamlessly within KnowEnG. In order to provide more context to the cancer analysis case studies presented in the manuscript, we have added a subsection ("Overview of three 'case studies'") to the beginning of the Results that provides a high-level 'roadmap' of the section. With this new text, we try to explain for each analysis i) the purpose of including it in the demonstration of the capabilities of the KnowEnG system, ii) why it follows from the previous analyses in its case study, and iii) the type of high-level biological understanding that was achieved from running it. Along with these description in the subsection, we have included a new figure (Fig 2) that diagrams how the analyses in the case studies relate to each other through the reuse of the same pipeline components. Fig 2 explicitly describes the data inputs and outputs of each step, shows where knowledge-guided analysis is being deployed in each case study, and highlights the related manuscript figures and supplementary detail for each result. We hope this new overview better establishes the purpose and decisions guiding each case study, and therefore provides the context to better appreciate the results.

**Comment:** Most the methods behind this tool are published we are told, so why do we need to see them applied and justified here?

**Response:** The previous publications have presented the underlying algorithms, but did not present a software system and platform where those algorithms can be invoked easily and scalably, nor how richer analyses can be constructed by stringing together multiple algorithms. Our goal in this paper is to

present the online, cloud-based system that allows this, and to reach out to biologists with an invitation to use the system. This is why we selected two relatively recent and well-received publications [57, 58] in the cancer genomics community to motivate our case studies. We hope to convey the message that those analyses (and more), which clearly required an immense investment of software, hardware, and human resources, can be easily reproduced or approximated within the KnowEnG system.

**Comment:** If there is a new resource such as this my main interest in reading about it are, how do I access it, what data do I need to feed in from which analysis platforms, what analysis routines can I run on the data and how do I do this, what form do the results come back to me in and what advantages does it offer relative to other tools/platforms. At the moment, I don't get this from the paper in its current form.

**Response:** We thank the editor for their comment and have made several changes to the main manuscript to address these concerns.

1. Previously missing from the main manuscript were the URLs to access the different modalities of KnowEnG. These links were available in the corresponding supplemental texts, but we have now moved them to the Introduction so potential platform users will be able to start using our tools right away.
2. Also, the input and output data types were introduced separately for each pipeline with most of the detail embedded in the corresponding Supplementary Methods. In order to provide users with an overview of the different data types produced and consumed by the KnowEnG pipelines, we have created Fig 2, which shows exactly that information for all pipelines simultaneously, as well as how each case study makes use of specific data files to perform a multi-pipeline analysis.
3. In order to answer the questions of what analysis runs are possible, we have created the new subsection of the Discussion ("Applications to other biological domains"), which argues how each pipeline supports a large number of possible analysis scenarios in several different biological contexts in multiple species available in KnowEnG.
4. To address what advantages the platform has with respect to other online tools, we have expanded the Discussion by creating the new subsection "Comparison with existing frameworks".
5. Finally, to make it easier to know how to run different analysis routines on user datasets, we have summarized important resources applicable for all the different KnowEnG access modalities in Supp. Table SN2.ST1. These resources include data preparation and quickstart guides, YouTube tutorials, instructions for recreating the case studies in the platform, details about the contents of the Knowledge Network, and links to the repositories that contain the open source code and Docker images. This table is copied below for the editor's convenience.

| Resource Description | Resource URL |
|---|---|
| Pipelines in Public Platform | https://knoweng.org/analyze |
| Pipelines in Seven Bridges CGC | https://cgc.sbgenomics.com/public/apps#q?search=knoweng |
| Pipelines in Jupyter Notebooks | https://knowtebook.knoweng.org/ |
| Platform QuickStart Guides | https://knoweng.org/quick-start/ |
| YouTube Videos | https://www.youtube.com/channel/UCjyIlolCaZIGtZC20XLBOyg |
| TCGA Analysis Walkthrough | https://github.com/KnowEnG/quickstart-demos/tree/master/publication_data/blatti_et_al_2019/ |
| Data Preparation Guide | https://github.com/KnowEnG/quickstart-demos/blob/master/pipeline_readmes/README-DataPrep.md |
| Knowledge Network Summary | https://knoweng.org/kn-data-references/ |
| Knowledge Network Downloads | https://github.com/KnowEnG/KN_Fetcher/blob/master/Contents.md |

| | |
|---|---|
| Docker Images | https://hub.docker.com/u/knowengdev/ |
| GitHub Code | https://knoweng.github.io/ |
| Contact us with Questions or Feedback | knoweng-support@illinois.edu |

## REFERENCES


1. Afgan E, Baker D, Batut B, van den Beek M, Bouvier D, Cech M, et al. The Galaxy platform for accessible, reproducible and collaborative biomedical analyses: 2018 update. Nucleic Acids Res. 2018;46(W1):W537-W44. doi: 10.1093/nar/gky379. PubMed PMID: 29790989; PubMed Central PMCID: PMCPMC6030816.
2. Birger C, Hanna M, Salinas E, Neff J, Saksena G, Livitz D, et al. FireCloud, a scalable cloud-based platform for collaborative genome analysis: Strategies for reducing and controlling costs. bioRxiv. 2017:209494.
3. Anderson C. AZ Partners with DNAnexus for 2 Million Patient Sequencing Project. Clinical OMICs. 2017;4(4):32-.
4. Gao J, Aksoy BA, Dogrusoz U, Dresdner G, Gross B, Sumer SO, et al. Integrative analysis of complex cancer genomics and clinical profiles using the cBioPortal. Sci Signal. 2013;6(269):pl1. doi: 10.1126/scisignal.2004088. PubMed PMID: 23550210; PubMed Central PMCID: PMCPMC4160307.
5. Jensen MA, Ferretti V, Grossman RL, Staudt LM. The NCI Genomic Data Commons as an engine for precision medicine. Blood. 2017;130(4):453-9. doi: 10.1182/blood-2017-03-735654. PubMed PMID: 28600341; PubMed Central PMCID: PMCPMC5533202.
6. Reich M, Liefeld T, Gould J, Lerner J, Tamayo P, Mesirov JP. GenePattern 2.0. Nat Genet. 2006;38(5):500-1. doi: 10.1038/ng0506-500. PubMed PMID: 16642009.
7. Baker EJ, Jay JJ, Bubier JA, Langston MA, Chesler EJ. GeneWeaver: a web-based system for integrative functional genomics. Nucleic acids research. 2011;40(D1):D1067-D76.
8. Warde-Farley D, Donaldson SL, Comes O, Zuberi K, Badrawi R, Chao P, et al. The GeneMANIA prediction server: biological network integration for gene prioritization and predicting gene function. Nucleic Acids Res. 2010;38(Web Server issue):W214-20. doi: 10.1093/nar/gkq537. PubMed PMID: 20576703; PubMed Central PMCID: PMCPMC2896186.
9. Qu K, Garamszegi S, Wu F, Thorvaldsdottir H, Liefeld T, Ocana M, et al. Integrative genomic analysis by interoperation of bioinformatics tools in GenomeSpace. Nature methods. 2016;13(3):245.
10. Floratos A, Smith K, Ji Z, Watkinson J, Califano A. geWorkbench: an open source platform for integrative genomics. Bioinformatics. 2010;26(14):1779-80. doi: 10.1093/bioinformatics/btq282. PubMed PMID: 20511363; PubMed Central PMCID: PMCPMC2894520.
11. Margolin AA, Nemenman I, Basso K, Wiggins C, Stolovitzky G, Dalla Favera R, et al., editors. ARACNE: an algorithm for the reconstruction of gene regulatory networks in a mammalian cellular context. BMC bioinformatics; 2006: BioMed Central.
12. Lefebvre C, Rajbhandari P, Alvarez MJ, Bandaru P, Lim WK, Sato M, et al. A human B-cell interactome identifies MYB and FOXM1 as master regulators of proliferation in germinal centers. Molecular systems biology. 2010;6(1).
13. Alvarez MJ, Shen Y, Giorgi FM, Lachmann A, Ding BB, Ye BH, et al. Functional characterization of somatic mutations in cancer using network-based inference of protein activity. Nature genetics. 2016;48(8):838.
14. Wang K, Saito M, Bisikirska BC, Alvarez MJ, Lim WK, Rajbhandari P, et al. Genome-wide identification of post-translational modulators of transcription factor activity in human B cells. Nature biotechnology. 2009;27(9):829.
15. Hofree M, Shen JP, Carter H, Gross A, Ideker T. Network-based stratification of tumor mutations. Nat Methods. 2013;10(11):1108-15. doi: 10.1038/nmeth.2651. PubMed PMID: 24037242; PubMed Central PMCID: PMCPMC3866081.
16. Yu G, Yu X, Wang J. Network-aided Bi-Clustering for discovering cancer subtypes. Scientific reports. 2017;7(1):1046.

17. Liu Y, Gu Q, Hou JP, Han J, Ma J. A network-assisted co-clustering algorithm to discover cancer subtypes based on gene expression. BMC bioinformatics. 2014;15(1):37.
18. Wu L, Liu Z, Xu J, Chen M, Fang H, Tong W, et al. NETBAGs: a network-based clustering approach with gene signatures for cancer subtyping analysis. Biomarkers in medicine. 2015;9(11):1053-65.
19. Hou JP, Ma J. DawnRank: discovering personalized driver genes in cancer. Genome medicine. 2014;6(7):56.
20. Bourdakou MM, Athanasiadis EI, Spyrou GM. Discovering gene re-ranking efficiency and conserved gene-gene relationships derived from gene co-expression network analysis on breast cancer data. Scientific reports. 2016;6:20518.
21. Cho A, Shim JE, Kim E, Supek F, Lehner B, Lee I. MUFFINN: cancer gene discovery via network analysis of somatic mutation data. Genome biology. 2016;17(1):129.
22. Valentini G, Paccanaro A, Caniza H, Romero AE, Re M. An extensive analysis of disease-gene associations using network integration and fast kernel-based gene prioritization methods. Artificial Intelligence in Medicine. 2014;61(2):63-78.
23. Erten S, Bebek G, Koyutürk M. Vavien: an algorithm for prioritizing candidate disease genes based on topological similarity of proteins in interaction networks. Journal of computational biology. 2011;18(11):1561-74.
24. Guo H, Dong J, Hu S, Cai X, Tang G, Dou J, et al. Biased random walk model for the prioritization of drug resistance associated proteins. Scientific reports. 2015;5:10857.
25. Zhang W, Ota T, Shridhar V, Chien J, Wu B, Kuang R. Network-based survival analysis reveals subnetwork signatures for predicting outcomes of ovarian cancer treatment. PLoS computational biology. 2013;9(3):e1002975.
26. Xu H, Moni MA, Liò P. Network regularised Cox regression and multiplex network models to predict disease comorbidities and survival of cancer. Computational biology and chemistry. 2015;59:15-31.
27. Kim S, Pan W, Shen X. Network-based penalized regression with application to genomic data. Biometrics. 2013;69(3):582-93.
28. Blatti C, Sinha S. Characterizing gene sets using discriminative random walks with restart on heterogeneous biological networks. Bioinformatics. 2016;32(14):2167-75. doi: 10.1093/bioinformatics/btw151. PubMed PMID: 27153592; PubMed Central PMCID: PMCPMC4937193.
29. Dong X, Hao Y, Wang X, Tian W. LEGO: a novel method for gene set over-representation analysis by incorporating network-based gene weights. Scientific reports. 2016;6:18871.
30. Di Lena P, Martelli PL, Fariselli P, Casadio R. NET-GE: a novel NETwork-based Gene Enrichment for detecting biological processes associated to Mendelian diseases. BMC genomics. 2015;16(8):S6.
31. Alexeyenko A, Lee W, Pernemalm M, Guegan J, Dessen P, Lazar V, et al. Network enrichment analysis: extension of gene-set enrichment analysis to gene networks. BMC bioinformatics. 2012;13(1):226.
32. Cornish AJ, Markowetz F. SANTA: quantifying the functional content of molecular networks. PLoS computational biology. 2014;10(9):e1003808.
33. Mostafavi S, Morris Q. Combining many interaction networks to predict gene function and analyze gene lists. Proteomics. 2012;12(10):1687-96.
34. Wang S, Cho H, Zhai C, Berger B, Peng J. Exploiting ontology graph for predicting sparsely annotated gene function. Bioinformatics. 2015;31(12):i357-i64.
35. Pena-Castillo L, Tasan M, Myers CL, Lee H, Joshi T, Zhang C, et al. A critical assessment of Mus musculus gene function prediction using integrated genomic evidence. Genome Biol. 2008;9 Suppl 1:S2. doi: 10.1186/gb-2008-9-s1-s2. PubMed PMID: 18613946; PubMed Central PMCID: PMCPMC2447536.

36. Emad A, Cairns J, Kalari KR, Wang L, Sinha S. Knowledge-guided gene prioritization reveals new insights into the mechanisms of chemoresistance. Genome Biol. 2017;18(1):153. doi: 10.1186/s13059-017-1282-3. PubMed PMID: 28800781; PubMed Central PMCID: PMCPMC5554409.
37. Barretina J, Caponigro G, Stransky N, Venkatesan K, Margolin AA, Kim S, et al. The Cancer Cell Line Encyclopedia enables predictive modelling of anticancer drug sensitivity. Nature. 2012;483(7391):603-7. doi: 10.1038/nature11003. PubMed PMID: 22460905; PubMed Central PMCID: PMCPMC3320027.
38. Kocher SD, Ayroles JF, Stone EA, Grozinger CM. Individual variation in pheromone response correlates with reproductive traits and brain gene expression in worker honey bees. PloS one. 2010;5(2):e9116.
39. Wytock TP, Motter AE. Predicting growth rate from gene expression. Proceedings of the National Academy of Sciences. 2019;116(2):367-72.
40. Kapheim KM, Pan H, Li C, Salzberg SL, Puiu D, Magoc T, et al. Genomic signatures of evolutionary transitions from solitary to group living. Science. 2015;348(6239):1139-43.
41. Nussbaumer T, Wagner C, Heidari P. TraitCorr - correlating gene expression measurements with phenotypic data. bioRxiv. 2019.
42. Costa-Silva J, Domingues D, Lopes FM. RNA-Seq differential expression analysis: An extended review and a software tool. PloS one. 2017;12(12):e0190152.
43. Robinson MD, McCarthy DJ, Smyth GK. edgeR: a Bioconductor package for differential expression analysis of digital gene expression data. Bioinformatics. 2010;26(1):139-40.
44. Huber W, Carey VJ, Gentleman R, Anders S, Carlson M, Carvalho BS, et al. Orchestrating high-throughput genomic analysis with Bioconductor. Nature methods. 2015;12(2):115.
45. Lightbody G, Haberland V, Fiona B, Taggart L, Zheng H, Parks E, et al. Review of Applications of High-Throughput Sequencing in Personalised Medicine: Barriers and Facilitators of Future Progress in Research and Clinical Application. Briefings in bioinformatics. 2018:bby051.
46. Curtis C, Shah SP, Chin S-F, Turashvili G, Rueda OM, Dunning MJ, et al. The genomic and transcriptomic architecture of 2,000 breast tumours reveals novel subgroups. Nature. 2012;486(7403):346.
47. Wang W-H, Xie T-Y, Xie G-L, Ren Z-L, Li J-M. An Integrated Approach for Identifying Molecular Subtypes in Human Colon Cancer Using Gene Expression Data. Genes. 2018;9(8):397.
48. Søkilde R, Persson H, Ehinger A, Pirona AC, Fernö M, Hegardt C, et al. Refinement of breast cancer molecular classification by miRNA expression profiles. BMC genomics. 2019;20(1):503.
49. Kuijjer ML, Paulson JN, Salzman P, Ding W, Quackenbush J. Cancer subtype identification using somatic mutation data. British journal of cancer. 2018;118(11):1492.
50. Väremo L, Henriksen TI, Scheele C, Broholm C, Pedersen M, Uhlén M, et al. Type 2 diabetes and obesity induce similar transcriptional reprogramming in human myocytes. Genome medicine. 2017;9(1):47.
51. Whitfield CW, Cziko A-M, Robinson GE. Gene expression profiles in the brain predict behavior in individual honey bees. Science. 2003;302(5643):296-9.
52. Narusaka M, Abe H, Kobayashi M, Kubo Y, Kawai K, Izawa N, et al. A model system to screen for candidate plant activators using an immune-induction system in Arabidopsis. Plant Biotechnology. 2006;23(3):321-7.
53. Subramanian A, Tamayo P, Mootha VK, Mukherjee S, Ebert BL, Gillette MA, et al. Gene set enrichment analysis: a knowledge-based approach for interpreting genome-wide expression profiles. Proc Natl Acad Sci U S A. 2005;102(43):15545-50. doi: 10.1073/pnas.0506580102. PubMed PMID: 16199517; PubMed Central PMCID: PMCPMC1239896.

54. Huang da W, Sherman BT, Lempicki RA. Systematic and integrative analysis of large gene lists using DAVID bioinformatics resources. Nat Protoc. 2009;4(1):44-57. doi: 10.1038/nprot.2008.211. PubMed PMID: 19131956.
55. Chen EY, Tan CM, Kou Y, Duan Q, Wang Z, Meirelles GV, et al. Enrichr: interactive and collaborative HTML5 gene list enrichment analysis tool. BMC Bioinformatics. 2013;14:128. doi: 10.1186/1471-2105-14-128. PubMed PMID: 23586463; PubMed Central PMCID: PMCPMC3637064.
56. Saul MC, Blatti C, Yang W, Bukhari SA, Shpigler HY, Troy JM, et al. Cross-species systems analysis of evolutionary toolkits of neurogenomic response to social challenge. Genes, Brain and Behavior. 2019;18(1):e12502.
57. Hoadley KA, Yau C, Wolf DM, Cherniack AD, Tamborero D, Ng S, et al. Multiplatform analysis of 12 cancer types reveals molecular classification within and across tissues of origin. Cell. 2014;158(4):929-44. doi: 10.1016/j.cell.2014.06.049. PubMed PMID: 25109877; PubMed Central PMCID: PMCPMC4152462.
58. Cancer Genome Atlas Research N, Analysis Working Group: Asan U, Agency BCC, Brigham, Women's H, Broad I, et al. Integrated genomic characterization of oesophageal carcinoma. Nature. 2017;541(7636):169-75. doi: 10.1038/nature20805. PubMed PMID: 28052061; PubMed Central PMCID: PMCPMC5651175.
59. Shannon P, Markiel A, Ozier O, Baliga NS, Wang JT, Ramage D, et al. Cytoscape: a software environment for integrated models of biomolecular interaction networks. Genome research. 2003;13(11):2498-504.
60. Pratt D, Chen J, Welker D, Rivas R, Pillich R, Rynkov V, et al. NDEx, the network data exchange. Cell systems. 2015;1(4):302-5.
61. Gentleman RC, Carey VJ, Bates DM, Bolstad B, Dettling M, Dudoit S, et al. Bioconductor: open software development for computational biology and bioinformatics. Genome biology. 2004;5(10):R80.
62. Merkel D. Docker: lightweight linux containers for consistent development and deployment. Linux Journal. 2014;2014(239):2.
