# Supplementary material for: Knowledge-guided analysis of "omics" data using the KnowEnG cloud platform
Source: PLoS Biol. 2020 Jan 23;18(1):e3000583. doi: 10.1371/journal.pbio.3000583 (PMC6977717; doi:10.1371/journal.pbio.3000583)
Supplement: Attachment — Submitted filename: Response_to_Reviewers.rev2.pdf [file pbio.3000583.s010.pdf]

RESPONSE TO REVIEWER #2

Comment: I appreciate the authors response to my comments. In particular, the description of how KnowEng fits in with other platforms is very useful, and I think significantly improves the paper.

I think it is clear that KnowEng is distinct from these other platforms. But it seems different mostly in degree, not in kind, and it is still not clear how big of a need it fills. "Knowledge-guided analysis" is not something I have heard used before, and I am not sure how big of a field it is (I could not tell if this is a new term defined to describe KnowEng, or if it was a pre-existing gap for a lot of users).

Response: We are grateful for the suggestions from the reviewer that led to our more careful description of how KnowEnG fits into the landscape of tools that exist for downstream analysis of omics data. Although we only cited around twenty example publications that use known or externally constructed networks of gene or protein interactions in the analysis of genomic or transcriptomic data, there is a much wider body of literature that meet these criteria. These methods are what we refer to when we discuss “knowledge-guided analysis”. Some of the difficulties that prevent these methods from wider adoption is the fact that they 1) are often developed using only a single specific knowledge network or 2) pertain to only a single step of downstream analysis. We built the KnowEnG platform to enable users of these methods to very easily launch them with many different options for the type of guiding knowledge network and to seamlessly insert these advanced methods among the steps of common downstream analysis workflows.

Comment: The example use cases are fine. But they are too detailed for somebody outside of the field of cancer genomic to really grasp the big picture. On the other hand, the high-level descriptions of "clustering" or "gene prioritization" are so broad that it's hard to see what is provided by KnowEng. It seems like the right level of detail would be somewhere in between, where a broad need were motivated and then addressed. I think the long responses to my comments illustrate the challenges in explaining the value of a system like this to somebody who has not developed it or used it closely.

In short, while I see the potential value and novelty of the platform, the current manuscript does not make it clear to me that it is the major advance that the abstract claims.

Response: The decision to frame the paper as several cancer-based use cases was a strategic one. We wanted to concretely show how frequently used analyses deployed in high-profile papers could be recreated on the KnowEnG platform. Not only that fact, but additionally, knowledge-guided versions of the analyses were also available and could be easily run with many different definitions of knowledge networks. Finally, we felt the use cases provided us a chance to comment on how the knowledge-guided analysis results differed and led to novel insights. We chose to center on cancer-based use cases because we felt this is a large research community where many projects using knowledge-guided analyses have been published previously. We chose the three different use cases to show that the KnowEnG system can be applied to different omics data types and be deployed on different compute infrastructures. In the Discussion where we detail “Applications to other biological domains”, we are stepping back from the use cases to reiterate how the analysis pipelines (with standard or knowledge guided modes) are fundamental types of omics downstream analyses and appear in applications in human health outside of cancer and in studies of other species (many of which are currently supported in KnowEnG). The advance offered by the KnowEnG system is the flexibility in which knowledge-guided analysis methods can be inserted in common downstream omics analysis workflows.

RESPONSE TO REVIEWER #3

Comment: Some of my concerns are still there, in particular the fact that the tool requires the maintainer to put an effort in including new analysis approaches. However, this is true for most available pipeline managers like KnowEng.

Most of my other comments have been addressed appropriately, and the amount of work that was put in the revision gives me hope that the authors will keep maintaining and developing KnowEng into a more complete framework.

Response: We thank the reviewer for their feedback and their appreciation of the improvements in the revision. We share the reviewer's opinion that the next phase of development for the KnowEnG framework would be to enable developers to add existing and novel additional pipelines without requiring modifications to the platform. We believe that by building our system on technologies such as Docker containers, CWL descriptions, and scalable compute clouds, we have already adopted the fundamental building blocks, and we have shown the feasibility of such an approach by loading and running our analysis pipelines into the externally-developed SevenBridges Cancer Genomics Cloud. We hope to use this publication as an opportunity to develop a larger user base while we pursue additional improvements to the KnowEnG framework.

RESPONSE TO ACADEMIC EDITOR

Comment: My inclination is that you accept on the proviso that they look at what they have, which has probably been expanded considerably in the trying to address all our comments and seek to reduce the work, not because they have to, as you say there is no limit on M&R papers but because it would be better vehicle to advertise their resource - which is clearly what they are trying very hard to achieve.

Response: We thank the academic editor for the suggestion, and we completely understand the concern about the manuscript length. However, after much deliberation and serious consideration, we concluded that a shorter version of the paper will not allow us to clearly state the major strengths and capabilities of the KnowEnG system nor provide readers with additional context in which to consider when the KnowEnG is applicable. In addition, since this manuscript is the flagship publication of KnowEnG, we would like for it to serve as a comprehensive reference for all future publications to cite and shortening it significantly may hinder this goal. As a result, we decided to keep the paper in its comprehensive format and hope that the editorial staff would accept this decision.
